# Seizure recruitment properties are dependent upon dynamotype: A modeling study

**DOI:** 10.1371/journal.pcbi.1013975

**Published:** 2026-09-21

**Authors:** Diana M. Karosas, Marisa Saggio, William C. Stacey

**Affiliations:** 1 Department of Biomedical Engineering, BioInterfaces Institute, University of Michigan, Ann Arbor, Michigan, United States of America; 2 Aix Marseille Univ, INSERM, INS, Inst Neurosci Syst, Marseille, France; 3 Department of Neurology, University of Michigan, Ann Arbor, Michigan, United States of America; University of Birmingham, UNITED KINGDOM OF GREAT BRITAIN AND NORTHERN IRELAND

## Abstract

Seizure propagation – how epileptogenic brain tissue recruits less excitable tissue – is poorly understood. Previous studies have used dynamical modeling to study seizure propagation and to create patient-specific whole-brain models of seizure spread. However, these studies focused on seizures of a single dynamotype (onset and offset bifurcation pair). Here, we implement a novel coupling method to investigate seizure propagation in a diverse array of dynamotypes. We utilize the Multiclass Epileptor, a recently proposed model that captures a wide range of seizure dynamotypes in a cortical mass (“node”). We consider two nodes: the seizure onset zone (node 1), which bursts autonomously, and the potential propagation zone (node 2), which is not independently epileptogenic but can be recruited by node 1. We examine the impact of intrinsic and coupling factors on the likelihood and speed of recruitment, with particular attention to the onset bifurcation of node 1. We also measure the range of onset behaviors observed in node 2 with respect to the onset behavior of node 1. The model predicted that seizures that display baseline shifts at onset are less likely to spread, and spread more slowly, compared to seizures that do not exhibit baseline shifts at onset. Seizures that present with amplitude scaling at onset were unlikely to propagate. Further, the model predicted the potential for unusual combinations of onset dynamics, such as a baseline shift in node 2 but not node 1. We confirmed the possibility for several of these unusual recruitment behaviors in humans using intracranial electroencephalography data. The results of the study provide a theoretical framework for seizure propagation, establishing a basis for innovations in characterization of patients’ seizure networks and identification of the seizure onset zone.

## Author summary

In this work, we examined how a seizure spreads from one part of the brain to another using a computational model. We modeled two brain regions using the Multiclass Epileptor, which reproduces a range of brain activity patterns associated with seizures. In the model, the first brain node was able to recruit the second brain node into a seizure. The model predicted that the likelihood and speed of seizure spread differ depending on the pattern of brain activity observed at the start of the seizure. We also found that the pattern of brain activity at seizure onset is not necessarily the same pattern seen when the seizure spreads. We confirmed this possibility for mismatched patterns in recordings from human brain. The findings of the study improve our understanding of seizure spread, which lays the groundwork for development of tools to quantify seizure spread and may inform future work in patient-specific brain modeling.

## Introduction

Epilepsy, defined as the propensity for multiple seizures [1], affects about 1% of the population, over 65 million people globally [2]. Antiseizure medications are the first-line treatment for epilepsy, but are insufficient to achieve seizure freedom in at least one-third of patients [3,4]. Surgical resection is a treatment option for drug-resistant focal epilepsy; however, between 20% and 60% of individuals continue to experience seizures following resective surgery [3,5]. Resection aims to remove the brain tissue responsible for initiating the seizure, known as the epileptogenic zone [6]. However, seizures typically propagate beyond that tissue once the seizure begins. A contributing factor to ineffective surgical outcomes is a lack of understanding of seizure propagation.

The current gold standard for delineation of the seizure onset and propagation zones is visual intracranial EEG analysis by a clinical expert [7,8]. That determination is primarily based upon identifying the first channels that begin a seizure, known as the seizure onset zone [7]. Clinical reports also describe how the seizure spreads, but such descriptions of seizure propagation are not standardized. Propagation is primarily assessed via patterns of latency (i.e., how long until a channel joins the seizure) and synchronicity (i.e., if the propagated channel is firing in synch with the onset channels). Reliance on visual interpretation restricts the definitive identification of onset, early, and late spread zones, leading to a limited understanding of seizure propagation. Clinicians utilize empirical descriptions to decide when propagation is “early” or “late” and how to interpret such results. A more robust method of quantifying seizure propagation would aid in better characterization of the seizure network.

Better understanding of propagation would benefit from the use of an appropriate model system that is relevant across a range of seizures. Epilepsy is a heterogenous disease, with many etiologies [9] and pathways to seizures, a concept called degeneracy [10]. Likewise, a particular seizure propagation pattern may emerge from a number of underlying pathologies. Research focused on a particular biological mechanism can provide important insights into a specific subset of epilepsies, but few findings generalize across a wider range of heterogeneity of the disease. Yet despite the biological diversity associated with the generation of epileptic seizures, the resulting dynamical mechanisms underlying seizure onset and termination seem to be more limited [11,12]. A complementary approach to epilepsy research is thus to focus on the invariant properties characterizing each dynamical mechanism [11]. One invariant property is how the brain transitions between resting state and seizure [11–13], with commonly proposed dynamical mechanisms including bifurcations and noise-induced transitions [12,14,15]. Work that assumes the former mechanism has shown that there are a limited number of bifurcations governing the transitions between resting and seizing states in human epilepsy (six total in a first approximation; four onset and four offset) [11,16,17]. These bifurcations can be hypothesized from their characteristic timeseries properties and are present across etiologies, brain regions, and species [11,16,18]. They are even observed in experimental conditions that prevent synaptic transmission [11]. Thus, while a focus on invariant properties necessarily sacrifices direct biological connection, the advantage of this approach is universality.

Mathematical models can simulate seizures as systems that undergo bifurcations to enter and exist bursting, which enables in silico study of seizure propagation. Previous studies have used large-scale brain models to investigate seizure propagation and surgical targets, equipping each brain region with a mathematical model to simulate the local dynamics. In these studies, the local dynamics at each brain region have been identical, differing only in connectivity and/or excitability parameter [15,19–25]. Previous studies on coupled brain regions with different onset/offset bifurcations pairs are rare and focus on synchronization rather than recruitment [26]. However, there are four bifurcations each that can be involved in seizure onset and offset, and data compatible with all of these bifurcations were observed in a cohort of 120 patients with focal epilepsy [16]. Seizure dynamotype (onset/offset bifurcation pair) can change from seizure to seizure in a single patient [16] and over the course of epileptogenesis [18]. Modeling work predicts different dynamotypes respond differently to ictal-aborting stimulation [27] and have different synchronization properties [26]. The diversity of dynamotypes in epilepsy and their differential responses to stimulation call for further investigation of the impact of local dynamics on seizure propagation.

The goal of this work is to provide the first exploration of recruitment with a focus on when different brain regions have potentially different intrinsic dynamics. The strategy is to describe recruitment across multiple local dynamics in the active seizure focus and the recruited tissue, with specific attention to conditions in which seizures are more or less likely to propagate. To do so, we extend the Multiclass Epileptor, a mathematical model uniquely capable of reproducing a wide range of dynamotypes [13,28]. We implement a novel method to couple two simulated brain regions, a seizure onset zone and a non-epileptogenic potential propagation zone, in a toy model of propagation. We first analyze the propagation model theoretically, then simulate recruitment under a variety of dynamical conditions. We identify the dynamical conditions under which recruitment is more likely. We also qualitatively describe the potential dynamical behavior of the propagation zone, given the dynamics of the onset zone. We provide examples of several of the different predicted recruitment behaviors via intracranial EEG recordings in patients with epilepsy. The results of the study provide a dynamical framework for seizure propagation, deepening our understanding of recruitment of non-epileptogenic brain tissue during a seizure. Factors that influence recruitment likelihood and speed are identified, providing a basis for future innovations in the characterization of patients’ seizure networks and identification of the seizure onset zone.

## Model and methods

### Ethics statement

All patients gave written consent to have their deidentified EEG data saved for research, and the protocol was approved by the University of Michigan IRB.

### The Multiclass Epileptor

To study the influence of dynamical conditions on seizure propagation, we coupled two nodes of the Multiclass Epileptor, a phenomenological model that can reproduce a wide range of onset and offset bifurcations. To our knowledge, the Multiclass Epileptor is the only model that can describe the relationships between different dynamotypes observed in epilepsy, providing a unique opportunity to examine the influence of dynamical conditions on recruitment. Full model details can be found in [13,28], and we also provide a more comprehensive summary in S1 File. Here, we provide a brief introduction to lay the foundation for our novel coupling method.

Epilepsy is characterized by at least two rhythms: a fast rhythm governs neural activity during a seizure, while a slower process governs the transitions between seizure and resting states. Bursting thus characterized by periods of quiescence and activity is termed ‘fast-slow bursting’ and involves fast and slow subsystems, between which there is timescale separation. A minimally complex realization of the fast subsystem, comprised of two state variables *(x, y)*, is given by, Eqns. 1, 2.


$$\begin{array}{c} \dot{x}=\;-y \end{array}$$ (1)

$$\begin{array}{c} \dot{y}=x^{\text{3}}-\mu_{\text{2}}x-\mu_{\text{1}}-y\left(\nu +x+x^{\text{2}}\right) \end{array}$$ (2)

The state variable *x* is the output of the system, and tracks voltage content during a seizure, the equivalent of an EEG signal. Within the state space (*x, y*) of the model, resting states are represented by stable fixed points, and ictal states are represented by stable limit cycles. The existence of stable fixed points and limit cycles and therefore the behavior of the fast subsystem varies depending on the values of its parameters *(μ*_*1*_*, μ*_*2*_*, ν)*. Although the model is phenomenological, these parameters have a similar dynamical role to the influence of physiological factors such as ion concentrations, oxygen consumption, synaptic activity, etc. [11,16].

As in previous work [13,27], we constrain the parameters of the fast subsystem to the surface of a sphere centered at the origin (Fig 1). Note that the choice of parameter space geometry is arbitrary; only the relationships between regions on the sphere are relevant. It can be demonstrated for this model that the spherical surface captures these relationships well (see S1 File). According to the location of the fast subsystem on the parameter space sphere, the system may be ‘resting only’, ‘ictal only’, or ‘bistable’ with potential for both resting and ictal behavior. In the resting region, we distinguish ‘active rest’ (top right quadrant) from ‘rest’ (all other ‘resting only’ regions) based on the location of the stable fixed point in state space. Two rest-ictal bistability regions, with distinct state space topologies, exist on the surface of the parameter space sphere. In the ‘limit cycle small’ bistability region (LCs), the stable fixed point is separated from the stable limit cycle. Due to this separation, transitions between stable fixed point and limit cycle are associated with baseline shifts in the *x* variable. In the ‘limit cycle big’ bistability region (LCb), the stable fixed point is within the stable limit cycle and transitions between them are not associated with baseline shifts.

**Fig 1:**
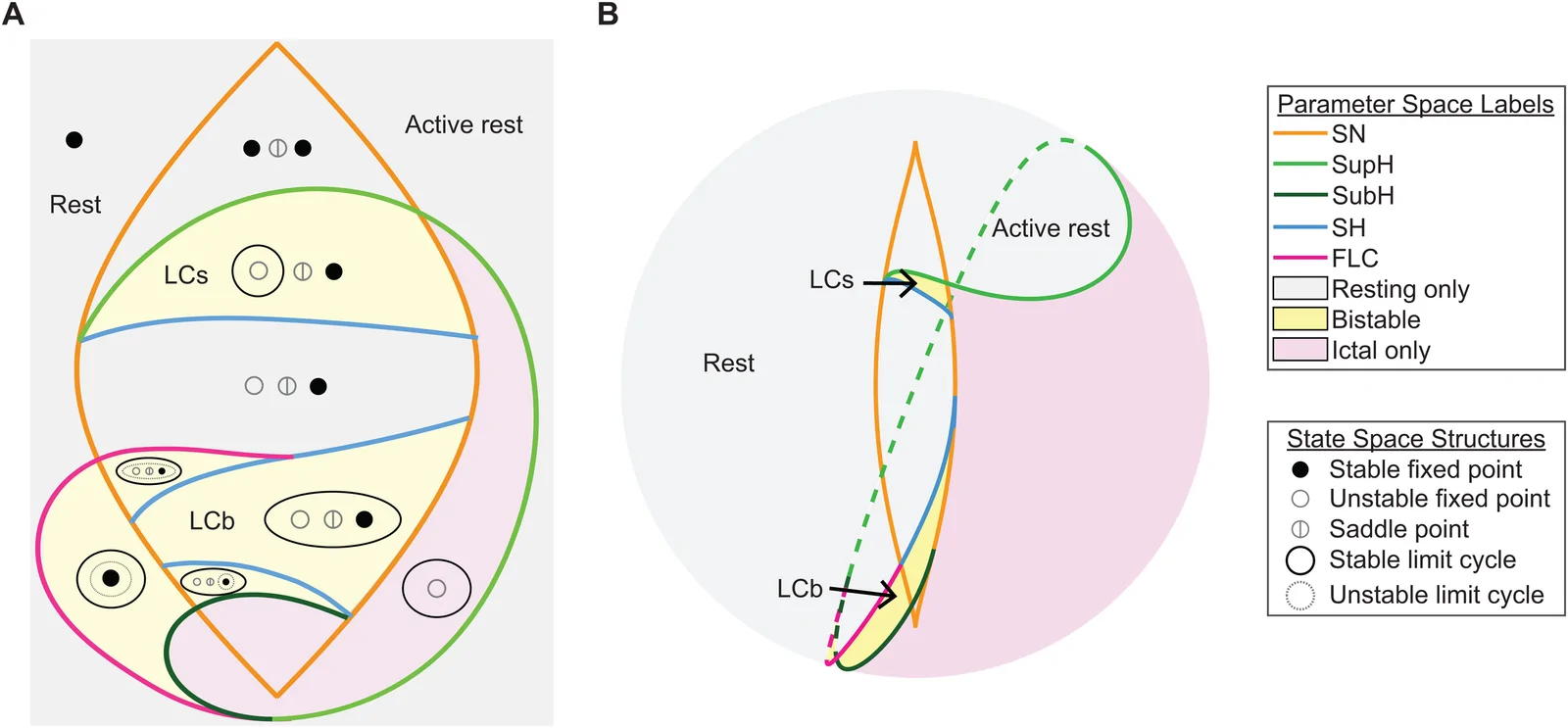
Parameter space of the Multiclass Epileptor. (A) 2D illustration and (B) 2D projection (lambert equal area). Bifurcation curves (colored lines) separate qualitatively distinct behavioral regions (shaded). Dashed lines indicate the back of the sphere in (b). State space diagrams are drawn in each region in (a). ‘Active rest’ includes the ‘resting only’ region to the right of the SN bifurcation in the top half of the sphere; all other ‘resting only’ regions are ‘rest’. Two bistability regions exist: limit cycle small (‘LCs’), where the stable fixed point is outside the limit cycle; and limit cycle big (‘LCb’), where the stable fixed point is inside the limit cycle. SN = saddle node, supH = supercritical Hopf, subH = subcritical Hopf, SH = saddle homoclinic, FLC = fold limit cycle.

Transitions between resting and ictal states in the Multiclass Epileptor can be precipitated by slow changes in the fast subsystem’s parameters that alter the existence of stable fixed points and limit cycles. These types of transitions are known as bifurcations, and they are represented by curves that divide regions of qualitatively distinct behavior repertoires in Fig 1. Over the course of a simulated seizure, the fast subsystem traverses a path that crosses onset and offset bifurcations in parameter space. The slow subsystem moves the fast subsystem along this bursting path. Multiple realizations of the slow subsystem are possible [13,28,29]. For the present work, we modeled the node representing the seizure onset zone using hysteresis-loop paths, in line with the approach used for the Epileptor model [11]. Hysteresis paths exploit bistability in the fast subsystem and feedback among fast and slow variables to produce autonomous bursting. A single implementation was chosen for comparison among bursting paths. For simplicity and without loss of generality, we chose the bursting paths to be arcs of great circles that connect onset and offset bifurcations. Details of the implementation method can be found in [13] and S1 File. The slow dynamics contains an excitability parameter, d*, determining how prone the system is to initiate a seizure.

The specific onset and offset bifurcations of a bursting path determine the dynamotype of the simulated seizure. There are eight dynamotypes that can be implemented on this sphere using the hysteresis mechanism. We separated these eight dynamotypes into four groups by onset bifurcation in this work: saddle node with baseline shift (SN(+DC)), supercritical Hopf (supH), saddle node without baseline shift (SN(-DC)), and subcritical Hopf (subH). The invariant characteristics of these onset bifurcations are indicated in Table 1 [13,16,28].

**Table 1:** Onset bifurcation characteristics.

Bifurcation	Characteristics at bifurcation point		
	Amplitude scaling from zero	Frequency	Baseline shift in x
Saddle node with baseline shift (SN(+DC))	no	nonzero	yes
Supercritical Hopf (supH)	yes	nonzero	no
Saddle node without baseline shift (SN(-DC))	no	nonzero	no
Subcritical Hopf (subH)	no	nonzero	no

### Connecting seizure onset and propagation zones

The Multiclass Epileptor describes the behavior of a cortical mass or “node”. Although the model describes seizure features that are present at different scales [11], in this work, it may be helpful to think of a node as the mass under a single SEEG electrode contact. The characteristic properties of bifurcations can be observed in the local field potential produced by this tissue. To study seizure propagation, we coupled two nodes of the model.

Clinical observations guided the development of our coupling method. When a seizure propagates, the propagation zone often exhibits the same onset pattern as and synchronizes with the seizure onset zone. However, the propagation zone does not always adopt the same dynamics as the seizure onset zone, and seizures often propagate to brain tissue that is not independently epileptic. The latter point is evidenced by seizure freedom after resective surgery of the seizure onset zone in patients that experience seizures with focal onset followed by generalization. We could think of two parameter space possibilities to capture these tendencies. First, the highly excitable onset zone and less excitable propagation zone could inhabit similar regions of parameter space, and the onset zone could then influence the excitability of the propagation zone. Second, they could exist in different regions of parameter space, and the onset zone could move the propagation zone into its region of parameter space during a seizure. The former explanation would require every node in the brain to inhabit the same bistable region of parameter space. In such a case, all brain nodes would have similar underlying dynamics and different excitabilities. This is the standard approach used in large-scale seizure network models [23–25,30–32]. Only the latter explanation provides a mechanism for recruitment of the propagation zone from multiple regions of parameter space, including resting-only regions. Since we are interested in this heterogenous scenario, we chose to explore the latter case. Thus, in our coupling method, we assume that a recruiting brain region changes the dynamics of the propagation zone to be similar to its own, but that the propagation zone is not required to initially be in the same region of parameter space.

Node 1 represented the seizure onset zone and was modeled as a hysteresis-loop burster. Since we were interested in the initial recruitment of tissue into a seizure (and not complex feedback effects after a seizure starts such as suppression or patterns of seizure progression and termination), we implemented one-directional coupling. In other words, node 2 was affected by node 1 but node 1 was not influenced by node 2. While this is an oversimplification, it captures the underlying pathology of seizure onset activity, defined by when uncontrolled bursting overrides normal brain activity. Node 2 represented the potential propagation zone and was not independently epileptogenic. The fast subsystem of node 2 was unchanged from Eqns. 1–2. The slow subsystem was modified to allow the fast subsystem of node 1 to drive recruitment of node 2. Fast-to-slow coupling has been proposed as a mechanism for seizure propagation since it allows fast activity in the seizure focus to influence the slow variables in another brain region [23,24,32]. Since node 2 does not burst autonomously and only moves toward seizure onset according to input from its coupling with node 1, node 2’s bursting mechanism is effectively a slow-wave type [29].

In the propagation model, node 2 is initially placed on a stable fixed point in a resting-only or bistable region of parameter space and does not move autonomously. Upon the onset of the burst in node 1, node 2 begins to move toward node 1 in parameter space along an arc connecting the nodes, with speed proportional to the square of the difference between the current state of node 1 and its resting state (in *x*). Since the *x* variable mimics a voltage recording, this form of coupling captures the influence of the change in voltage in the seizure onset zone (node 1) on the activity in the potential propagation zone (node 2). During the recruitment period, we modeled node 2’s speed using Eqn. 3, where *G* is a coupling strength constant, *x*_*1*_ is the value of the *x* variable for node 1, and *x*_*1, rest*_ is the location of the fixed point for node 1. *x*_*1*_ and *x*_*1, rest*_ are functions of time.


$$\begin{array}{c} {\dot{z}}_{\text{2}}={G(x_{\text{1}}-x_{\text{1},\;rest})}^{\text{2}} \end{array}$$ (3)

Following the offset of the burst in node 1, node 2 moves back to its initial location in parameter space at a constant speed. That node 2 has an innate tendency to return to its initial location in parameter space is supported by the clinical observation that seizures tend to follow a stereotyped progression in a given patient. If propagation zones did not return to their “preferred” parameters, then we would expect each seizure to propagate differently. We implemented a simple method to return node 2 to its initial location in parameter space; however, note that we focus on onset only and none of our analyses were dependent on post-coupling trajectories.

A representative example of recruitment showing the parameter space movement of the nodes and corresponding model outputs is provided in Fig 2 and S3 File. The parameterization of node 2’s path in parameter space and all system equations for node 1 and node 2 are provided in S1 File.

**Fig 2:**
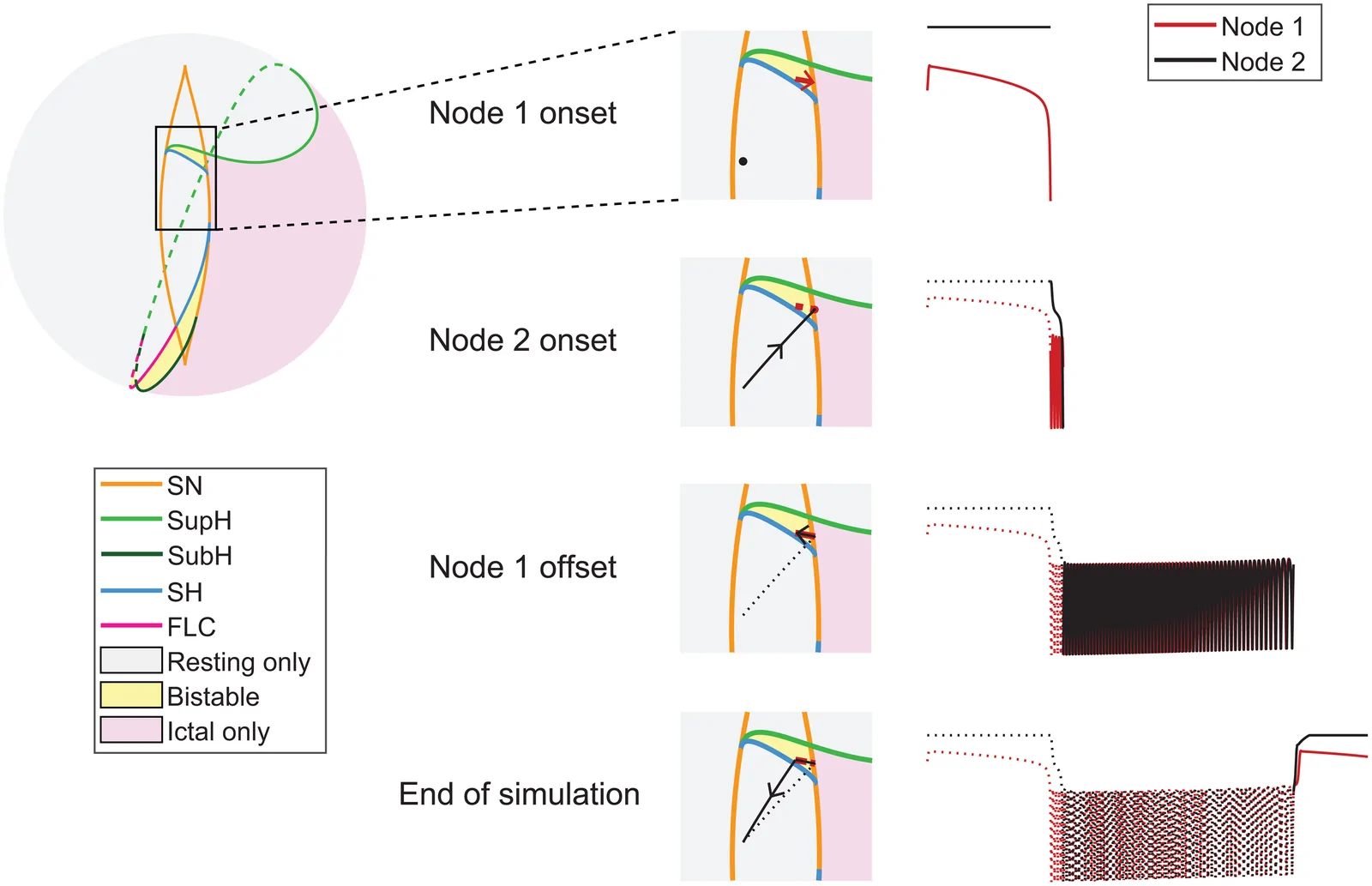
Example of recruitment of node 2 by node 1. Parameter space movement (left) and model output (right) are shown for each node. Node 1 begins to burst in the LCs region, while node 2 is far away in a resting-only region. During the burst, node 1 pulls node 2 towards itself, and node 2 starts to burst when it crosses the onset bifurcation curve. Results are shown at four time points in the simulation (top to bottom). In each timeseries, solid lines indicate activity since the last time point. SN = saddle node, supH = supercritical Hopf, subH = subcritical Hopf, SH = saddle homoclinic, FLC = fold limit cycle.

### Simulation parameters

We simulated a wide range of node 1 classes, node 2 initial locations, coupling strength (*G*) values, and node 1 excitability (d*) values to investigate the effects of these factors on recruitment properties. Details of the simulation parameter sweeps can be found in S1 File. Briefly, for each of the eight dynamotype classes that can be implemented via hysteresis-loop bursting, three paths were chosen arbitrarily. A total of 71 node 2 locations were sampled from throughout parameter space, with greater attention to regions near bifurcations. Since node 2 was initially placed in the quiescent state (on a stable fixed point) to model a potential propagation zone, the ictal-only region of the sphere was avoided. Coupling strength and excitability were varied across a range that included combinations resulting in very low to very high overall recruitment. Increasing node 1 excitability increased the amount of time node 1 spent near its onset bifurcation, relative to the total burst duration. There were a total of 86,904 simulations. For all simulations, the model was solved numerically using Euler’s forward method and a timestep of 0.01. Initial conditions were chosen such that the nodes were initially at rest in *(x, y)* state space. Timeseries were generated from the state variable *x*. All simulations lasted for a single burst in node 1. MATLAB R2024b was used for all simulations.

### Recruitment analysis

Three recruitment properties were measured: the proportion of simulations that resulted in recruitment, recruitment delay, and recruitment energy. For each simulation, whether node 2 was recruited was identified via thresholding (details in S1 File). Recruitment delay was defined as the time between node 1 onset and node 2 onset. Recruitment energy was defined as the area under the recruitment term *G(x*_*1*_ *– x*_*1, rest*_*)*^*2*^, measured from node 1 onset until node 2 onset. Recruitment energy captures the amplitude and duration of bursting in node 1 required to recruit node 2. For delay and energy calculations, time was divided by the duration of the burst in node 1 for comparison across node 1 dynamotypes and excitability values. This time normalization prevented delay and energy values from being elevated for slower or longer bursting paths.

Results were presented with respect to node 1 onset bifurcation. There were four onset bifurcation groups: SN (+DC), supH, SN(-DC), and subH. To evaluate the effect of propagation zone dynamics on recruitment properties, analyses were repeated with respect to node 2 locations. Except where otherwise specified, the mean and variance of each measure were computed for each node 2 location, then normalized by subtracting the minimum and dividing by the range for visualization of trends. Statistical significance in recruitment properties between groups was evaluated via Kruskal-Wallis tests followed by multiple comparisons with Bonferroni correction, if appropriate.

### Clinical data

Data were collected from a previously-deidentified database at the University of Michigan of long-term intracranial video-EEG monitoring in patients with epilepsy. The sampling rate was 4096 Hz. Seizure onset and propagation zones were identified by reading the official EEG report written by the treating clinicians. We identified recordings compatible with specific onset bifurcations visually: SN(+DC) onsets were identified via baseline shifts and supH onsets were identified via amplitude increasing from zero. The remaining recordings, i.e., those with arbitrary amplitude, were marked as SN(-DC) or subH onsets. It needs to be stressed that such analysis of shifts and amplitude scaling laws does not prove that a bifurcation is occurring, but only that the data are compatible with this scenario [29]. SN(-DC) and subH onsets, lacking specific features, are more susceptible to mislabeling. For consistency with the definition of burst onset used in our simulations (see S1 File), we specifically considered the dynamics in the onset channel at the onset of *sustained* oscillations.

## Results

### Theoretical analysis

The Multiclass Epileptor is a dynamically rich, well-understood model of bursting that enables theoretical analysis. Here, using the parameter and state space topologies of the Multiclass Epileptor as a guide, we identify possible mechanisms of recruitment of node 2. We then predict dynamical conditions that encourage recruitment, and the behavior of node 2 when it is recruited. Note that while this analysis is provided in the context of coupled nodes of the Multiclass Epileptor, our approach and the recruitment mechanisms identified are applicable to other dynamical models of bursting.

#### Possible mechanisms of recruitment.

In this work, all node 1 bursting paths are located within bistability regions to facilitate autonomous bursting. Node 1 can enter bursting via SN(+DC) or supH bifurcation in LCs and via SN(-DC) or subH bifurcation in LCb (Fig 3A, left). Note in Fig 3A that only certain portions of the SN and supH bifurcation curves (those that are highlighted) can induce bursting.

**Fig 3:**
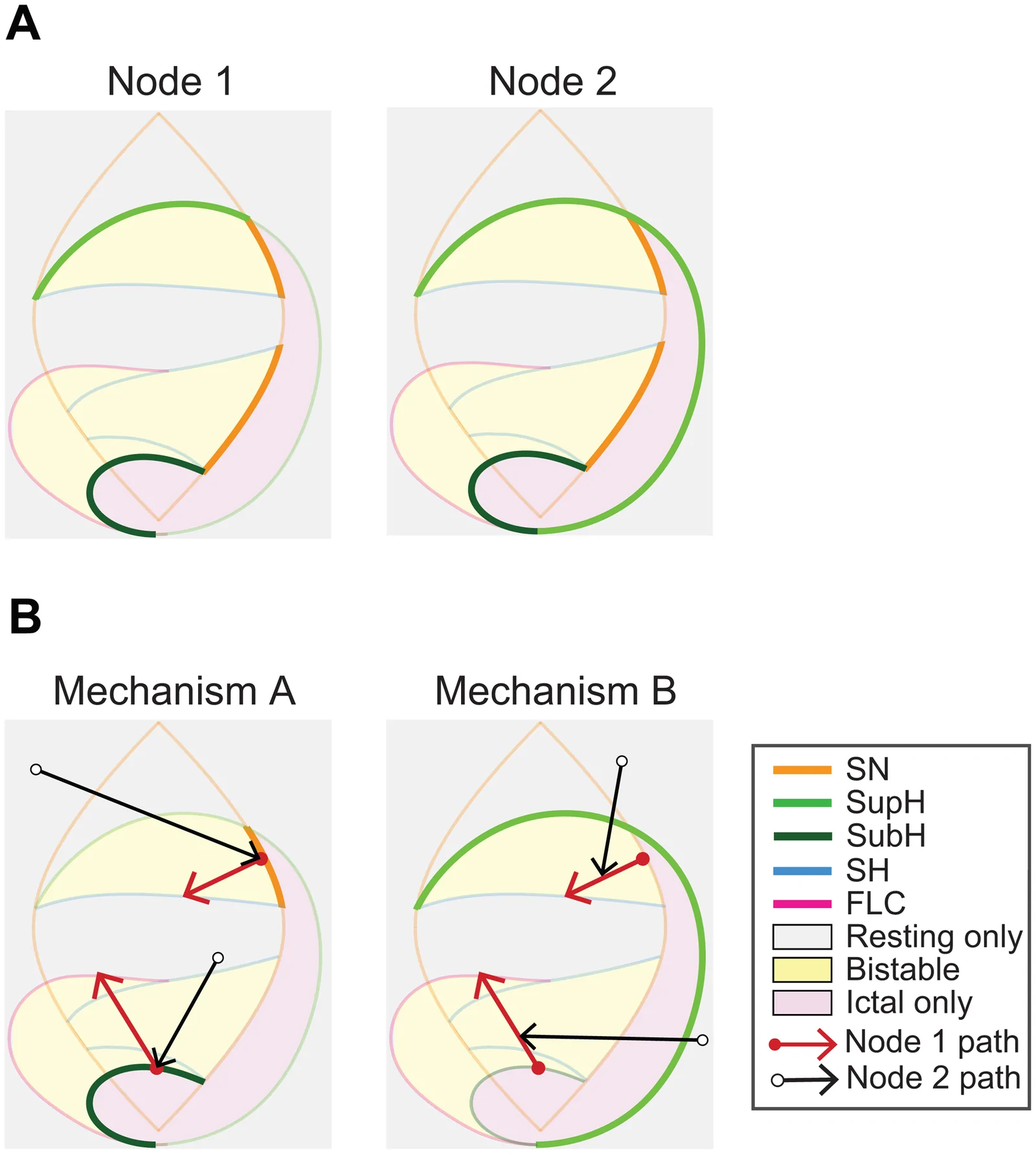
Mechanisms of recruitment. (A) Illustration of potential onset bifurcations for node 1 (left) and node 2 (right). Bifurcations curves (or parts of bifurcation curves) that can serve as onset bifurcations are highlighted. (B) Illustration of two mechanisms of recruitment. Onset bifurcations for node 2 are highlighted. In mechanism A (left), node 2 (black) reaches node 1 (red) before node 1 leaves its onset bifurcation. In mechanism B (right), node 2 is recruited by crossing a bifurcation before reaching node 1. SN = saddle node, supH = supercritical Hopf, subH = subcritical Hopf, SH = saddle homoclinic, FLC = fold limit cycle.

Node 2 may initially be located anywhere in parameter space with the exception of the ictal-only region. Upon the onset of coupling, node 2 travels toward either the LCs or LCb bistability region, depending on the class of node 1. Two mechanisms exist by which node 2 can enter bursting (Fig 3B). In mechanism A, node 2 does not cross onset bifurcations before reaching node 1 and reaches node 1 before node 1 has left the proximity of its onset bifurcation. Node 2 can be recruited if node 1’s onset bifurcation is SN or subH since these bifurcations are embedded in the ictal-only region. Crossing these onset bifurcations destabilizes the stable fixed point (i.e., resting state) for node 2, leading to recruitment. Recruitment through mechanism A must occur early in the burst in node 1. If node 2 reaches node 1 after node 1 leaves its onset bifurcation, then node 2’s resting state will not be destabilized, and node 2 will follow node 1 in parameter space without leaving the stable fixed point in state space. Even if the two nodes are in the same location in parameter space, node 1 will seize and node 2 will not seize because of bistability. Except in cases where the initial parameters of node 2 are very close to the onset bifurcation of node 1, mechanism A requires node 2 to temporarily break timescale separation, meaning the slow variable *z* is effectively moving at the same time scale as *x*. Mechanism A is more likely to occur when the coupling strength (*G*) is high, which increases the speed of node 2’s movement toward node 1, and when node 1 excitability (d*) is high, which increases the relative amount of time node 1 spends near onset bifurcation after having crossed it. Examples of recruitment through mechanism A are given in Fig 3B, left.

In mechanism B, node 2 is recruited by crossing an onset bifurcation along the path to, but before reaching, node 1. We can think of two scenarios for mechanism B. First, node 2 may pass through the ictal-only region along the path to node 1. The portion of the supH bifurcation that borders the ictal-only region can thus act as an onset bifurcation for node 2. Second, for some initial node 2 parameters, crossing the supH bifurcation to enter the LCs region promotes recruitment (see “Theoretical predictions” below). In contrast to mechanism A, mechanism B can facilitate recruitment at any point in the burst in node 1 because it is a bifurcation along the route to node 1 that destabilizes node 2’s stable fixed point. Since the window for recruitment is longer in mechanism B, node 2 can be recruited through mechanism B at slower speeds than through mechanism A. Given that mechanism B does not rely on reaching node 1 before node 1 leaves its onset bifurcation, recruitment through mechanism B should also be less sensitive to coupling strength (*G*) and node 1 excitability (d*). Examples of recruitment through mechanism B are given in Fig 3B, right. Considering both recruitment mechanisms, the onset bifurcations that can induce bursting in node 2 include all those that can induce bursting in node 1, plus the portion of the supH bifurcation bordering the ictal-only region (Fig 3A, right).

#### Theoretical predictions.

Three predictions arise from the preceding analysis. First, under equal coupling strength, nodes in the LCb bistability region will recruit node 2s from more initial locations than nodes in the LCs bistability region. The onset bifurcation curves in the LCb bistability region are fully embedded in the ictal-only region, whereas only the SN(+DC) bifurcation in the LCs bistability region borders the ictal-only region (Figs 1, 3A). The consequences of this topology are twofold: (1) supH-onset node 1 dynamotypes, which occur in LCs, cannot recruit through mechanism A and (2) node 2 is more likely to encounter the ictal-only region when approaching the LCb bistability region (mechanism B). Thus, recruitment through either identified mechanism is more likely when node 1 is in the LCb bistability region.

Second, nodes in active rest are more likely to be recruited than nodes in rest for a given combination of node 1 dynamotype and coupling strength. Fig 4 illustrates state space arguments for higher recruitment proportions when node 1 is in LCs and node 2 is in active rest compared to rest. If node 2 is initially in rest (Fig 4B, top two panels), it may enter LCs through the region above the supH bifurcation (region II in Fig 4) or the region below the SH bifurcation (region VI in Fig 4). Along either route, the stable fixed point corresponding to rest does not disappear, so recruitment is not guaranteed. Note that recruitment could still occur through mechanism A. In contrast, if node 2 begins in active rest, it may enter the LCs region via the ictal-only region or via the region between the SN bifurcations (region II in Fig 4). In either case, the stable fixed point corresponding to active rest becomes unstable and a stable limit cycle appears (Fig 4B, bottom two panels). The location of the unstable fixed point is within the basin of attraction of the limit cycle, enabling recruitment through mechanism B. S1 Fig illustrates similar state space arguments for higher recruitment proportions when node 1 is in LCb and node 2 is in active rest compared to rest.

**Fig 4:**
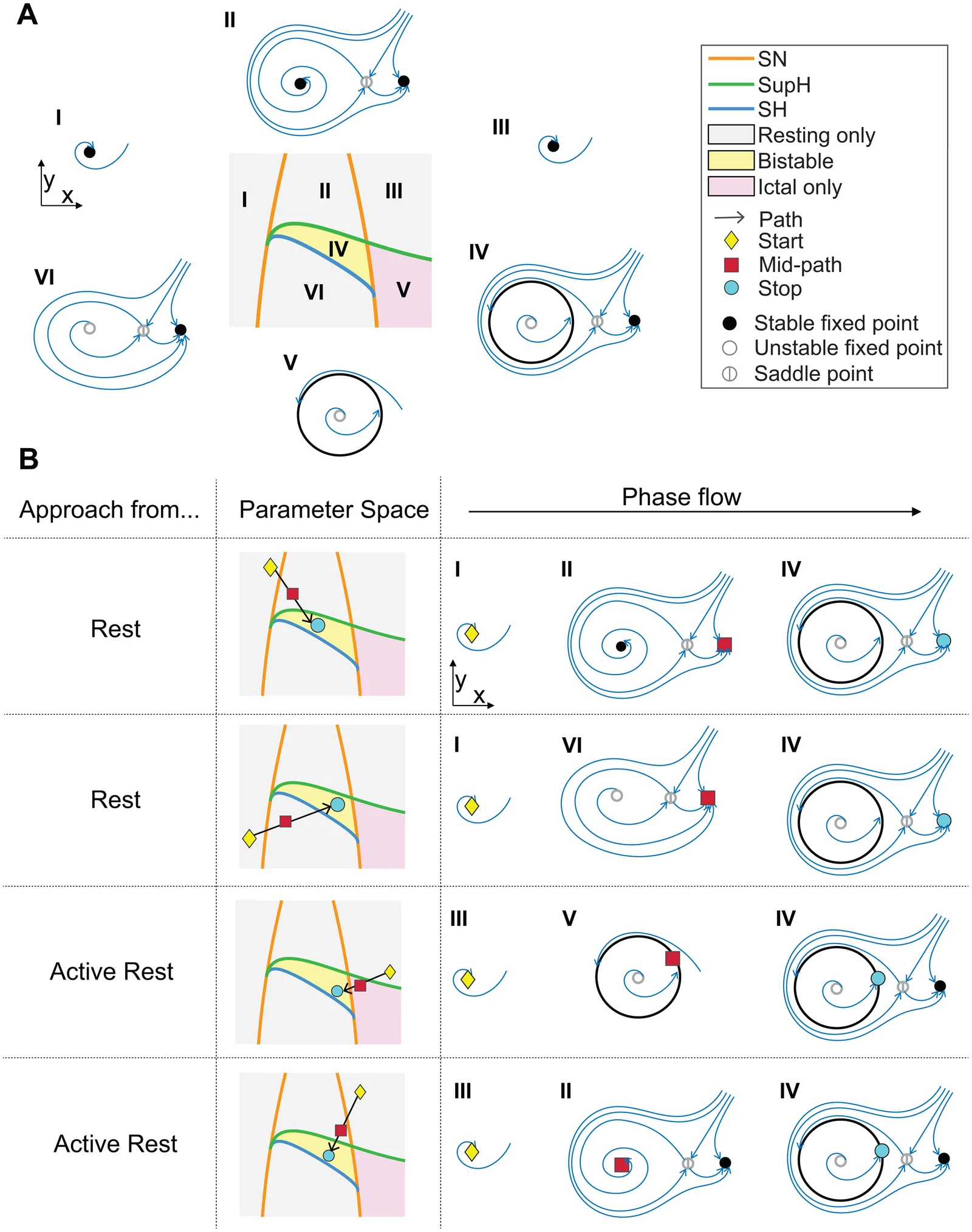
State space analysis during recruitment to LCs. (A) Flow diagrams in each region surrounding LCs, and in LCs. (B) Phase flow diagrams demonstrating changes in state space during recruitment to LCs from different directions. Blue lines are flow lines. During recruitment from rest (top two panels), the system begins on a stable fixed point that remains stable along the path to and in the LCs region. During recruitment from active rest (bottom two panels), the system begins on a stable fixed point that becomes unstable along the path to or upon entering the LCs region. LCs = limit cycle small, SN = saddle node, supH = supercritical Hopf, SH = saddle homoclinic.

The third theoretical prediction is that the onset dynamics of node 2 may be the same as or different from those of node 1. Mechanism A ensures node 1 and node 2 encounter the same onset bifurcation, encouraging similar onset behavior. If node 1 exhibits a baseline shift, for example, node 2 will also exhibit a baseline shift. However, mechanism B allows for the dynamics of node 2 to diverge from those of node 1. When node 2 is recruited through mechanism B, it can exhibit supH or subH onset dynamics. Depending on node 1’s onset bifurcation, the dynamics of node 1 and node 2 may differ. Note that this prediction is only relevant with our choice of coupling mechanism, in which node 2 can initially be in a different region of parameter space than node 1.

### Dynamical factors that influence seizure recruitment properties *in silico*

Next, we simulated a wide range of dynamics in both the seizure onset zone and propagation zone. We investigated recruitment properties via measurement of recruitment proportion, recruitment delay, and recruitment energy. We considered two dynamical factors that could influence recruitment properties: the onset dynamics of node 1 and the initial parameter space location of node 2. We also considered the effects of coupling strength (*G*) and node 1 excitability (d*).

#### Recruitment properties with respect to node 1 onset bifurcation.

Node 1 dynamotypes were divided into four groups by onset bifurcation: SN(+DC), supH, SN(-DC), and subH. SN(+DC) onsets are characterized by baseline shifts. SupH onsets are characterized by increasing amplitude from zero. SN(-DC)- and subH-onset dynamotypes are both characterized by arbitrary amplitude without baseline shift [16]. Results with respect to node 1 onset bifurcation are summarized in Table 2. We found these results to be robust to the influence of parameter space geometry and coupling term. We also evaluated SN(+DC) followed by supH-onset dynamotypes, which showed recruitment properties between those of SN(+DC)- and supH-onset dynamotypes in isolation (S2 File).

**Table 2:** Summary of recruitment properties by onset bifurcation.

Onset Bifurcation	Relative Recruitment Properties			Impact on Recruitment Likelihood	
	Likelihood	Speed	Energy	Coupling Strength *G*	Node 1 Excitability d^*^
SN (+DC)	Medium-Low	Slow	High	Medium	Medium
SupH	Low	Variable	Low	None	None
SN(-DC)	Medium-High	Fast	Medium-High	High	Medium
SubH	High	Fast	Medium-Low	High	Low

Consistent with theoretical predictions, nodes in the LCb bistability region were more likely to recruit than nodes in the LCs bistability region. SN(-DC)- and subH-onset dynamotypes, located in LCb, showed elevated recruitment (Fig 5A) compared to SN(+DC)- and supH-onset dynamotypes, located in LCs. Recruitment proportion was particularly high for subH-onset dynamotypes, and particularly low for supH-onset dynamotypes. All differences in recruitment proportion were statistically significant (Kruskal-Wallis test p = 2.5x10^-184^; post-hoc testing with Bonferroni correction yielded p < 0.001 for all pairwise comparisons). Thus, the model predicts that seizures that do not start with baseline shifts are more likely to spread than seizures that display baseline shifts at onset. Seizures with amplitude scaling at onset are unlikely to spread.

**Fig 5:**
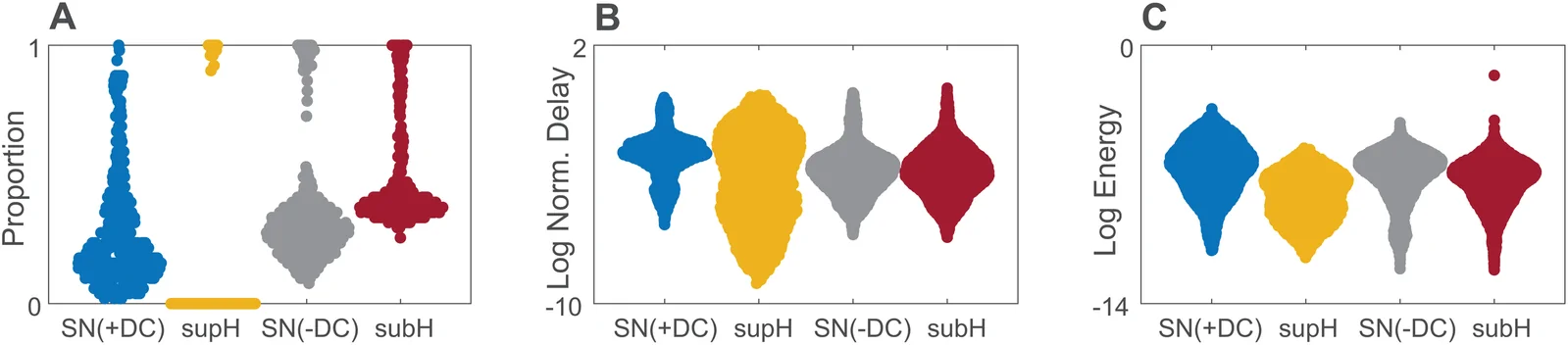
Recruitment properties depend on node 1 onset bifurcation. (A) Recruitment proportion. Each datapoint represents one node 2 location. (B) Log of normalized recruitment delay and **(C)** log of recruitment energy. Each datapoint represents one simulation. All pairwise comparisons in (A) and (C) were statistically significant. Statistical significance was evaluated via Kruskal-Wallis tests followed by multiple comparisons with Bonferroni correction at a significance level of 0.001. In (B), SN(+DC)-onset node 1 dynamotypes were associated with significantly higher recruitment delays than all other node 1 dynamotypes. Also, subH-onset node 1 dynamotypes had significantly higher delays than supH-onset node 1 dynamoytpes. SN(+DC) = saddle node with baseline shift, supH = supercritical Hopf, SN(-DC) = saddle node without baseline shift, subH = subcritical Hopf.

SN(+DC)-onset node 1 dynamotypes also demonstrated longer recruitment delays (Fig 5B) compared to all other dynamotypes (Kruskal-Wallis test p  < 2.2x10^-308^; p < 1x10^-190^ for all pairwise comparisons with Bonferroni correction including SN(+DC)-onset dynamotypes). Hence, the model predicts that seizures that exhibit baseline shifts at onset spread more slowly than seizures that do not exhibit baseline shifts at onset. Recruitment energy was highest for SN(+DC)-onset node 1 dynamotypes, followed by SN(-DC)-onset dynamotypes, then subH-onset dynamotypes, and finally lowest for supH-onset dynamotypes (Fig 5C). All differences were statistically significant (Kruskal-Wallis test p < 2.2x10^-308^; p < 1x10^-31^ for all pairwise comparisons with Bonferroni correction).

Recruitment proportions versus coupling strengths and node 1 excitability values are displayed in Fig 6. Across all simulations, the likelihood of recruitment increased with node 1 excitability and with the strength of the coupling between the nodes (Fig 6A). Recruitment patterns varied by onset bifurcation. SN(+DC)-onset dynamotypes showed a gradual increase in recruitment proportion with coupling strength and node 1 excitability (Fig 6B). In contrast, SN(-DC)- and subH-onset node 1 dynamotypes displayed an abrupt increase in recruitment proportion at a coupling strength of 0.02, with a smaller dependence on node 1 excitability (Fig 6C, 6E). The model therefore predicts that seizures that exhibit baseline shifts or arbitrary amplitude at onset are more likely to recruit when the onset zone is well-connected to potential propagation zones. The excitability of the onset zone has a greater influence on recruitment for seizures that start with baseline shifts compared to those that exhibit arbitrary amplitude at onset.

**Fig 6:**
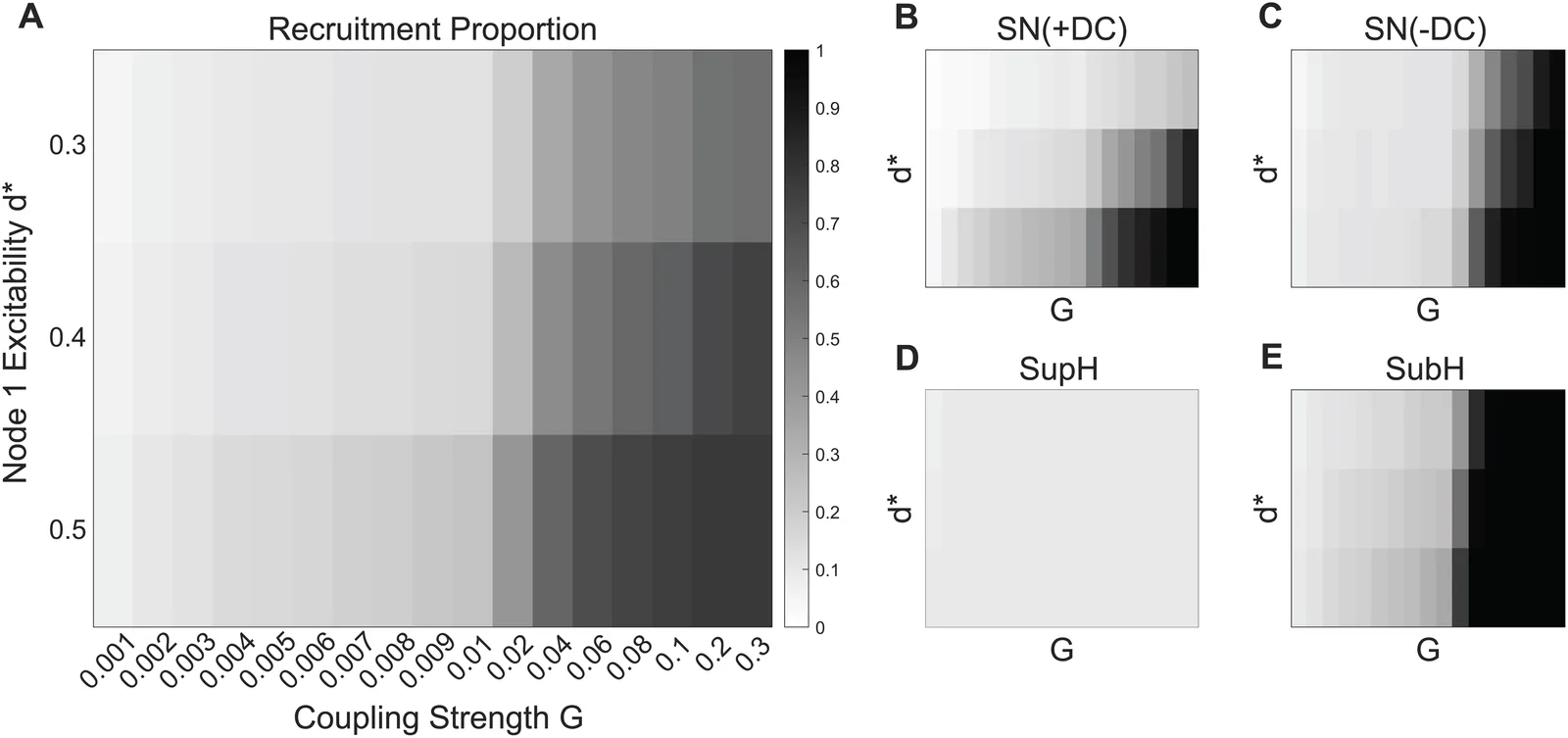
Recruitment proportion versus coupling strength (G) and node 1 excitability (d*). (A) Results for all simulations. Recruitment increased with coupling strength and node 1 excitability. (B)-(E) Results for each node 1 onset bifurcation. Dependence on coupling strength and node 1 excitability varied by node 1 onset bifurcation. SN(+DC) = saddle node with baseline shift, supH = supercritical Hopf, SN(-DC) = saddle node without baseline shift, subH = subcritical Hopf.

From theoretical analysis, we predicted that recruitment through mechanism A would be more sensitive to coupling strength and node 1 excitability compared to recruitment through mechanism B. It was no surprise then that supH-onset node 1 dynamotypes, which can only recruit through mechanism B, exhibited a consistent, low recruitment proportion across all coupling strengths and node 1 excitabilities (Fig 6D). The model predicts these factors do not influence recruitment for seizures that exhibit increasing amplitude at onset.

#### Recruitment properties with respect to node 2 initial location.

The initial location of node 2 in parameter space also significantly influenced recruitment properties (Kruskal-Wallis tests yielded p < 6.01x10^-7^ for comparisons of recruitment proportion, delay, and energy across node 2 initial locations). For most node 2 initial locations, recruitment proportion was consistently low (Fig 7). Recruitment proportions increased and more node 2 initial locations were recruited at high excitability and coupling strength values (S2 Fig). Generally, recruitment delays and energy were consistent and decreased moving from near the LCs region to near the LCb region (Fig 7). These results are consistent with recruitment via mechanism A. Recall that in mechanism A, node 2 reaches node 1 in parameter space before node 1 leaves its onset bifurcation. Thus, recruitment through mechanism A is likely to be low and increase with node 1 excitability and coupling strength. Further, recruitment delay and energy are stereotyped because node 2 can only be recruited in a narrow timespan.

**Fig 7:**
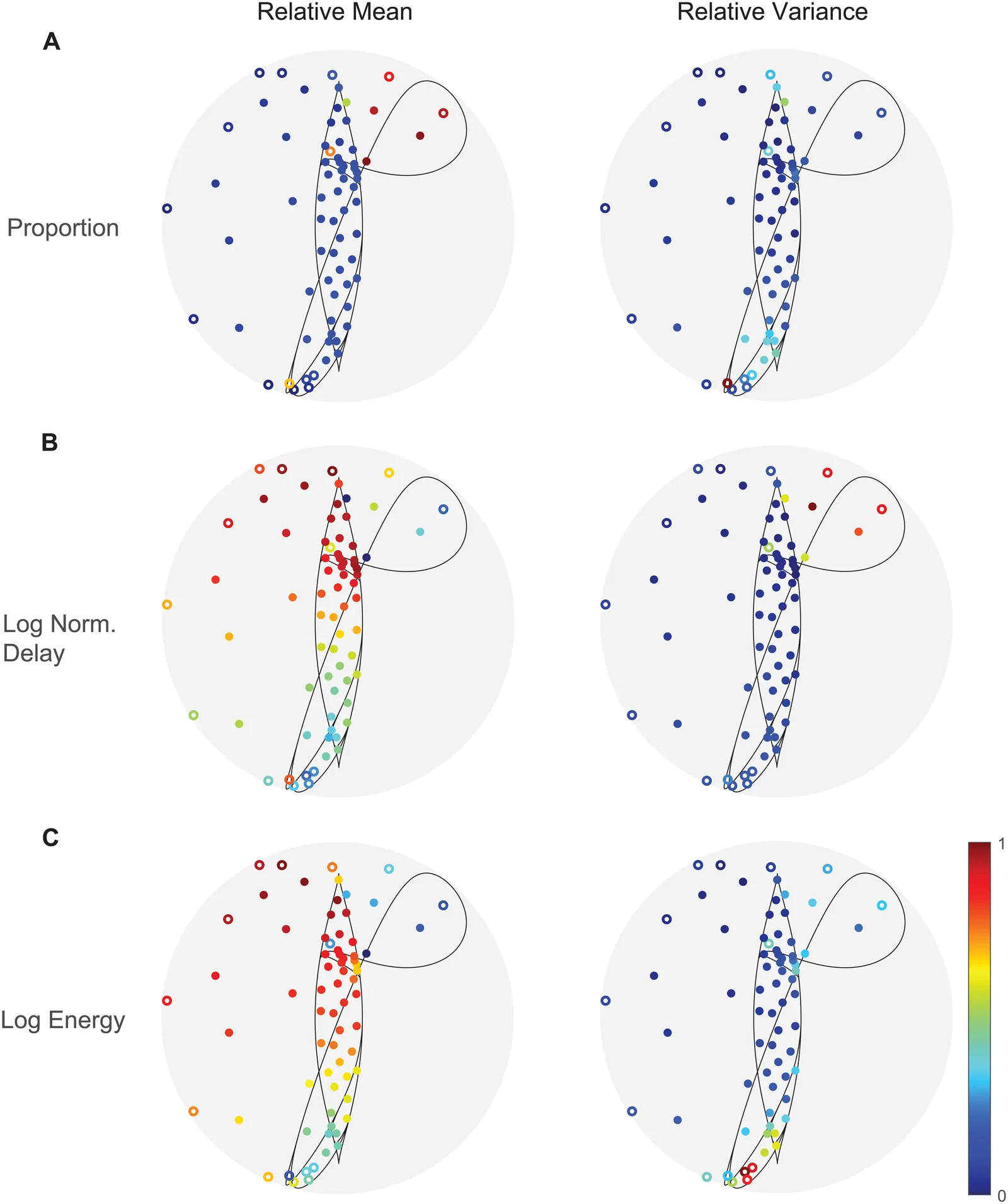
Node 2 initial location impacts recruitment properties. Relative mean and variance of **(A)** recruitment proportion, **(B)** log of normalized recruitment delay, and **(C)** log of recruitment. For most points, recruitment proportion was consistently low. Normalized recruitment delay and recruitment energy increased as the initial location of node 2 moved upwards on the sphere. A set of initial locations in the resting-only region in the top half of the sphere departed from these trends, instead showing more frequent recruitment, more variable recruitment delay, and lower recruitment energy. Initial location had a significant influence on all recruitment properties (Kruskal-Wallis tests yielded p < 6.01x10^-7^).

A set of initial locations in or near active rest – in the resting-only region, in the upper right quadrant of the sphere, very close to or to the right of the SN bifurcation – departed from the general trends. These initial locations demonstrated elevated recruitment proportions (Fig 7). They tended to be recruitable regardless of node 1’s onset bifurcation, even at low node 1 excitability and low coupling strength (S2 Fig). They were the only initial locations recruited by supH-onset node 1 dynamotypes (S2 Fig). These observations are consistent with the theoretical prediction that nodes in active rest can easily be recruited via mechanism B. Initial locations in or near active rest also showed lower and more variable recruitment delays, and reduced recruitment energy (Fig 7). The proximity of these nodes to the LCs and ictal-only regions likely lowered recruitment delays and energy. Recruitment through mechanism B can occur at any point in the node 1 burst, facilitating variation in recruitment delay.

### Propagation zone dynamics

Although not mandatory, clinicians expect that recruited tissue will most commonly adopt the same bursting dynamics as the onset zone. Likewise, the propagation model, by construction, facilitated the occurrence of similar dynamics, but also allowed for situations where that may not be true. Here, we illustrate the routes to diverse propagation zone dynamics via qualitative analysis of recruitment examples, with special attention to model predictions that are clinically unexpected. To demonstrate that unusual model predictions are indeed plausible, we compare to seizures recorded in human iEEG. Note that while only a few examples are included for brevity, the entire range of propagation zone dynamics predicted by the model and comparisons to human iEEG are provided in S2 File.

In a typical case of recruitment through mechanism A, node 2 displayed the same onset dynamics as node 1. Fig 8A shows such a typical example of recruitment for a SN(+DC)-onset node 1 dynamotype. After being recruited from rest, the propagation zone exhibited the same onset dynamics – namely, a baseline shift – as node 1. This recruitment behavior aligns with the general clinical expectation. The top two traces of the human seizure in Fig 9 provide an example analogous to the model’s prediction. This seizure began in the basal temporal area with a clear baseline shift at onset, compatible with a SN(+DC) bifurcation. The right lateral temporal propagation zone also demonstrated a change in baseline at onset. Also note that in this clinical seizure, each propagation zone demonstrated unique onset dynamics. In the context of coupled nodes of the Multiclass Epileptor, these differing onset patterns suggest the initial dynamical states of the propagation channels varied.

**Fig 8:**
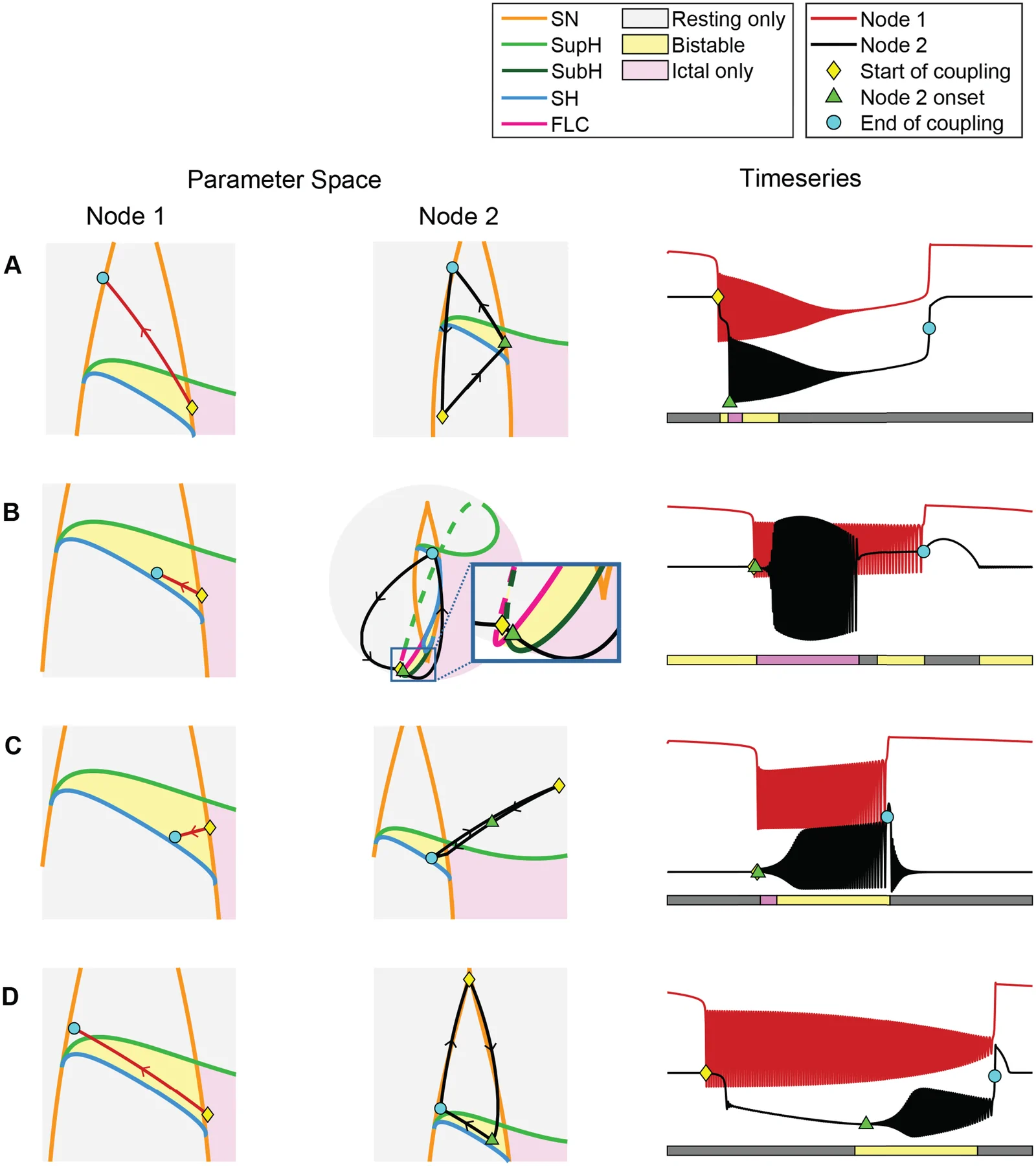
Propagation zone behavior for SN(+DC)-onset node 1 dynamotypes. Parameter space movement is shown on the left. Timeseries (offset for visualization) are shown on the right. The shaded bars under the timeseries indicate the parameter space region of node 2. Arrows indicate the direction of travel in parameter space. Yellow diamonds mark the location of node 1 and node 2 in parameter space at the time of coupling onset. Cyan circles mark the location of node 1 and node 2 in parameter space at the time of coupling offset. Green triangles mark the location of node 2 in parameter space at node 2 onset. The symbols are also shown in the node 2 timeseries (right). (A) Example in which the onset dynamics of node 2 matched that of node 1. (B)-(D) Examples in which the onset dynamics of node 2 differed from that of node 1. In (B), the box zooms into the LCb region for node 2. SN(+DC) = saddle node with baseline shift, SN = saddle node, supH = supercritical Hopf, subH = subcritical Hopf, SH = saddle homoclinic, FLC = fold limit cycle.

**Fig 9:**
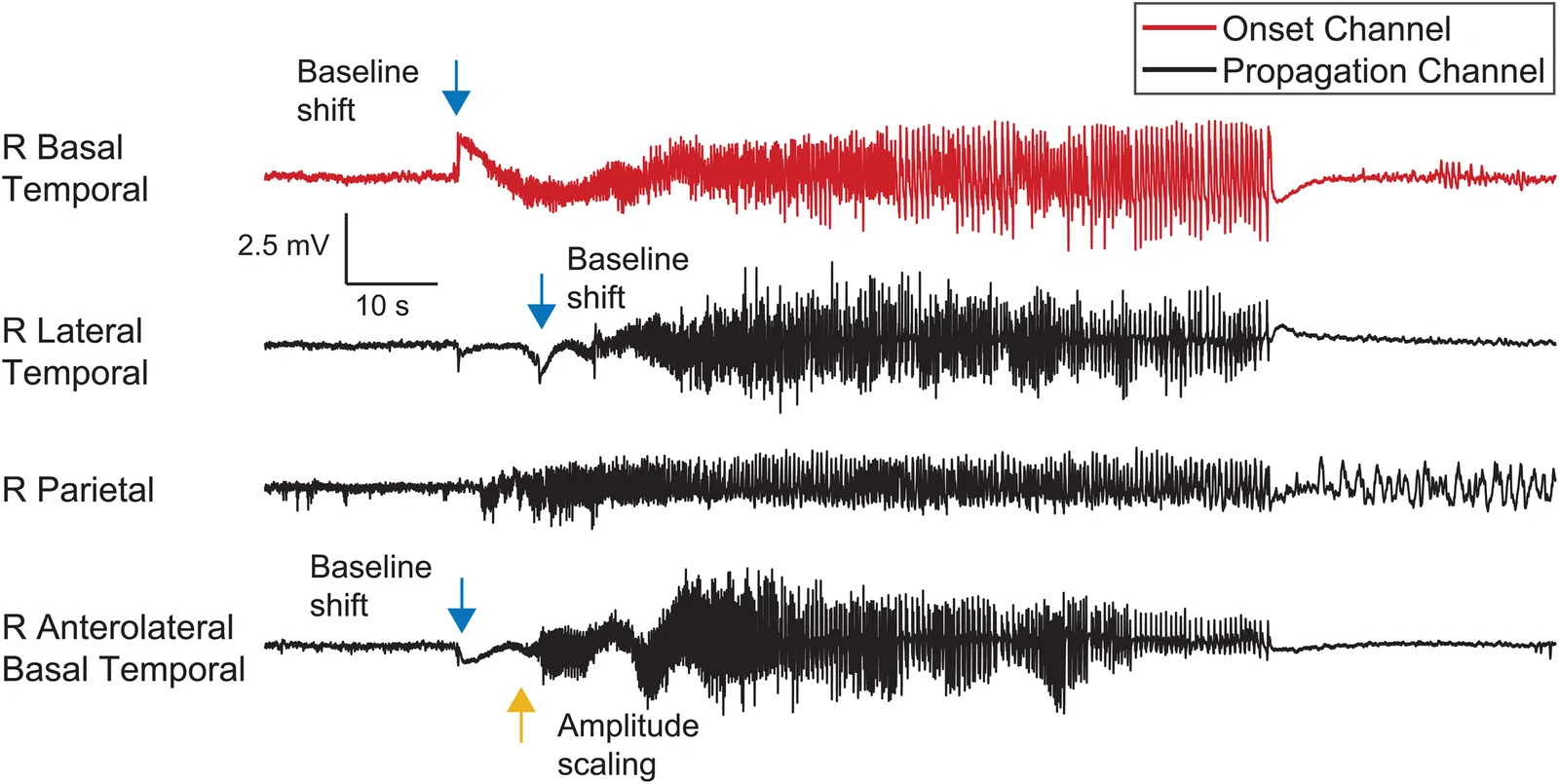
Clinical seizure propagation example. There is a baseline shift at onset in the onset channel, compatible with a SN(+DC) onset bifurcation. Propagation channel dynamics vary. The right lateral temporal channel shows a baseline shift at onset, consistent with a SN(+DC) bifurcation. The right parietal channel exhibits arbitrary amplitude without a change in baseline at onset, consistent with a SN(-DC) or subH bifurcation. The right anterolateral basal temporal channel displays a baseline shift followed by increasing amplitude at onset, consistent with a baseline drift followed by a supH bifurcation. R = right.

Recruitment through mechanism B could lead to a mismatch in the onset dynamics of node 1 and node 2. Fig 8B and C highlight two such examples. In Fig 8B, node 2 crossed a subH bifurcation to enter the ictal-only region on the path to node 1 and demonstrated arbitrary amplitude *without* a baseline shift at onset. In Fig 8C, node 2 began to burst via a supH bifurcation, thus exhibiting increasing amplitude at onset. Despite the unexpected nature of these model predictions, we found examples of both behaviors in clinical data. In the third trace in Fig 9, the right parietal propagation zone exhibited arbitrary amplitude at onset despite the baseline shift in the onset channel. S3 Fig provides an example of a possible SN(+DC) onset in the onset zone, followed by amplitude scaling at onset in the propagation zone.

Throughout the parameter space map, the position of the resting state (stable fixed point) varies. We found that node 2 could experience a drift in the location of the resting state while traversing parameter space, which appeared similar to a baseline shift in the timeseries. Note that we use the term *drift* rather than *shift* to distinguish these changes in baseline from the abrupt changes at burst onset caused by a SN bifurcation. In Fig 8D, node 2 crossed from rest to active rest and experienced a concurrent baseline drift prior to burst onset. Burst onset then occurred through a supH bifurcation and was associated with increasing amplitude. The bottom trace in Fig 9 highlights an analogous clinical example. Such drifts in baseline could also occur in the absence of a baseline shift in node 1, as exemplified in Fig 10A. This situation is particularly unexpected clinically, given that there is literature indicating baseline shifts may be a biomarker of the seizure onset zone [33–35]. Yet, we were able to find an example in human EEG. In Fig 10B, the seizure began in the left hippocampus with increasing amplitude and without a baseline shift at onset. After a delay, the seizure spread to propagation channels in the right hippocampus, where there was a change in baseline at onset.

**Fig 10:**
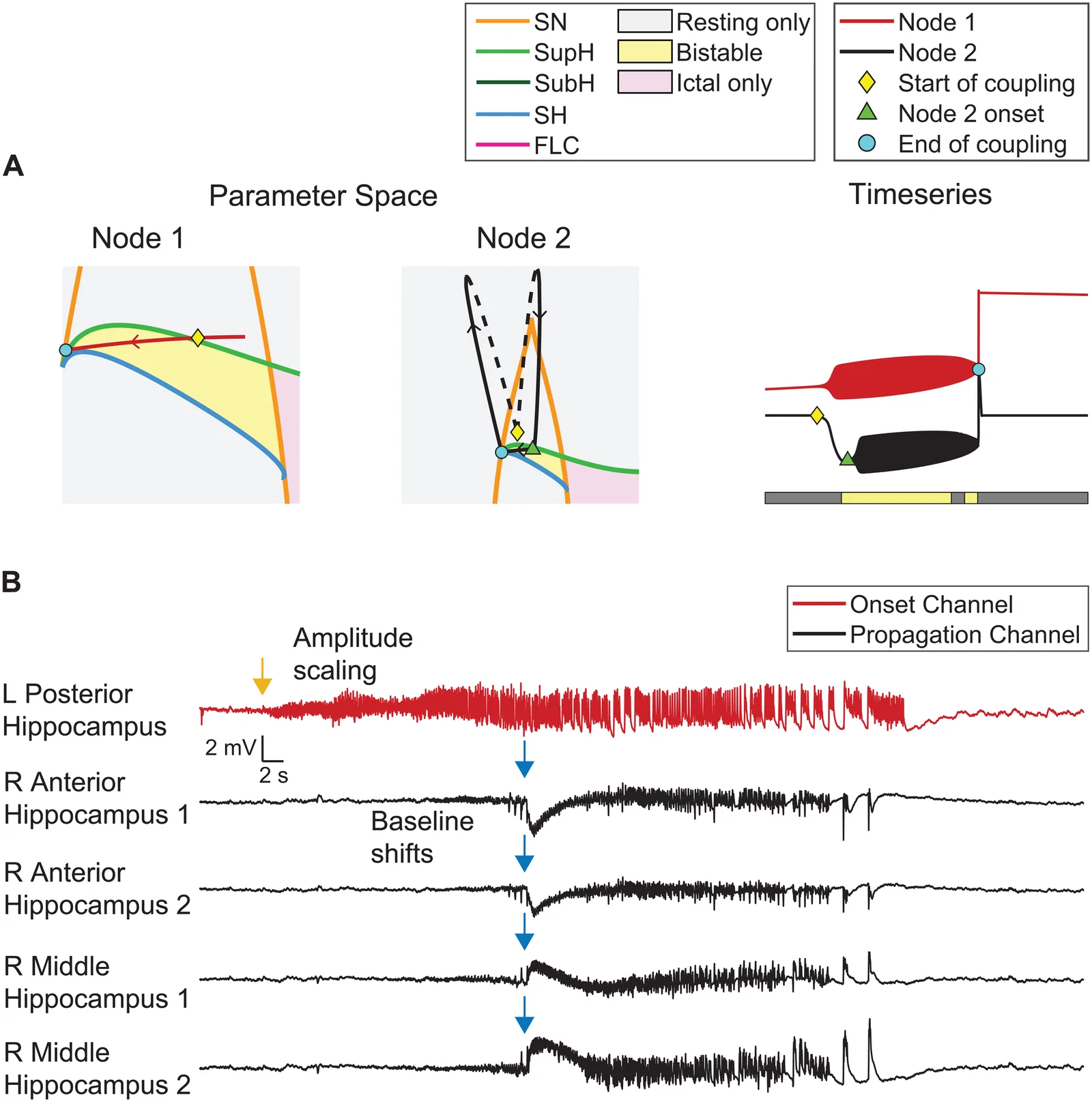
Changes in baseline can be isolated to the propagation zone. (A) Recruitment example in which node 1 is a supH-onset dynamotype, showing amplitude scaling at onset without a baseline shift. Node 2 demonstrates a baseline drift just before onset. Parameter space movement is shown on the left. Timeseries (offset for visualization) are shown on the right. The shaded bars under the timeseries indicate the parameter space region of node 2. Arrows indicate the direction of travel in parameter space. Yellow diamonds mark the location of node 1 and node 2 in parameter space at the time of coupling onset. Cyan circles mark the location of node 1 and node 2 in parameter space at the time of coupling offset. Green triangles mark the location of node 2 in parameter space at node 2 onset. The symbols are also shown in the node 2 timeseries (right). SN = saddle node, supH = supercritical Hopf, subH = subcritical Hopf, SH = saddle homoclinic, FLC = fold limit cycle. (B) Analogous clinical seizure propagation example. There is increasing amplitude at onset in the onset channel, compatible with a supH bifurcation. The propagation channels display clear changes in baseline at onset. Numbers indicate electrode contacts. L = left, R = right.

Breaking of timescale separation in some simulations with sufficiently high coupling strength also allowed the onset pattern of node 2 to vary from that of node 1. Fig 11A illustrates an example. Here, node 1 was a supH-onset dynamotype and demonstrated amplitude scaling at burst onset. During recruitment, node 2 quickly crossed into the LCs region via the supH bifurcation. Due to loss of timescale separation during movement across the supH bifurcation, there was no amplitude scaling at burst onset. The clinical seizure example in Fig 11B confirms the potential for this recruitment behavior in human epilepsy.

**Fig 11:**
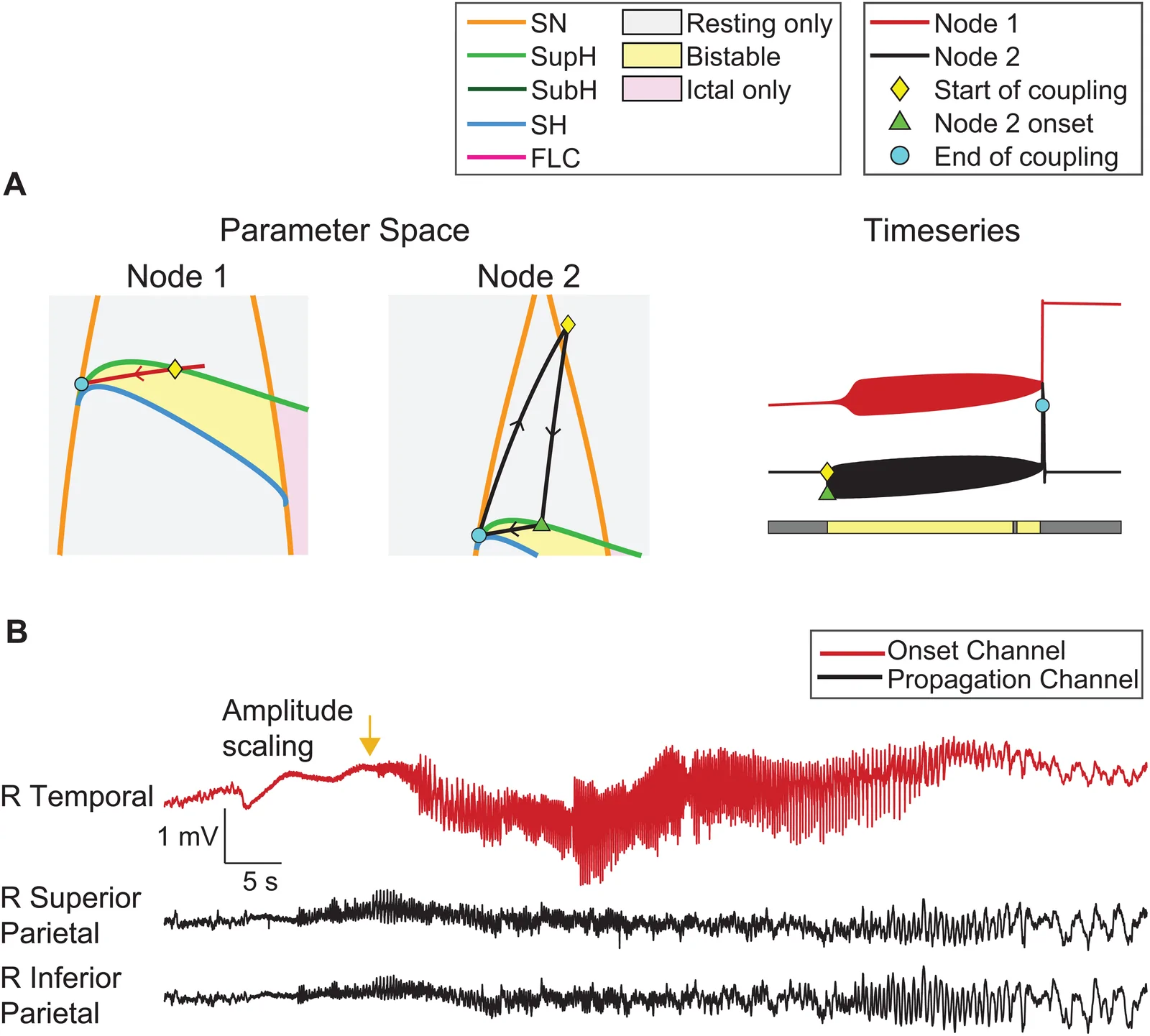
Amplitude scaling can be isolated to the seizure onset zone. (A) Recruitment example in which node 1 is a supH-onset dynamotype, showing amplitude scaling at onset. Node 2 demonstrates arbitrary amplitude onset. Parameter space movement is shown on the left. Timeseries (offset for visualization) are shown on the right. The shaded bars under the timeseries indicate the parameter space region of node 2. Arrows indicate the direction of travel in parameter space. Yellow diamonds mark the location of node 1 and node 2 in parameter space at the time of coupling onset. Cyan circles mark the location of node 1 and node 2 in parameter space at the time of coupling offset. Green triangles mark the location of node 2 in parameter space at node 2 onset. The symbols are also shown in the node 2 timeseries (right). SN = saddle node, supH = supercritical Hopf, subH = subcritical Hopf, SH = saddle homoclinic, FLC = fold limit cycle. (B) Analogous clinical seizure propagation example. There is increasing amplitude at onset in the onset channel, compatible with a supH bifurcation. The propagation channels display arbitrary amplitude at onset. R = right.

## Discussion

### Seizure propagation model

#### Rationale for model development.

Seizures exhibit a high degree of degeneracy: there are many etiologies and mechanisms of seizure generation [9,10]. The degenerate nature of seizures forces a choice between biological detail and universality. Computational (or indeed, experimental) models tied to biological mechanisms are unable to reveal generic properties generalizable across epilepsies. Conversely, phenomenological models lack a direct connection to biology. We chose the latter approach, sacrificing biological detail to focus on universal emergent properties of epileptic brains. Note that the phenomenological approach does not negate the possibility of relating components of the model to specific biophysical processes. Indeed, state variables in the Epileptor, a phenomenological model for one seizure dynamotype, have been related to specific ion concentrations and glutamatergic and GABAergic activity in a low magnesium hippocampal preparation [11]. However, the model’s predictions held even when inhibiting neurotransmitter release, demonstrating that they are tied to the phenomenon of seizures, not the specific mechanisms. Likewise, the propagation model presented here constrains the dynamics of seizure propagation, but not the underlying biological mechanisms, which may vary in different experimental conditions or patients.

To investigate how a rich array of seizure onset and propagation zone dynamics influence recruitment, we introduced a novel method to connect two nodes in the Multiclass Epileptor. To our knowledge, the Multiclass Epileptor is the only model in the literature that can reproduce a variety of dynamotypes (i.e., a range of onset and offset bifurcations) seen in human seizures in an easily controllable way. The Multiclass Epileptor describes the relationships between multiple bifurcations via the parameter space map; by establishing a coupling method in the Multiclass Epileptor, we enabled the first study of recruitment in this rich dynamical space. While different onset/offset bifurcations can also be obtained by tuning the parameters of well-known models such as the Jansen-Rit [36] and its extension, the Wendling-Chauvel [37], these models typically lack slow dynamics for autonomous seizure generation. Further, previous dynamical models of epilepsy networks have focused on single dynamotypes and generally require bistability or excitability [19–21,23,24,32,38,39]. In one recent study [40], different neural mass models were used in epileptogenic and non-epileptogenic zones, potentially enabling diverse dynamics. However, the onset bifurcation of the epileptogenic zone was not specified in that paper, and how different onset zone dynamics impact propagation was not studied. Thus, a unique aspect of our study is the simulation of a wide range of dynamics in the seizure onset and propagation zones.

There are multiple established methods for seizure generation in the Multiclass Epileptor [13,28]. For this work, we chose to model the seizure onset zone using the hysteresis-loop implementation, which is the dynamical mechanism exploited by the well-established Epileptor model [11,13,24,25,27,32,41]. The advantages of the hysteresis-loop implementation include that it produces autonomous seizures with generation and termination that rely on feedback from fast activity (see S1 File for expanded discussion). The strategy of fast-slow feedback is not unique to these phenomenological models: fast-to-slow feedback also tends to play a role in biophysically-inspired neural mass models for autonomous seizures [42–44]. The presence of hysteresis-loop slow dynamics in such high-dimensional models is a more complex question that still needs to be investigated. It is important to note that the primary results regard the pathway of node 2, which is not directly dependent on the implementation of node 1. However, using the hysteresis-loop implementation constrained the number of node 1 pathways and dynamotypes tested. In particular, node 1 did not enter the ictal-only region of parameter space. In the case in which node 1 paths move through the ictal-only region, we would expect node 2 to burst as soon as it is recruited into the ictal-only region (similar to the example in Fig 8B).

To connect two nodes of the model, we implemented a novel form of fast-to-slow coupling, allowing fast activity in node 1 (the seizure onset zone) to alter the dynamics of node 2 (the propagation zone). Fast-to-slow coupling has been used to connect brain regions with identical dynamics using the Epileptor in toy [24] and patient-specific [23,25,32] models of seizure propagation. Fast-to-slow coupling models the influence of fast variables (e.g., neural firing) in one brain region on slow variables (e.g., ion concentrations, ATP, tissue oxygenation) in another brain region. The perturbation of the slow variables may occur through any physiological process (e.g., synaptic transmission, ephaptic connections, gap junctions, or a combination therein), directly or indirectly. For example, a change in the homeostatic variables, like extracellular potassium concentration, in node 1 can indirectly alter those in node 2. Compared to fast-to-fast coupling, fast-to-slow coupling may better account for long recruitment times (up to seconds) during seizure propagation [24,25]. Further, fast-to-slow coupling has been shown to better capture patients’ seizure networks [25] than fast-to-fast coupling, although the timescale of coupling was not the most important factor in that study. Here, we extend fast-to-slow coupling to connect brain regions with diverse dynamics within the Multiclass Epileptor. While models implementing bifurcations as onset mechanisms have primarily used fast-to-slow coupling [23–25,32], fast-to-fast coupling is standard in models relying on noise to initiate a seizure [19–22,38,39]. The different coupling methods in the literature are not mutually exclusive. Indeed, a combination of coupling on different timescales may best predict the spatiotemporal diversity of propagation patterns in epilepsy [31]. Future work will investigate the interaction of coupling on different timescales in the Multiclass Epileptor.

The novelty of our implementation is that, during coupling, the dynamics (parameter values) of the potential propagation zone are altered towards that of the onset zone. For this first exploration of recruitment among nodes with diverse dynamics, we moved node 2 along an arc path to node 1. This is the simplest method and assumes that node 2 moves in the shortest path toward node 1. However, it is possible that node 2 would take other pathways, which would not necessarily follow the same sequences of bifurcation crossings nor necessarily follow an arc path. However, even in the context of more wandering paths, many of our results are still applicable. The two mechanisms of recruitment are independent of the path node 2 travels to reach node 1. Theoretical predictions regarding the likelihood of recruitment rely on the relative arrangement of regions in parameter space, in particular on the immediate neighboring regions of a given node 1’s onset bifurcation curve, and are expected to be robust to path. More complex paths may enable more complicated propagation zone behavior by crossing additional bifurcations. We already found that node 2 onset behavior could be the same as or differ from that of node 1; more complex paths would increase the potential for variations in onset behavior. Conversely, quantitative results, particularly those regarding recruitment speed and energy, may be altered in consideration of alternative node 2 paths. We leave the study of more complicated routes, and the behaviors and numerical results that may arise, to future work.

As a first approximation, we chose the speed of movement of node 2 towards node 1 to be proportional to the square of the difference from rest in node 1’s *x* variable (Eqn. 3). In developing this coupling function, we reasoned that the novel activity in the seizure onset zone (i.e., the electric field generated, represented by the change in voltage: *x*_*1*_ *– x*_*1, rest*_) influences the propagation zone. The coupling is phenomenological and thus effectively summarizes the cumulative effect of a variety of processes which depend on the specific conditions and locations of the brain regions involved. We squared the change in voltage to avoid biasing recruitment towards bursting paths in LCs (which have a distinct separation between bursting and resting states; see [13]) compared to bursting paths in LCb (in which oscillations cross the resting state). To further ensure that the coupling function was not biasing results, we calculated the area under the entire coupling term G(*x*_*1*_ *– x*_*1, rest*_)^2^ during the period between node 1 and node 2 onset. We called this value the recruitment energy. If the coupling function biased recruitment towards certain dynamotypes, we would expect dynamotypes with elevated recruitment proportions to also demonstrate elevated recruitment energy. However, there was no consistent relationship between recruitment proportion and recruitment energy among node 1 onset bifurcations (Fig 5). We therefore infer that the differences in recruitment proportion are not due to biases or saturation effects introduced by the choice of coupling term. We also found no major difference in results with a different coupling function (S2 File). Nevertheless, other choices for the coupling term could be equally relevant and are an area for future study. Other coupling terms may change the speed of movement of node 2 toward node 1, which could influence the likelihood of recruitment and recruitment time.

#### Model limitations.

A key aspect of the Multiclass Epileptor is timescale separation between the fast and slow subsystems. Timescale separation follows from the progression toward seizure termination, over the course of seconds or minutes, being much slower than the oscillations during the ictal phase. With fast-to-slow coupling, timescale separation holds only for small values of coupling strength. However, we chose to investigate a wider range of coupling strengths, meaning that timescale separation did not hold in all examples of our propagation model. Specifically, timescale separation is temporarily broken for node 2 between node 1 onset and when node 2 reaches node 1, when coupling strength is sufficiently high. During the period of broken timescale separation, the topology of the parameter space map and invariant properties of bifurcations may not hold. As such, node 2’s EEG (*x* variable) may show unexpected behavior. Most obviously, node 2 may cross a bifurcation without demonstrating that bifurcations’ properties (scaling laws, presence/absence of baseline shifts). This further complicates identification of onset bifurcation in clinical data. Note that timescale separation is only broken for node 2, and only during the initial recruitment period. There is physiological rational for this situation. When a clinical seizure begins in the seizure onset zone, the propagation zone may be recruited quickly – often within milliseconds [45]. That recruitment can span the entirety of the brain, and it is unlikely every brain node is always at the same dynamical baseline. That the seizure onset zone can quickly recruit propagation zones from a range of initial dynamical conditions (e.g., Fig 9; evidenced by different onset behavior in each propagation zone) suggests that temporary breakage of timescale separation is plausible. Further, violation of timescale separation was required to produce certain recruitment behaviors. For example, node 2 could cross a supH bifurcation but lack amplitude scaling (as in Fig 11A) if the movement on the map was sufficiently fast, enabling arbitrary amplitude in node 2 when recruited by a supH-onset node 1. The propagation pattern in the human seizure in Fig 11B is consistent with this recruitment behavior.

To focus on the dynamical conditions under which a seizure onset zone is more likely to recruit a propagation zone, we implemented a toy model of seizure propagation. While the use of such a toy model facilitated the study of dynamical factors and recruitment in isolation, it is a simplification of clinical seizure propagation. In our toy model, coupling was one-directional from a single, epileptogenic seizure onset zone to a single, non-epileptogenic propagation zone. We did not consider complex feedback that can occur in larger network models or with reciprocal coupling, thereby negating suppression effects. Further, biological (e.g., anatomy, structural connectivity) and dynamical factors will interact to produce a patient’s specific propagation patterns. For example, we found that, under sufficiently high coupling strength, SN(+DC)-onset dynamotypes can demonstrate recruitment proportions on par with SN(-DC)- and subH-onset dynamotypes (Fig 6). High coupling strength could correspond to increased structural or functional connectivity, or other physiological processes that increase the relative influence of the seizure onset zone on a brain region. Our use of a toy model is not meant to imply patient-specific anatomy and physiology is unimportant, but rather to motivate the inclusion of dynamical conditions in addition to these biological ones in future studies.

We also did not include noise in our simulations. In this work, we assume that seizure onset is precipitated by a bifurcation, and we consider the influence of bifurcation type on recruitment. For this reason, all simulations are performed without noise. This is in line with previous studies using the Epileptor [23,24,32], in which only low levels of additive noise are used to improve the realism of the simulations without altering the key dynamics. However, the inherently noisy nature of the brain and the fact that stronger or more complex noise can alter the dynamics of the model justifies future efforts to characterize the impact of noise. A first consequence of including noise at the single-node level (see [28] for a method), is that noise-induced transitions become possible in the presence of bistability [16]. The coupled nodes could therefore demonstrate either bifurcation- or noise-induced transitions within the bistable regions of parameter space. Other expected effects of noise in this model include excitable and chaotic dynamics. While evaluating the impact of noise on propagation is certainly a subject for future work, here we first establish the influence of dynamotype alone.

To ensure that our propagation model was plausible despite these limitations, we compared to EEG recordings in patients with epilepsy to ensure recruitment behaviors predicted by the model were possible clinically. One particularly unexpected prediction, from the clinical perspective, was the possibility to observe a change in baseline at onset in the propagation zone but not the seizure onset zone (Fig 10A). In focal epilepsy, a DC shift in the propagation zone only is a surprise – baseline shifts have been proposed as a biomarker of the seizure onset zone [33–35]. Yet, we found examples in intracranial EEG from several patients that confirmed this and other predictions (Fig 10B; see also Figs 9, 11, S3, S2 File). These examples are not meant to be a robust validation of the predictions; rather, they are a demonstration that the recruitment behaviors predicted using our propagation model, several of which were unusual and unexpected, were plausible and could be found even in our dataset of under 200 patients. Robust validation of all model predictions would require labeling exact onset times and compatible bifurcations in every channel (up to hundreds of channels) for tens to hundreds of seizures for every dynamotype in large human EEG datasets, a task far beyond the scope of this initial study. Our results motivate that future work, as well as the development of automated multi-channel dynamotype classification methods to enable such an undertaking.

### Factors impacting recruitment

We evaluated the influence of onset zone excitability, coupling strength, onset zone dynamotype, and propagation zone dynamics on recruitment likelihood, delay, and energy. The model predicted that recruitment properties depend on seizure onset zone dynamotype. Specifically, seizures that exhibited arbitrary amplitude without a baseline shift at onset recruited more often and faster than seizures that exhibited a baseline shift at onset. Seizures that displayed increasing amplitude from zero at onset were unlikely to propagate. While quantitative validation of model predictions is beyond the scope of this study, it is worth noting that generalized seizures – which propagate quickly and extensively – tend to exhibit arbitrary amplitude without a baseline shift at onset. In contrast, baseline shifts at seizure onset are a strong indicator of an isolated focal epilepsy [33–35], in which seizures do not always propagate quickly or extensively. Finally, the initial dynamics of the potential propagation zone influenced its likelihood of recruitment. In other words, some resting states were predicted to be more vulnerable to recruitment than others. These predictions provide a guide for future experiments to improve the methods to quantify and control human seizure spread.

### Clinical relevance

#### Motivation for identifying seizure dynamotypes.

Clinical seizure classification is primarily based on semiology and visual analysis of EEG [3,9]. Seizures are first divided into generalized, focal, and unknown onset, and may be subdivided by clinical symptoms and known or suspected etiology [46]. While these are useful descriptive tools, they ignore the underlying dynamics that define the seizure itself. The dynamotype is a complementary method of seizure classification based upon invariant seizure properties. Dynamical systems theory predicts that different onset bifurcations have distinct properties, which have recently been described in human epilepsy [16]. Previous work predicted that different dynamotypes are associated with differential response to aborting stimulation [27]. Here, we present the first demonstration of potential differences in seizure propagation among dynamotypes, further underscoring the utility in identifying patients’ dynamotypes. Knowledge that certain dynamotypes are more likely to recruit propagation zones may be useful for understanding a patient’s seizures, planning epilepsy surgery, and evaluating neuromodulation targets. The dynamotype classification also provides a language and basis for future studies investigating seizure propagation. That is not to exclude the relevance of other seizure dynamics – onset and offset bifurcation are but two possibly relevant dynamical features. The relevance of different dynamical features to seizures depends on the specific scientific or clinical question that is asked. Here, we chose to focus on onset bifurcation since this is highly relevant to recruitment.

#### Explanation of unusual propagation zone dynamics.

There is very little prior literature analyzing how dynamics change during propagation in human seizures. The clinical expectation is that recruited nodes will adopt the same dynamics as the seizure onset zone. When recruited brain regions have different dynamics, this situation is often assumed to mean that the second node is an independent seizure focus. However, our results suggest that a recruited node can adopt distinct dynamics simply due to its resting state position. This prediction provides a novel perspective on seemingly independent propagation zone dynamics.

#### Toward improved analysis of seizure networks.

A contributing factor to poor outcomes in resective surgery for drug-resistant focal epilepsy is a lack of understanding of patients’ seizure networks. A novel method used to better delineate onset and propagation zones is the use of the virtual epileptic patient (VEP), which seeks to recreate patients’ seizure networks in a computational model based on electrographic and imaging data [23,32]. Thus far, nodes in VEPs have been populated with Epileptors, which model the SN(+DC)/SH dynamotype. VEPs are created within the virtual brain framework [30], a platform for large-scale brain network models with realistic connectivity. Neural mass models other than the Epileptor can be implemented in the virtual brain framework [30]. Methods for multiscale simulation – i.e., combining phenomenological neural mass models with biophysical neuron models – in the virtual brain are an area of active development [47]. Patient-specific whole-brain seizure network models like the VEP seek to estimate the seizure onset zone and predict the outcomes of resective surgery, with an ultimate goal of providing a presurgical planning tool.

In demonstrating that seizure recruitment properties depend upon dynamotype, we motivate the inclusion of a range of dynamotypes in future whole-brain network dynamical models of epilepsy. Seizure dynamotypes are known to vary between patients and over time [16,18]. One seizure can also involve diverse dynamics in different areas of the brain (e.g., Fig 9). Thus, the inclusion of a wider range of dynamics in large scale dynamical models may provide more accurate and patient-specific results. There are several options for implementing the inclusion of richer dynamical detail in seizure network models. A recent analysis revealed that minor modifications of the Epileptor model can enable some additional bifurcations, albeit not all the bifurcations present in the Multiclass Epileptor [48]. The Epileptor is already integrated into the VEP workflow, so minor modifications may facilitate an increase in dynamical diversity without requiring major updates to the remainder of the pipeline. Another option is to populate each node in a seizure network model with a Multiclass Epileptor, which would capture a greater range of dynamotypes possible in epileptic brain. The cost of this greater dynamical detail would be more computationally intensive model inversion, given that the Multiclass Epileptor involves a larger parameter space. It is worth noting that despite increased computational complexity, current model inversion techniques can be successful even in high dimensional parameter spaces [49]. To reduce computational requirements, it may be helpful to identify the nearby bifurcations of each node in the virtual brain to partially constrain the choice of bursting path. This can be done through analysis of ictal timeseries [16] or in theory through perturbation analysis [17].

## Supporting information

S1 File Methods. (DOCX)

S2 File Results. (DOCX)

S3 File Video. Example of recruitment.
(MP4)

S4 File Zip file with Matlab code for the reviewers to perform the statistical analyses added in the revisions. This is a temporary file that will NOT BE PART of the final publication, because the final code will instead be posted to the Deep Blue Archive as listed in the Data Review URL. That file and this legend should be removed prior to publication.
(ZIP)

S1 Fig State space analysis during recruitment to LCb. (A) Flow diagrams in each region surrounding LCb, and in LCb. (B) Phase flow diagrams demonstrating changes in state space during recruitment to LCb from different directions. Blue lines are flow lines. During recruitment from rest (top two panels), the system begins on a stable fixed point that remains stable along the path to and in the LCb region. During recruitment from active rest (bottom panel), the system begins on a stable fixed point that becomes unstable along the path to the LCb region. LCb = limit cycle big, SN = saddle node, supH = supercritical Hopf, subH = subcritical Hopf, SH = saddle homoclinic, FLC = fold limit cycle.
(EPS)

S2 Fig Recruitment proportions for each node 2 initial location, grouped by node 1 onset bifurcation, coupling strength, and excitability values. Low excitability was d* = 0.3; high excitability was d* = 0.5. Low coupling strength was G < 0.01; high coupling strength was G > 0.05. Low and high coupling strengths were defined based on the observation that overall recruitment proportions increased around a coupling strength of 0.02. SN(+DC) = saddle node with baseline shift; supH = supercritical Hopf; SN(-DC) = saddle node without baseline shift; subH = subcritical Hopf.
(EPS)

S3 Fig Additional clinical seizure propagation example. There is a baseline shift at onset in the onset channels, compatible with a SN(+DC) onset bifurcation. In the propagation channel, increasing amplitude at onset is consistent with a supH onset bifurcation.
(EPS)

## References

[1] Fisher RS, Acevedo C, Arzimanoglou A, Bogacz A, Cross JH, Elger CE, et al. ILAE official report: a practical clinical definition of epilepsy. Epilepsia. 2014;55(4):475–82. doi: 10.1111/epi.12550 24730690

[2] Milligan TA. Epilepsy: A Clinical Overview. Am J Med. 2021;134(7):840–7. doi: 10.1016/j.amjmed.2021.01.038 33775643

[3] Thijs RD, Surges R, O’Brien TJ, Sander JW. Epilepsy in adults. Lancet. 2019;393(10172):689–701. doi: 10.1016/S0140-6736(18)32596-0 30686584

[4] Kwan P, Brodie MJ. Early identification of refractory epilepsy. N Engl J Med. 2000;342(5):314–9. doi: 10.1056/NEJM200002033420503 10660394

[5] Spencer S, Huh L. Outcomes of epilepsy surgery in adults and children. Lancet Neurol. 2008;7(6):525–37. doi: 10.1016/S1474-4422(08)70109-1 18485316

[6] Lüders HO, Najm I, Nair D, Widdess-Walsh P, Bingman W. The epileptogenic zone: general principles. Epileptic Disord. 2006;8 Suppl 2:S1-9. doi: 10.1684/j.1950-6945.2006.tb00204.x 17012067

[7] Bulacio JC, Chauvel P, McGonigal A. Stereoelectroencephalography: Interpretation. J Clin Neurophysiol Off Publ Am Electroencephalogr Soc. 2016;33:503–10. doi: 10.1097/WNP.0000000000000305 27918345

[8] Jobst BC, Bartolomei F, Diehl B, Frauscher B, Kahane P, Minotti L, et al. Intracranial EEG in the 21st Century. Epilepsy Curr. 2020;20:180–8. doi: 10.1177/1535759720934852 PMC7427159 32677484

[9] Scheffer IE, Berkovic S, Capovilla G, Connolly MB, French J, Guilhoto L, et al. ILAE classification of the epilepsies: Position paper of the ILAE Commission for Classification and Terminology. Epilepsia. 2017;58(4):512–21. doi: 10.1111/epi.13709 28276062 PMC5386840

[10] Stöber TM, Batulin D, Triesch J, Narayanan R, Jedlicka P. Degeneracy in epilepsy: multiple routes to hyperexcitable brain circuits and their repair. Commun Biol. 2023;6(1):479. doi: 10.1038/s42003-023-04823-0 37137938 PMC10156698

[11] Jirsa VK, Stacey WC, Quilichini PP, Ivanov AI, Bernard C. On the nature of seizure dynamics. Brain. 2014;137(Pt 8):2210–30. doi: 10.1093/brain/awu133 24919973 PMC4107736

[12] Lopes da Silva F, Blanes W, Kalitzin SN, Parra J, Suffczynski P, Velis DN. Epilepsies as dynamical diseases of brain systems: basic models of the transition between normal and epileptic activity. Epilepsia. 2003;44 Suppl 12:72–83. doi: 10.1111/j.0013-9580.2003.12005.x 14641563

[13] Saggio ML, Spiegler A, Bernard C, Jirsa VK. Fast-Slow Bursters in the Unfolding of a High Codimension Singularity and the Ultra-slow Transitions of Classes. J Math Neurosci. 2017;7(1):7. doi: 10.1186/s13408-017-0050-8 28744735 PMC5526832

[14] Baier G, Goodfellow M, Taylor PN, Wang Y, Garry DJ. The importance of modeling epileptic seizure dynamics as spatio-temporal patterns. Front Physiol. 2012;3:281. doi: 10.3389/fphys.2012.00281 22934035 PMC3429055

[15] Saggio ML, Jirsa VK. Phenomenological Mesoscopic Models for Seizure Activity. A Complex Systems Approach to Epilepsy. Cambridge University Press. 2022. p. 41–60. doi: 10.1017/9781108582285.004

[16] Saggio ML, Crisp D, Scott JM, Karoly P, Kuhlmann L, Nakatani M, et al. A taxonomy of seizure dynamotypes. Skinner FK, Frank MJ, Van Drongelen W, Valiante TA, editors. eLife. 2020;9:e55632. doi: 10.7554/eLife.55632 PMC7375810 32691734

[17] Izhikevich EM. Dynamical Systems in Neuroscience: The Geometry of Excitability and Bursting. MIT press; 2007.

[18] Crisp DN, Cheung W, Gliske SV, Lai A, Freestone DR, Grayden DB, et al. Quantifying epileptogenesis in rats with spontaneous and responsive brain state dynamics. Brain Commun. 2020;2(1):fcaa048. doi: 10.1093/braincomms/fcaa048 32671339 PMC7331126

[19] Goodfellow M, Rummel C, Abela E, Richardson MP, Schindler K, Terry JR. Estimation of brain network ictogenicity predicts outcome from epilepsy surgery. Sci Rep. 2016;6:29215. doi: 10.1038/srep29215 27384316 PMC4935897

[20] Sinha N, Dauwels J, Kaiser M, Cash SS, Brandon Westover M, Wang Y, et al. Predicting neurosurgical outcomes in focal epilepsy patients using computational modelling. Brain. 2017;140(2):319–32. doi: 10.1093/brain/aww299 28011454 PMC5278304

[21] Laiou P, Avramidis E, Lopes MA, Abela E, Müller M, Akman OE, et al. Quantification and Selection of Ictogenic Zones in Epilepsy Surgery. Front Neurol. 2019;10:1045. doi: 10.3389/fneur.2019.01045 31632339 PMC6779811

[22] Gerster M, Taher H, Škoch A, Hlinka J, Guye M, Bartolomei F, et al. Patient-Specific Network Connectivity Combined With a Next Generation Neural Mass Model to Test Clinical Hypothesis of Seizure Propagation. Front Syst Neurosci. 2021;15:675272. doi: 10.3389/fnsys.2021.675272 34539355 PMC8440880

[23] Wang HE, Woodman M, Triebkorn P, Lemarechal J-D, Jha J, Dollomaja B, et al. Delineating epileptogenic networks using brain imaging data and personalized modeling in drug-resistant epilepsy. Sci Transl Med. 2023;15(680):eabp8982. doi: 10.1126/scitranslmed.abp8982 36696482

[24] Proix T, Bartolomei F, Chauvel P, Bernard C, Jirsa VK. Permittivity coupling across brain regions determines seizure recruitment in partial epilepsy. J Neurosci. 2014;34(45):15009–21. doi: 10.1523/JNEUROSCI.1570-14.2014 25378166 PMC6608363

[25] Proix T, Bartolomei F, Guye M, Jirsa VK. Individual brain structure and modelling predict seizure propagation. Brain. 2017;140(3):641–54. doi: 10.1093/brain/awx004 28364550 PMC5837328

[26] Belykh I, Reimbayev R, Zhao K. Synergistic effect of repulsive inhibition in synchronization of excitatory networks. Phys Rev E Stat Nonlin Soft Matter Phys. 2015;91(6):062919. doi: 10.1103/PhysRevE.91.062919 26172784

[27] Szuromi MP, Jirsa VK, Stacey WC. Optimization of ictal aborting stimulation using the dynamotype taxonomy. J Comput Neurosci. 2023;51(4):445–62. doi: 10.1007/s10827-023-00859-7 37667137 PMC10754472

[28] Sheckler C, Kish K, Walker Z, Barkelew G, Crisp DN, Szuromi MP, et al. Dynamotypes for Dummies: A Toolbox, Atlas, and Tutorial for Simulating a Comprehensive Range of Realistic Synthetic Seizures. eNeuro. 2025;12(10). doi: 10.1523/ENEURO.0200-25.2025 41027733 PMC12549069

[29] Izhikevich EM. Neural Excitability, Spiking and Bursting. Int J Bifurc Chaos. 2000;10:1171–266. doi: 10.1142/S0218127400000840

[30] Sanz-Leon P, Knock SA, Spiegler A, Jirsa VK. Mathematical framework for large-scale brain network modeling in The Virtual Brain. Neuroimage. 2015;111:385–430. doi: 10.1016/j.neuroimage.2015.01.002 25592995

[31] Proix T, Jirsa VK, Bartolomei F, Guye M, Truccolo W. Predicting the spatiotemporal diversity of seizure propagation and termination in human focal epilepsy. Nat Commun. 2018;9(1):1088. doi: 10.1038/s41467-018-02973-y 29540685 PMC5852068

[32] Jirsa VK, Proix T, Perdikis D, Woodman MM, Wang H, Gonzalez-Martinez J, et al. The Virtual Epileptic Patient: Individualized whole-brain models of epilepsy spread. Neuroimage. 2017;145(Pt B):377–88. doi: 10.1016/j.neuroimage.2016.04.049 27477535

[33] Ikeda A, Taki W, Kunieda T, Terada K, Mikuni N, Nagamine T, et al. Focal ictal direct current shifts in human epilepsy as studied by subdural and scalp recording. Brain. 1999;122 (Pt 5):827–38. doi: 10.1093/brain/122.5.827 10355669

[34] Ikeda A, Takeyama H, Bernard C, Nakatani M, Shimotake A, Daifu M, et al. Active direct current (DC) shifts and “Red slow”: two new concepts for seizure mechanisms and identification of the epileptogenic zone. Neurosci Res. 2020;156:95–101. doi: 10.1016/j.neures.2020.01.014 32045575

[35] Kim W, Miller JW, Ojemann JG, Miller KJ. Ictal localization by invasive recording of infraslow activity with DC-coupled amplifiers. J Clin Neurophysiol. 2009;26(3):135–44. doi: 10.1097/WNP.0b013e3181a768d8 19424082

[36] Jansen BH, Rit VG. Electroencephalogram and visual evoked potential generation in a mathematical model of coupled cortical columns. Biol Cybern. 1995;73(4):357–66. doi: 10.1007/BF00199471 7578475

[37] Wendling F, Bartolomei F, Bellanger JJ, Chauvel P. Epileptic fast activity can be explained by a model of impaired GABAergic dendritic inhibition. Eur J Neurosci. 2002;15(9):1499–508. doi: 10.1046/j.1460-9568.2002.01985.x 12028360

[38] Benjamin O, Fitzgerald TH, Ashwin P, Tsaneva-Atanasova K, Chowdhury F, Richardson MP, et al. A phenomenological model of seizure initiation suggests network structure may explain seizure frequency in idiopathic generalised epilepsy. J Math Neurosci. 2012;2(1):1. doi: 10.1186/2190-8567-2-1 22657571 PMC3365870

[39] Hutchings F, Han CE, Keller SS, Weber B, Taylor PN, Kaiser M. Predicting Surgery Targets in Temporal Lobe Epilepsy through Structural Connectome Based Simulations. PLoS Comput Biol. 2015;11(12):e1004642. doi: 10.1371/journal.pcbi.1004642 26657566 PMC4675531

[40] Lopez-Sola E, Mercadal B, Lleal-Custey È, Salvador R, Sanchez-Todo R, Wendling F, et al. Personalized whole-brain models of seizure propagation. J Neural Eng. 2025;22(5). doi: 10.1088/1741-2552/ae08e9 40967238

[41] Zhou X, Wang Y, Si B. Controlling Epileptic Seizures through Hippocampal Regulation: A Complex Network Analysis in the Mouse Brain. IEEE Trans Neural Syst Rehabil Eng. 2025;33:4302–14. doi: 10.1109/TNSRE.2025.3616957 41042665

[42] Lopez-Sola E, Sanchez-Todo R, Lleal È, Köksal-Ersöz E, Yochum M, Makhalova J, et al. A personalizable autonomous neural mass model of epileptic seizures. J Neural Eng. 2022;19(5). doi: 10.1088/1741-2552/ac8ba8 35995031

[43] Gentiletti D, de Curtis M, Gnatkovsky V, Suffczynski P. Focal seizures are organized by feedback between neural activity and ion concentration changes. Elife. 2022;11:e68541. doi: 10.7554/eLife.68541 35916367 PMC9377802

[44] Rabuffo G, Bandyopadhyay A, Calabrese C, Gudibanda K, Depannemaecker D, Takarabe LM, et al. Biophysically inspired mean-field model of neuronal populations driven by ion exchange mechanisms. 2026. doi: 10.7554/elife.104249.2 PMC13626649 42814608

[45] Azeem A, Abdallah C, von Ellenrieder N, El Kosseifi C, Frauscher B, Gotman J. Explaining slow seizure propagation with white matter tractography. Brain. 2024;147(10):3458–70. doi: 10.1093/brain/awae192 38875488 PMC11449139

[46] Fisher RS, Cross JH, French JA, Higurashi N, Hirsch E, Jansen FE, et al. Operational classification of seizure types by the International League Against Epilepsy: Position Paper of the ILAE Commission for Classification and Terminology. Epilepsia. 2017;58(4):522–30. doi: 10.1111/epi.13670 28276060

[47] Hater T, Courson J, Lu H, Diaz-Pier S, Manos T. Arbor-TVB: a novel multi-scale co-simulation framework with a case study on neural-level seizure generation and whole-brain propagation. Front Comput Neurosci. 2026;19:1731161. doi: 10.3389/fncom.2025.1731161 41704907 PMC12907379

[48] Saggio ML, Jirsa V. Bifurcations and bursting in the Epileptor. PLoS Comput Biol. 2024;20(3):e1011903. doi: 10.1371/journal.pcbi.1011903 38446814 PMC10947678

[49] Vattikonda AN, Hashemi M, Woodman MM, Lemarechal J-D, Daini D, Bartolomei F, et al. High-resolution Bayesian Virtual Epileptic Patient using neural field models. Netw Neurosci. 2026;10(2):374–99. doi: 10.1162/NETN.a.543 42039098 PMC13108505

