# Supplementary material for: Seizure recruitment properties are dependent upon dynamotype: A modeling study
Source: PLoS Comput Biol. 2026 Sep 21;22(9):e1013975. doi: 10.1371/journal.pcbi.1013975 (PMC13626473; doi:10.1371/journal.pcbi.1013975)
Supplement: Supplementary file 1 [file pcbi.1013975.s001.docx]

**Supplementary methods**

**1. The Multiclass Epileptor**

A generic dynamical engine that reproduces a wide range of dynamotypes was previously created by exploiting the canonical structure around the degenerate Bogdanov-Takens singularity [1]. In the context of epilepsy, we refer to this DBT Burster as the Multiclass Epileptor to highlight that it is a more comprehensive version of the Epileptor, which describes one seizure dynamotype [2]. A full description of the DBT Burster has previously been published [1]; here, we provide a summary of the model.

From a dynamical perspective, epilepsy is characterized by at least two rhythms: a fast rhythm governs neural activity during a seizure, while a slower rhythm or process governs the transitions between seizure and resting states. Bursting thus characterized by periods of quiescence and activity is termed ‘fast-slow bursting’, and can be described by Eqns. 1-2 [1,3]. Eqn. 1 describes the fast subsystem given by state vector $\boldsymbol{x}=\boldsymbol{x}\left( t \right)\in\mathbb{R}^{n}$, and Eqn. 2 describes the slow subsystem given by state vector $\boldsymbol{z}=\boldsymbol{z}\left( t \right)\in\mathbb{R}^{m}$. Timescale separation between the subsystems is ensured by setting $k\ll1$.

$$\dot{\boldsymbol{x}}=f\left( \boldsymbol{x, z} \right) [1]$$

$$\dot{\boldsymbol{z}}=kg\left( \boldsymbol{x, z} \right) [2]$$

Reported in detail elsewhere [1,4,5], a minimally complex realization of the fast subsystem, comprised of two state variables such that $\boldsymbol{x}=(x,y)$, is given by Eqns. 3-4.

$$\dot{x}= -y \left[ 3 \right]$$

$$\dot{y}=x^{3}-\mu_{2}x-\mu_{1}-y\left( \nu+x+x^{2} \right) \left[ 4 \right]$$

The state variable $x$ is the output of the system, and tracks voltage content during a seizure, the equivalent of an EEG signal. Within the *(x, y)* state space of the model, resting states are represented by stable fixed points, and ictal states are represented by stable limit cycles. The existence of stable fixed points and limit cycles and therefore the behavior of the fast subsystem vary depending on the values of its parameters ${(\mu}_{1}, \mu_{2}, \nu)$. Because of the properties of this three-dimensional space, the relevant behaviors for this work can be captured by constraining the parameters of the fast subsystem to the surface of a sufficiently small sphere centered at the origin [5]. Note that the choice of parameter space geometry is arbitrary; only the relationships between parameter space regions with different dynamical repertoires are relevant, as captured, for example, using the spherical surface. Thus, as in previous work [1,4,6,7], we constrain the parameters of the fast subsystem to the surface of a sphere centered at the origin with radius 0.4 (Fig 1). This reduces the dimensionality of the parameter space to two (the angular coordinates theta and phi).

According to the location of the fast subsystem on this sphere, the system may be ‘resting’ (only a stable fixed point(s) exists), ‘ictal only’ (only a stable limit cycle exists), or ‘bistable’ with potential for both resting and ictal behavior (both a stable fixed point and a stable limit cycle exist). In the resting region, we distinguish ‘rest’ from ‘active rest’ based on the location of the fixed point in state space. The difference in the location of the fixed points is due to the geometry of the equilibrium manifold, as explained previously [1,7].

There are two ways to create a burst or seizure in the model [1,8], which link to the two major dynamical mechanisms proposed for seizure onset at the level of a single node [9–11]. Within the bistability regions, adding noise or an appropriate stimulus can cause the system to move between stable fixed point and stable limit cycle. The parameters of the fast subsystem need not change for such noise-induced transitions, although proximity to an onset bifurcation lowers the threshold for the transition. Alternatively, the transitions between resting and active states can be precipitated by slow changes in the fast subsystem’s parameters that alter the existence of stable fixed points and limit cycles; these types of transitions are known as bifurcations. On the surface of the parameter space sphere, bifurcations are represented by curves that divide regions of qualitatively distinct behavior repertoires (Fig 1). A seizure can be created when the fast subsystem traverses a path that crosses onset and offset bifurcations in parameter space. The slow subsystem of Eqn. 2 moves the fast subsystem along this bursting path.

Multiple realizations of the slow subsystem are possible [1,3,4]. In particular, hysteresis-loop implementations exploit bistability in the fast subsystem and feedback among fast and slow variables to produce autonomous bursting [12]. These paths capture the endogenous mechanisms that move the brain towards seizure termination upon onset, which occurs in all cases except status epilepticus [2]. The statistics of seizure termination support an underlying deterministic process [13]. In contrast, slow-wave implementations lack this feedback, may occur without bistability, and require slow variables to change independently to force the fast ones into bursting. The complexity of slow-wave implementations is also higher than that of hysteresis-loop implementations, requiring a minimum of two slow variables instead of one [1]. For the present work, we chose hysteresis-loop paths for the node representing the active seizure focus (node 1), enabling node 1 to start and end a seizure autonomously. This is the simplest choice that enables seizures that progress towards termination through feedback from the fast activity. Also, in the hysteresis-loop implementation, paths are realized as arcs between onset and offset bifurcations, which is a useful constraint for this initial work. A single implementation was chosen for comparison among bursting paths.

Eqn. 5 describes the speed and direction of movement of node 1 along its bursting path. In Eqn. 5, $D$ represents the Euclidean distance (in $\left( x, y \right)$state space) from rest and $d^{*}$ represents the excitability of the system. A larger excitability allows the fast system to move further from the resting state before the direction of motion in parameter space changes from toward onset bifurcation to toward offset bifurcation. Increasing $d^{*}$ alters the duty cycle of the slow subsystem such that the fast subsystem spends a greater proportion of time in the active (vs quiescent) state and near the onset bifurcation. The speed at which the fast subsystem moves along the bursting path is determined by $k_{1}$. To maintain timescale separation as in Eqn. 2, $k_{1}<<1$. Eqn. 5 prescribes that the system moves toward onset bifurcation when it is at rest and moves toward offset bifurcation when it is bursting.

$$\dot{z}=-k_{1}\left( D-d^{*} \right) [5]$$

A necessary condition for Eqn. 5 is that the bursting path exists in a bistable region of parameter space. We chose the bursting paths to be arcs of great circles that connect onset and offset bifurcations [1]. As mentioned above for the choice of the spherical surface, the validity of the model is only in terms of the relationships between parameter space regions (how they are placed with respect one to the other) and not in terms of their geometry (such as size and precise shape). Thus, paths that traverse the same sequence of regions are equivalent in this framework and we can choose the simplest one without loss of generality [1]. Details of the implementation method can be found in [1].

**2. Equations for node 1 and node 2**

In the propagation model, node 2 is initially placed at rest at a stationary point in parameter space, i.e. $\dot{z_{2}}=0$. Upon the onset of the burst in node 1, node 2 begins to move toward node 1 in parameter space along an arc connecting the nodes, with speed proportional to the square of the difference between the current state of node 1 and its resting state (in $x$). Since the x variable mimics a voltage recording, this form of coupling captures the influence of the change in voltage in the seizure onset zone (node 1) on the activity in the potential propagation zone (node 2). Following the offset of the burst in node 1, node 2 moves back to its initial location in parameter space at a constant speed ($k_{2}$). The parameterization of node 2’s path in parameter space, $\boldsymbol{\mu}\left( z \right)$, is given by Eqns. 6-8. In these equations, **A** is the location of node 2. During the coupling period, **B** is the location of node 1; after coupling ends, **B** is the initial location of node 2. **A** and **B** are updated at every timestep. R is the radius of the sphere (0.4).

$$\boldsymbol{\mu}\left( z \right)=R\left( \boldsymbol{E}\cos\left( z \right)+\boldsymbol{F}sin\left( z \right) \right)=\left( \begin{matrix} \mu_{2} \\ -\mu_{1} \\ \upsilon\end{matrix} \right) [6]$$

$$\boldsymbol{E}=\frac{\boldsymbol{A}}{R} [7]$$

$$\boldsymbol{F}=\frac{\left( \boldsymbol{A}x \boldsymbol{B} \right) x \boldsymbol{A}}{\left\| \left( \boldsymbol{A}x \boldsymbol{B} \right) x \boldsymbol{A} \right\|} [8]$$

The speed of node 2’s movement along this path, $\dot{z_{2}}$, is defined according to Eqn. 9. In Eqn. 9, $t_{on,1}$ is node 1 onset time, $t_{off,1}$ is node 1 offset time, $t_{rest,2}$is the time when node 2 reaches its initial location in parameter space following the burst in node 1, x_1_ is the value of the x variable for node 1, x_1, rest_ is the location of the fixed point for node 1, k_2_ is the speed of movement of node 2 back to its initial location, and $G$ is a coupling strength constant. x_1_ and x_1, rest_ are functions of time.

$$\dot{z}_{2}=\left\{ \begin{aligned} 0, t<t_{on,1} or t\geq t_{rest,2} \\ {G(x_{1}-x_{1, rest})}^{2}, t_{on,1}\leq t\leq t_{off,1} \\ k_{2}, t_{off,1}<t<t_{rest,2} \end{aligned} \right. [9]$$

The fast subsystem of node 2 is unchanged from Eqn. 3-4. With equations repeated for the reader, the system equations for node 1 and node 2 are given below. Subscripts indicate the node. For parameters $\mu_{1}$ and $\mu_{2}$, the first subscript indicates the parameter and the second the node (e.g. $\mu_{1,2}$ is $\mu_{1}$ for node 2). Recall that, in Eqn. 5, $D$ represents the Euclidean distance (in $\left( x_{1}, y_{1} \right)$state space) from rest and $d^{*}$ represents the excitability of the system. k_i_ in Eqns. 5, 9 determine the speed of movement of the relevant node in parameter space.

Node 1

$$\dot{x_{1}}= -y_{1} from \left[ 3 \right]$$

$$\dot{y_{1}}=x_{1}^{3}-\mu_{2,1}x_{1}-\mu_{1,1}-y_{1}\left( \nu_{1}+x_{1}+x_{1}^{2} \right) from \left[ 4 \right]$$

$$\dot{z}_{1}=-k_{1}\left( D-d^{*} \right) [5]$$

Node 2

$$\dot{x_{2}}= -y_{2} from \left[ 3 \right]$$

$$\dot{y_{2}}=x_{2}^{3}-\mu_{2,2}x_{2}-\mu_{1,2}-y_{2}\left( \nu_{2}+x_{2}+x_{2}^{2} \right) from \left[ 4 \right]$$

$$\dot{z}_{2}=\left\{ \begin{aligned} 0, t<t_{on,1} or t\geq t_{rest,2} \\ {G(x_{1}-x_{1, rest})}^{2}, t_{on,1}\leq t\leq t_{off,1} \\ k_{2}, t_{off,1}<t<t_{rest,2} \end{aligned} \right. [9]$$

**3. Simulation parameter details**

We simulated a wide range of node 1 classes, node 2 initial locations, coupling strength ($G$) values, and node 1 excitability ($d^{*}$) values to investigate the effects of these factors on recruitment properties. For each of the eight dynamotype classes that can be implemented via hysteresis-loop bursting, three paths were chosen arbitrarily. The speed of movement along each bursting path ($k_{1}$) was set slow enough that the characteristic properties of the dynamotype were evident in the timeseries. Initial conditions were adjusted such that the nodes were initially resting. $\boldsymbol{A, B,}$ $k_{1}$, and the initial conditions for each node 1 path are listed in Table A in S1 Methods. The variability of ictal inter-spike intervals across bursting paths was confirmed to be within the expected physiological range (within an order of magnitude; Fig A in S1 Methods).

For each dynamotype, onset and offset bifurcations, a representative parameter space bursting path, and the corresponding timeseries are displayed in Fig B in S1 Methods, panels C, D. Note that while we included all dynamotypes possible on the map in our analyses, offset bifurcation is less relevant to recruitment. Thus, our results are presented with respect to onset bifurcation only. As illustrated in Fig B in S1 Methods, panel B, 71 initial node 2 locations were simulated. Regions near bifurcations were sampled more heavily, while there was more sparse sampling in resting regions far from bifurcations. When initially located in a resting region, the system is in the quiescent state until a bifurcation is crossed, so there are only trivial differences in recruitment properties between two nearby points that are both far from bifurcations. Since node 2 was initially placed in the quiescent state (on a stable fixed point) to model a potential propagation zone, the ictal-only region of the sphere was avoided.

Seventeen values of coupling strength ($G$) were tested between 0.001 and 0.3. Three node 1 excitability ($d^{*}$) values were tested between 0.3 and 0.5. Together with the 24 bursting paths and 71 node 2 locations, there were a total of 86,904 simulations.

**Table A.** Node 1 offset $\boldsymbol{A}$, onset $\boldsymbol{B}$, speed $k_{1}$, and initial conditions $[x_{0},y_{0},z_{0}]$.

| Node 1 Class | $\boldsymbol{A}$ | $\boldsymbol{B}$ | $k_{1}$ | $x_{0} y_{0} z_{0}$ |
| --- | --- | --- | --- | --- |
| SN(+DC)/SH | 0.3484 0.037 0.1931  0.3296 -0.0243 0.2253  0.3448 0.02285 0.2014 | 0.337 0.0753 0.2019  0.3479 0.079 0.181  0.3426 0.0772 0.1915 | 0.00025  0.0005  0.0005 | 0.40 0 0.07  0.50 0 0.20  0.40 0 0.044 |
| SN(+DC)/SupH | 0.3209 0.0504 0.2353  0.3102 -0.0404 0.2493  0.3155 0.0017 0.2459 | 0.3407 0.0766 0.195  0.3496 0.0795 0.1774  0.3291 0.0727 0.2155 | 0.001  0.001  0.001 | 0.4026 0 0.05  0.498 0 0.267  0.48 0 0.12 |
| SupH/SH | 0.3464 0.0289 0.1979  0.3262 -0.0338 0.229  0.3517 0.0527 0.1831 | 0.3216 0.0475 0.2331  0.3175 0.0139 0.2429  0.3222 0.0565 0.2302 | 0.0001  7.5e-5  0.0001 | -0.63 0 0.1472  -0.607 0 0.1795  -0.633 0 0.1912 |
| SupH/SupH | 0.3171 -0.066 0.2347  0.3115 -0.0546 0.245  0.3166 -0.0654 0.2356 | 0.3177 0.0155 0.2425  0.3129 -0.0141 0.2487  0.3155 0.0017 0.2459 | 2.5e-05  0.00001  0.00003 | -0.6311 0 0.3009  -0.5383 0 0.1058  -0.5911 0 0.2192 |
| SN(-DC)/SH | 0.3006 0.032 -0.2619 0.331 0.0516 -0.2186  0.3163 0.0419 -0.2413 | 0.3462 0.0784 -0.1843  0.2519 0.0487 -0.3069  0.3049 0.0648 -0.2507 | 0.0005  0.001  0.00025 | 0.44 0 0.15  0.44 0 0.09  0.43 0 0.03 |
| SN(-DC)/FLC | 0.1892 -0.0243 -0.3516  0.0069 -0.0819 -0.3915  0.267 0.0126 -0.2976 | 0.3049 0.0648 -0.2507  0.2371 0.0444 -0.319  0.3371 0.0753 -0.2018 | 0.001  0.001  0.001 | 0.47 0 0.15  0.41 0 0.44  0.46 0 0.16 |
| SubH/SH | 0.3125 0.0395 -0.2465  0.3216 0.0454 -0.2335  0.331 0.0516 -0.2186 | 0.1221 0.0104 -0.3808  0.0128 -0.0246 -0.399  0.1658 0.0243 -0.3632 | 0.001  0.001  0.001 | 0.32 0 0.54  0.33 0 0.8  0.35 0 0.42 |
| SubH/FLC | 0.0628 -0.0679 -0.3892  0.267 0.0126 -0.2976  -0.1627 -0.1012 -0.3511 | 0.0312 -0.0189 -0.3983  -0.0171 -0.0336 -0.3982  0.1221 0.0104 -0.3808 | 0.0005  0.001  0.0005 | 0.38 0 0.09  0.33 0 0.68  0.34 0 0.40 |

**Fig A**. **Interspike intervals for each of the 24 node 1 bursting paths implemented.** Paths are grouped by dynamotype and colored by onset. Crosses indicate median values. SN(+DC) = saddle node with baseline shift, supH = supercritical Hopf, subH = subcritical Hopf, SN(-DC) = saddle node without baseline shift, SH = saddle homoclinic, FLC = fold limit cycle.

**Fig B. Eight node 1 dynamotypes and 71 node 2 locations were simulated**. **(A)** Indicates the zoom regions for representative node 1 dynamotype paths in **(C)** LCs and **(D)** LCb. Some bursting paths extend beyond the onset/offset points to close the hysteresis loop. (C) and (D) also show a representative timeseries for each dynamotype. **(B)** Parameter space locations used to model node 2. Open circles and dashed lines are used to indicate a location on the back of the sphere. LCs = limit cycle small, LCb = limit cycle big, SN(+DC)= saddle node with baseline shift, SN(-DC) = saddle node without baseline shift, supH = supercritical Hopf, subH = subcritical Hopf, SH = saddle homoclinic, FLC = fold limit cycle.

**4. Recruitment analysis**

**4.1 Identification of recruitment.** Simulations with at least 10 peaks (spikes) in node 2 were considered for potential recruitment. Removing simulations with less than 10 peaks in node 2 filtered out simulations with only transient responses at the onset and/or offset of coupling. For each simulation with at least 10 peaks, timeseries were high-pass filtered. Mean-square power was calculated for each node during the period with coupling between the nodes. If the node 2 power was at least 10% of the node 1 power, then node 2 was said to be recruited. This thresholding step was performed to discard node 2 responses that were not bursts. Node 2 power was compared to node 1 power to reduce bias toward node 1 bursting paths with greater burst duration or amplitude. The threshold of 10% was chosen based on the observation that the proportion of simulations that resulted in recruitment was relatively stable for power thresholds between 0.1 and 0.8 (Fig C in S1 Methods). Timeseries with power between 0.001 and 0.8 were visualized to confirm the choice of threshold was reasonable.

**4.2 Identification of burst onset and offset.** In this work, we defined burst/seizure onset as the onset of sustained oscillations. In general, burst onset and offset in node 1 were marked by inspecting the sign of the derivative of the slow variable, ż. In the hysteresis-loop implementation, ż changes from positive to negative when node 1 departs from the stable fixed point representing rest; the opposite is true at burst offset (i.e. when node 1 returns to rest). For node 1 dynamotypes with a supH onset bifurcation, hysteresis requires a SN(+DC) bifurcation to move the system into the correct position in state space prior to the onset supH bifurcation. The sign of ż changes after the SN(+DC) bifurcation, but bursting does not start until the supH bifurcation. To isolate the recruitment properties of the supH bifurcation, we adjusted the initial conditions such that the first supH-onset burst could occur without a preceding SN bifurcation. Specifically, we placed the node on the fixed point corresponding to active rest at the start of the simulation. We then defined burst onset as the first negative peak in the timeseries. Thus, node 1 did not influence node 2 through coupling (Eqn. 9) until the supH bifurcation. It should be noted that it is possible to observe periodic bursts in the Multiclass Epileptor that display a supH onset bifurcation without a preceding SN(+DC) bifurcation using paths other than the hysteresis-loop.

In node 2, burst onset was defined as the first negative peak in the timeseries after the onset of coupling. Identification of offset was not required for the analysis. For identification of peaks, MATLAB’s *findpeaks()* was used with a minimum peak prominence of 0.005. Peak prominence is a measure of how much a peak stands out from the surrounding baseline; an arbitrarily low value was set to ensure peaks due to numerical noise were ignored. Peaks that were not prominent and temporally separated from the rest of the bursting pattern were also ignored to remove transients that could occur at the onset of coupling. Peaks were considered ‘not prominent’ if they were below the mean prominence for the simulation. The mean prominence was calculated after discarding the bottom and top 10% to reduce the influence of outliers. To confirm the accuracy of onset times, a third of the data were visualized. Onset was confirmed to be marked accurately in over 99.9% of cases.

**Fig C. Recruitment proportion as a function of power threshold.** Timeseries were high-pass filtered, then mean square power was calculated for each node during the coupling period. Node 2 power was divided by node 1 power and used for identification of recruitment via thresholding. For all dynamotypes, recruitment proportions were consistent for power thresholds between 0.1 and 0.8. Thus, simulations above a power threshold of 0.1 were included in the analyses. SN(+DC) = saddle node with baseline shift, supH = supercritical Hopf, subH = subcritical Hopf, SN(-DC) = saddle node without baseline shift, SH = saddle homoclinic, FLC = fold limit cycle.

**5. References**

1.	Saggio ML, Spiegler A, Bernard C, Jirsa VK. Fast–Slow Bursters in the Unfolding of a High Codimension Singularity and the Ultra-slow Transitions of Classes. J Math Neurosci. 2017;7: 7. doi:10.1186/s13408-017-0050-8

2.	Jirsa VK, Stacey WC, Quilichini PP, Ivanov AI, Bernard C. On the nature of seizure dynamics. Brain. 2014;137: 2210–2230. doi:10.1093/brain/awu133

3.	Izhikevich EM. Neural Excitability, Spiking and Bursting. Int J Bifurc Chaos. 2000;10: 1171–1266. doi:10.1142/S0218127400000840

4.	Sheckler C, Kish K, Walker Z, Barkelew G, Crisp DN, Szuromi MP, et al. Dynamotypes for Dummies: A toolbox, atlas, and tutorial for simulating a comprehensive range of realistic synthetic seizures. eneuro. 2025; ENEURO.0200-25.2025. doi:10.1523/ENEURO.0200-25.2025

5.	Dumortier FAG, Roussarie R, Sotomayor J. Generic 3-parameter families of planar vector-fields, unfoldings of saddle, focus and elliptic-singularities with nilpotent linear parts. 1991. Available: https://api.semanticscholar.org/CorpusID:125805089

6.	Szuromi MP, Jirsa VK, Stacey WC. Optimization of ictal aborting stimulation using the dynamotype taxonomy. J Comput Neurosci. 2023;51: 445–462. doi:10.1007/s10827-023-00859-7

7.	Saggio ML, Crisp D, Scott JM, Karoly P, Kuhlmann L, Nakatani M, et al. A taxonomy of seizure dynamotypes. Skinner FK, Frank MJ, Van Drongelen W, Valiante TA, editors. eLife. 2020;9: e55632. doi:10.7554/eLife.55632

8.	Lopes da Silva F, Blanes W, Kalitzin SN, Parra J, Suffczynski P, Velis DN. Epilepsies as dynamical diseases of brain systems: basic models of the transition between normal and epileptic activity. Epilepsia. 2003;44 Suppl 12: 72–83. doi:10.1111/j.0013-9580.2003.12005.x

9.	Saggio ML, Jirsa VK. Phenomenological Mesoscopic Models for Seizure Activity. In: Scott RC, Mahoney JM, editors. A Complex Systems Approach to Epilepsy: Concept, Practice, and Therapy. Cambridge: Cambridge University Press; 2023. pp. 41–60. doi:10.1017/9781108582285.004

10.	Baier G, Goodfellow M, Taylor PN, Wang Y, Garry DJ. The importance of modeling epileptic seizure dynamics as spatio-temporal patterns. Front Physiol. 2012;Volume 3-2012. doi:10.3389/fphys.2012.00281

11.	Maturana MI, Meisel C, Dell K, Karoly PJ, D’Souza W, Grayden DB, et al. Critical slowing down as a biomarker for seizure susceptibility. Nat Commun. 2020;11: 2172. doi:10.1038/s41467-020-15908-3

12.	Izhikevich EM. Dynamical Systems in Neuroscience: The Geometry of Excitability and Bursting. MIT press; 2007.

13.	Suffczynski P, Lopes da Silva FH, Parra J, Velis DN, Bouwman BM, van Rijn CM, et al. Dynamics of epileptic phenomena determined from statistics of ictal transitions. IEEE Trans Biomed Eng. 2006;53: 524–532. doi:10.1109/TBME.2005.869800
