# Supplementary material for: Seizure recruitment properties are dependent upon dynamotype: A modeling study
Source: PLoS Comput Biol. 2026 Sep 21;22(9):e1013975. doi: 10.1371/journal.pcbi.1013975 (PMC13626473; doi:10.1371/journal.pcbi.1013975)
Supplement: Supplementary file 2 [file pcbi.1013975.s002.docx]

**Supplementary results**

**1. Recruitment properties**

**1.1 Influence of parameter space geometry.** For consistency with prior work [1,2], we constrained the parameters of the fast subsystem of both nodes to the surface of a sphere centered at the origin. However, only the topology of the bifurcation curves in parameter space is guaranteed, by the fact that the fast subsystem is based on the normal form of the unfolding of a bifurcation, and nothing can be said a priori on their geometry, and thus distances. In the present study, the parameter space geometry influenced the distance between the nodes at the onset of coupling. Thus, to mitigate the possibility that the presented results were a byproduct of this specific parameter space geometry, we confirmed the results were not qualitatively influenced by the initial distance between the nodes. First, we verified that node 2 did not tend to be closer to subH or SN(-DC)-onset node 1 dynamotypes compared to SN(+DC)- and supH-onset node 1 dynamotypes (Fig A in S1 Results, panel A). Then, we analyzed the effect of distance on recruitment properties. There were some relationships between distance and recruitment proportion, delay, and energy, but comparisons between SN(+DC)-, supH-, SN(-DC)-, and subH-onset dynamotypes were generally preserved across distance (Fig A in S1 Results, panels B-D).

**1.2 SN followed by SupH-onset dynamotypes.** In the hysteresis-loop implementation, supH-onset dynamotypes cross a SN bifurcation prior to the supH bifurcation. In this work, we adjusted the initial conditions such that the supH-onset dynamotypes did not undergo a SN bifurcation prior to the supH bifurcation. Specifically, we placed the node on the fixed point corresponding to active rest at the start of the simulation. Then, we defined burst onset as the onset of sustained oscillations. In our coupling equations, the coupling term is set to zero until burst onset, meaning that only the supH bifurcation influenced recruitment for supH-onset node 1 dynamotypes. This choice of onset definition facilitated study of the supH-onset bifurcation in isolation. To examine the recruitment properties of the SN(+DC) and supH onset bifurcations in sequence, we re-ran the simulations for supH-onset dynamotypes with a different set of initial conditions, such that a SN bifurcation preceded the supH bifurcation. For these simulations, we defined burst onset as the first departure from rest, i.e. the SN bifurcation. We found that supH-onset node 1 dynamotypes still displayed low recruitment proportion, especially compared to SN(-DC)- and subH-onset node 1 dynamotypes (Fig B in S1 Results, panel A). All comparisons remained statistically significant. Recruitment delay remained variable and significantly lower than that of SN(+DC)-onset node 1 dynamotypes, but was significantly higher than SN(-DC)- and subH-onset node 1 dynamotypes (Fig B in S1 Results, panel B). Recruitment energy became significantly higher than that of SN(-DC)- and subH-onset node 1 dynamotypes and similar to that of SN(+DC)-onset node 1 dynamotypes (Fig B in S1 Results, panel C). All statistical comparisons were done via Kruskal-Wallis test followed by multiple comparisons with Bonferroni correction at a significance level of 0.001. Recruitment proportion increased with coupling strength, but only for high node 1 excitability (Fig C in S1 Results). Thus, the SN(+DC) followed by supH onset bifurcation sequence demonstrated recruitment properties between those of SN(+DC) and supH onset bifurcations in isolation.

**1.3 Influence of coupling term.** Finally, to ensure the results were robust to choice of coupling term, we re-ran the simulations after replacing ${G(x_{1}-x_{1, rest})}^{2}$with $G\sqrt{\left( x_{1}-x_{1, rest} \right)^{2}+\left( y_{1}-y_{1, rest} \right)^{2}}$. We found only subtle changes in the results (Figs D, E in S1 Results).

**Fig A. Influence of distance on recruitment properties. (A)** Distributions of distances between node 1 and node 2 at the onset of coupling, with respect to node 1 onset bifurcation. Node 2 initial locations were not closer to SN(-DC) or subH-onset node 1 dynamotypes. Influence of distance on **(B)** recruitment proportion, **(C)** log of normalized recruitment delay, and **(D)** log of recruitment energy. While there are some consistent trends, particularly at distances < 0.2 AU, differences in recruitment properties across node 1 dynamotypes are generally preserved. In (A), proportions were computed in 40 equally spaced distance bins (bin width = 0.02). In (B) and (C), moving averages were computed with a window size of 300. SN(+DC) = saddle node with baseline shift, supH = supercritical Hopf, SN(-DC) = saddle node without baseline shift, subH = subcritical Hopf.

**Fig B. Recruitment properties with respect to node 1 onset bifurcation.** For these results, supH onset bifurcations were preceded by SN(+DC) bifurcations and burst onset was defined as the first departure from rest. Thus, for supH-onset node 1 dynamotypes, coupling began at the preceding SN(+DC) bifurcation. **(A)** Recruitment proportion. Each datapoint represents one node 2 location. All pairwise comparisons were statistically significant. **(B)** Log of normalized recruitment delay and **(C)** log of recruitment energy. Each datapoint represents one simulation. In (B), SN(+DC)-onset node 1 dynamotypes were associated with significantly higher recruitment delays than all other node 1 dynamotypes. Also, supH-onset node 1 dynamotypes had significantly higher delays than SN(-DC)- and subH-onset node 1 dynamoytpes. In (C), all pairwise comparisons were statistically significant, except for SN(+DC)- compared to supH-onset node 1 dynamotypes. Statistical significance was evaluated via Kruskal-Wallis tests followed by multiple comparisons with Bonferroni correction at a significance level of 0.001. SN(+DC) = saddle node with baseline shift, supH = supercritical Hopf, SN(-DC) = saddle node without baseline shift, subH = subcritical Hopf.

**Fig C. Recruitment proportion versus coupling strength (G) and node 1 excitability (d*).** For these results, supH onset bifurcations were preceded by SN(+DC) bifurcations and burst onset was defined as the first departure from rest. Thus, for supH-onset node 1 dynamotypes, coupling began at the preceding SN(+DC) bifurcation. **(A)** Results for all simulations. **(B)-(E)** Results for each node 1 onset bifurcation. SN(+DC) = saddle node with baseline shift, supH = supercritical Hopf, SN(-DC) = saddle node without baseline shift, subH = subcritical Hopf.

**Fig D. Recruitment properties with respect to node 1 onset bifurcation.** For these results, the coupling term was modified to ensure results were robust to choice of coupling term. **(A)** Recruitment proportion. Each datapoint represents one node 2 location. **(B)** Log of normalized recruitment delay and **(C)** log of recruitment energy. Each datapoint represents one simulation. In (A) and (C), all pairwise comparisons were statistically significant. In (B), SN(+DC)-onset node 1 dynamotypes were associated with significantly higher recruitment delays than all other node 1 dynamotypes. Also, SN(-DC)-onset node 1 dynamotypes had significantly higher delays than supH- and subH-onset node 1 dynamotypes. Statistical significance was evaluated via Kruskal-Wallis tests followed by multiple comparisons with Bonferroni correction at a significance level of 0.001. SN(+DC) = saddle node with baseline shift, supH = supercritical Hopf, SN(-DC) = saddle node without baseline shift, subH = subcritical Hopf.

**Fig E. Recruitment proportion versus coupling strength (G) and node 1 excitability (d*).** For these results, the coupling term was modified to ensure results were robust to choice of coupling term. **(A)** Results for all simulations. **(B)-(E)** Results for each node 1 onset bifurcation. SN(+DC) = saddle node with baseline shift, supH = supercritical Hopf, SN(-DC) = saddle node without baseline shift, subH = subcritical Hopf.

**2. Propagation zone behavior**

In the main text, we identified factors that impact the behavior of the propagation zone. Namely, recruitment through mechanism A facilitated onset dynamics in node 2 that matched those of node 1, while recruitment through mechanism B could enable mismatches in the onset dynamics of the nodes. In addition, changes in the location of the stable fixed point in parameter space could lead to baseline drifts in node 2, even in the absence of a change in baseline in node 1. Finally, for simulations with sufficiently high coupling strength, temporary violation of timescale separation promoted unusual dynamics in node 2. Here, we summarize the propagation zone behaviors enabled by these processes for each node 1 onset bifurcation, with comparisons to clinical EEG. We combine SN(-DC) and subH bifurcations into one group because they are indistinguishable in timeseries data [3]. As a reminder, SN(+DC)-, supH-, and SN(-DC)- or subH-onset node 1 dynamotypes demonstrate baseline shifts, amplitude scaling, and arbitrary amplitude at onset, respectively.

**2.1 SN(+DC)-onset node 1 dynamotypes.** The model predicted that node 2 could exhibit all possible onset properties: a baseline shift, arbitrary amplitude, amplitude scaling, or a baseline drift followed by amplitude scaling (Fig 8). Recruitment through mechanism B and changes in the location of the fixed point facilitated the range of onset dynamics seen in node 2. Clinical examples confirmed the potential for these propagation behaviors (Fig 9, S3).

**2.2 SupH-onset node 1 dynamotypes.** Recruitment from active rest through mechanism B promoted amplitude scaling at onset in node 2, as in node 1 (Fig F in S1 Results, panel A). The seizure in Fig F in S1 Results, panel C confirms the possibility for this combination of seizure onset and propagation zone dynamics. Model predictions and clinical examples also demonstrated the potential for node 2 to exhibit changes in baseline and/or arbitrary amplitude (Figs 10, 11; Fig F in S1 Results, panel B). Changes in baseline were possible through drifts in the location of the fixed point. Arbitrary amplitude was enabled by violation of timescale separation.

**2.3 SN(-DC)- and subH-onset node 1 dynamotypes.** Recruitment through mechanism A enabled in arbitrary amplitude in node 2 (Fig G in F1 Results, panel A), which we confirmed was possible in clinical data (Fig G in F1 Results, panel D). Recruitment of nodes from active rest via the ictal-only region promoted amplitude scaling at onset (Fig G in F1 Results, panel C). If node 2 moved quickly across the supH and subH bifurcations, supH onset characteristics could be lost (Fig G in F1 Results, panel B), resulting in arbitrary amplitude. Baseline drifts smaller than the amplitude of the oscillations were also possible in node 2 (Fig G in F1 Results, panels B, C). The potential for amplitude scaling at node 2 onset is confirmed by the clinical example in Fig G in F1 Results, panel E. Here, there also may have been a baseline drift >5 sec prior to the onset of sustained oscillations. We did not witness an example of a baseline drift temporally separated from supH onset dynamics in node 2 when node 1 was subH- or SN(-DC)-onset in the model. However, node 2 could display this behavior if it began in rest was pulled to active rest and then into the ictal-only region across the supH bifurcation. This path did not occur in the simulations presented here because node 2 moved along the shortest path to node 1 at each point in time. A slightly longer path could produce the observed sequence.

**Fig F. Additional propagation zone behaviors for supH-onset node 1 dynamotypes. (A)** and **(B)** Model predictions. Shaded bars under the timeseries indicate the parameter space region of node 2. SN = saddle node, supH = supercritical Hopf, subH = subcritical Hopf, SH = saddle homoclinic, FLC = fold limit cycle. **(C)** Clinical seizure propagation example analogous to (A). Increasing amplitude at onset suggests supH onset bifurcations in all channels. Numbers indicate electrode contacts; all contacts were located on a grid electrode placed over the right lateral temporal region. R = right.

**Fig G. Propagation zone behaviors for SN(-DC)- and subH-onset node 1 dynamotypes. (A)-(C)** Model predictions. Shaded bars under the timeseries indicate the parameter space region of node 2. SN = saddle node, supH = supercritical Hopf, subH = subcritical Hopf, SH = saddle homoclinic, FLC = fold limit cycle. **(D)** Clinical seizure propagation example analogous to (A). At onset, amplitude is arbitrary in all channels, consistent with subH or SN(-DC) onset bifurcations. Note that although there is increasing amplitude, particularly in the right middle hippocampus, this does not occur until more than 15 seconds after the seizure began. That increasing amplitude was not part of the initial bursting pattern and likely represents volume conduction from neighboring tissue. R = right. **(E)** Clinical seizure propagation example analogous to (C). The onset channels display arbitrary amplitude at onset, compatible with a subH or SN(-DC) onset bifurcation. The propagation channels display increasing amplitude at onset, suggesting a supH onset bifurcation. There is also potentially a baseline shift without bursting before the supH onset. Numbers indicate electrode contacts. L = left.

**3. References**

1.	Saggio ML, Spiegler A, Bernard C, Jirsa VK. Fast–Slow Bursters in the Unfolding of a High Codimension Singularity and the Ultra-slow Transitions of Classes. J Math Neurosci. 2017;7: 7. doi:10.1186/s13408-017-0050-8

2.	Szuromi MP, Jirsa VK, Stacey WC. Optimization of ictal aborting stimulation using the dynamotype taxonomy. J Comput Neurosci. 2023;51: 445–462. doi:10.1007/s10827-023-00859-7

3.	Saggio ML, Crisp D, Scott JM, Karoly P, Kuhlmann L, Nakatani M, et al. A taxonomy of seizure dynamotypes. Skinner FK, Frank MJ, Van Drongelen W, Valiante TA, editors. eLife. 2020;9: e55632. doi:10.7554/eLife.55632

4.	Izhikevich EM. Neural Excitability, Spiking and Bursting. Int J Bifurc Chaos. 2000;10: 1171–1266. doi:10.1142/S0218127400000840
