# Supplementary material for: Seizure recruitment properties are dependent upon dynamotype: A modeling study
Source: PLoS Comput Biol. 2026 Sep 21;22(9):e1013975. doi: 10.1371/journal.pcbi.1013975 (PMC13626473; doi:10.1371/journal.pcbi.1013975)
Supplement: Supplementary file 3 [file pcbi.1013975.s009.docx]

**Response to the Reviewers**

We would like to thank the reviewers for their detailed feedback on our manuscript. Please see our detailed responses below. Text references are given as

Section, subsection, paragraph (page number, line number in manuscript file without track changes, if applicable).

*Text*

**Note on Code Availability**

We have updated our code. Specifically, file names have been changed to match figure numbers. We also added statistical analysis to the files corresponding to Fig 5 and Fig 6. Minor changes have been made to the code for S7 Fig in S2 Supplementary Results. We will upload the revised code with those minor changes to Deep Blue Data Repository when we know no further changes are required. In case any of the reviewers would like to access the updated code, we have uploaded the scripts corresponding to the figures with our resubmission in S1 Code. All other code was unchanged and can be found using the provided link to Deep Blue Data Repository.

**Reviewer #1:**

1. No stochasticity or measurement noise is incorporated; robustness of recruitment predictions to realistic noise remains untested.

We agree with the reviewer that the absence of noise is a limitation of the model. This choice was deliberate. Without noise, we could more directly evaluate the influence of dynamotype alone on recruitment, which was the primary goal of this work. However, we would expect noise, unless at low levels, to impact the results. Future work will evaluate the effects of noise. Our recent publication (Sheckler et al., eNeuro, 2025) included a method to include noise in this model (both stochasticity and measurement noise), so we readily can perform such studies numerically. We are actively exploring how different types of noise affect seizure onsets at the single node level, which will be the subject of future publications. At a single node level, adding realistic levels of noise (human EEG is very noisy) dominates the results unless at trivially low levels. Given the sensitivity of the model to noise, it is paramount that the modeled noise have realistic characteristics. Unfortunately, we are unaware of prior work that might give rigorous guidance about the dynamical properties of noise in the context of our model, and thus such work requires careful validation that is outside the scope of the current paper. We note that it is common in prior studies using similar dynamical models to explore the responses with low or no noise first, which allows isolation of the model’s behavior rather than discovering how noise will deviate from that behavior (see Proix et al., Journal of Neuroscience, 2014; Jirsa et al., NeuroImage, 2017; Saggio et al., PLOS Computational Biology, 2024). As our results are a novel exploration of the effects of coupling, we feel it is most important to establish the essential behavior without noise in similar fashion. We added the following paragraph to the discussion to address this limitation:

Discussion, Model Limitations, Paragraph 3 (Page 33, Line 790):

*We also did not include noise in our simulations. In this work, we assume that seizure onset is precipitated by a bifurcation, and we consider the influence of bifurcation type on recruitment. For this reason, all simulations are performed without noise. This is in line with previous studies using the Epileptor [23,24,32], in which only low levels of additive noise are used to improve the realism of the simulations without altering the key dynamics. However, the inherently noisy nature of the brain and the fact that stronger or more complex noise can alter the dynamics of the model justifies future efforts to characterize the impact of noise. A first consequence of including noise at the single-node level (see [28] for a method), is that noise-induced transitions become possible in the presence of bistability [16]. The coupled nodes could therefore demonstrate either bifurcation- or noise-induced transitions within the bistable regions of parameter space. Other expected effects of noise in this model include excitable and chaotic dynamics.*

*While evaluating the impact of noise on propagation is certainly a subject for future work, here we first establish the influence of dynamotype alone.*

2. Human data serve primarily as proof-of-concept illustrations. Validation on human data is qualitative and limited to a few examples; no quantitative assessment (e.g., rates of predicted mismatches, delay distributions, or dependence on onset patterns) is provided.

We agree that the comparisons are proof-of-concept, and there are two reasons for this. First, it is critical to point out that these data are presented from a clinical viewpoint. Some of the examples are patterns seen commonly in human intracranial EEG, which are presented to show how such patterns can be explained via the lens of dynamotypes, i.e. why does the recruited node have different dynamics than the onset node, and how can we describe it? But more importantly, other examples are highly unusual patterns that clinicians might consider impossible, such as a DC shift in the recruited node but not the initial node. Indeed, our clinician author (WS) thought such a prediction impossible, and challenged the first author (DK) to find examples to show that such a situation was even plausible. Thus, while such examples may appear anecdotal, they serve a critical purpose: when disproving the hypothesis that something is impossible (or proving a high-risk hypothesis that something is plausible), a few examples are sufficient to establish plausibility. We found multiple examples even in our limited dataset, showing that these situations were uncommon but certainly found in nature. Thus, the examples serve two purposes: as visual demonstrations to show how our modeled data could translate to human EEG, and as proof that some of our unusual predictions can be found in nature.

Second, while we are certainly interested in applying these predictions to human data, it is also critical to point out the scope that would be necessary to prove these predictions rigorously. This will be a massive project far outside the scope of this initial study. We estimate needing thousands of seizures from intracranial EEG, each with the onset time and dynamotype marked on every channel. Thousands of seizures are necessary because many of the dynamotypes are much less common in humans, as we showed in our prior work (Saggio et al., eLife, 2020). Then, these studies usually have >100 channels, all of which must be categorized for onset time and type as well. Currently, this can only be done via visual analysis by a trained rater. In addition, the volume of tissue corresponding to each electrode/group of electrodes would need to be estimated, as well as the distance between every electrode pair. To be rigorous, this must account for the sylvian fissure and the intrahemispheric fissure at least, and potentially for white matter tracts. Further, the number of electrodes and electrode locations vary between patients; hence, a method of normalizing propagation analysis across patients would need to be developed. This will require literally thousands of hours of work. This model-centric approach thus fulfills one of the classic roles of computational modeling—to perform the impossible experiment and motivate future work when the experiments become feasible. We are developing automated tools to speed up portions of this work, but they also must be validated on many hundreds of seizures that are similarly marked. These endeavors must be justified by computational modeling. The current paper motivates that validation work in the future.

We clarified the intention of our comparisons to human data with the following addition to the discussion:

Discussion, Model Limitations, Paragraph 4 (Page 34, Line 812):

*These examples are not meant to be a robust validation of the predictions; rather, they are a demonstration that the recruitment behaviors predicted using our propagation model, several of which were unusual and unexpected, were plausible and could be found even in our dataset of under 200 patients. Robust validation of all model predictions would require labeling exact onset times and compatible bifurcations in every channel (up to hundreds of channels) for tens to hundreds of seizures for every dynamotype in large human EEG datasets, a task far beyond the scope of this initial study. Our results motivate that future work, as well as the development of automated multi-channel dynamotype classification methods to enable such an undertaking.*

3. The coupling function for node 2’s slow variable is ad hoc (proportional to (x_1_ − x_1,rest_)^2^), with no comparison to alternative fast-to-slow or slow-to-slow couplings, saturation effects, or normalization across dynamotypes.

We thank the reviewer for bringing up this important point. We chose to implement fast-to-slow coupling to model seizure propagation to account for long recruitment times (up to seconds), and due to the success of fast-to-slow coupling in modeling seizure networks using the (single-class) Epileptor. In models with only fast variables, which rely on noise or inputs for seizure initiation, this alteration of the baseline parameter is achieved by fast-to-fast coupling which directly affect the excitability parameter in the fast equation (see for example Goodfellow et al., Scientific Reports, 2016; Sinha et al., Brain, 2017; Laiou et al., Frontiers in Neurology, 2019; Hutchings et al., PLOS Computational Biology, 2015). In the Epileptor model, which has fast-slow dynamics and in which the excitability parameter is on the slow equation, this could be achieved by fast-to-slow coupling. Our novelty is that the fast-to-slow coupling affects the parameters of the fast equations, effectively steering the second node in its parameter space and allowing for a variety of dynamics. We have expanded our discussion to further justify our use of fast-to-slow coupling while acknowledging that coupling on other timescales may also occur in seizure propagation. In doing so, we also addressed comment #6. The relevant paragraph now reads:

Discussion, Rationale for model development, Paragraph 3 (Page 29, Line 699):

*To connect two nodes of the model, we implemented a novel form of fast-to-slow coupling, allowing fast activity in node 1 (the seizure onset zone) to alter the dynamics of node 2 (the propagation zone). Fast-to-slow coupling has been used to connect brain regions with identical dynamics using the Epileptor in toy [24] and patient-specific [23,25,32] models of seizure propagation. Fast-to-slow coupling models the influence of fast variables (e.g. neural firing) in one brain region on slow variables (e.g. ion concentrations, ATP, tissue oxygenation) in another brain region. The perturbation of the slow variables may occur through any physiological process (e.g. synaptic transmission, ephaptic connections, gap junctions, or a combination therein), directly or indirectly. For example, a change in the homeostatic variables, like extracellular potassium concentration, in node 1 can indirectly alter those in node 2. Compared to fast-to-fast coupling, fast-to-slow coupling may better account for long recruitment times (up to seconds) during seizure propagation [24,25]. Further, fast-to-slow coupling has been shown to better capture patients’ seizure networks [25] than fast-to-fast coupling, although the timescale of coupling was not the most important factor in that study. Here, we extend fast-to-slow coupling to connect brain regions with diverse dynamics within the Multiclass Epileptor. While models implementing bifurcations as onset mechanisms have primarily used fast-to-slow coupling [23–25,32], fast-to-fast coupling is standard in models relying on noise to initiate a seizure [19–22,38,39]. The different coupling methods in the literature are not mutually exclusive. Indeed, a combination of coupling on different timescales may best predict the spatiotemporal diversity of propagation patterns in epilepsy [31]. Future work will investigate the interaction of coupling on different timescales in the Multiclass Epileptor.*

We thank the reviewer for pointing out our lack of justification for the coupling function and limited discussion of normalization across dynamotypes. To compare results across dynamotypes, we included normalization in our calculation of recruitment proportions, delays and energy. To be considered ‘recruited,’ the mean-square power of node 2 had to be at least 0.10 that of node 1 during the coupling period. Comparing to node 1 power reduces bias towards longer bursting paths and bursting paths with greater amplitude oscillations. For both recruitment delay and energy calculations, time was expressed as a proportion relative to node 1’s total burst duration. The speed (k) of movement along each node 1 bursting path varied, resulting in a different number of discrete time points per node 1 path. Hence, this normalization prevented the delay and energy values from being elevated for ‘slower’ bursting paths. We now provide additional sentences of emphasis on these normalizations in the methods. We previously performed a separate analysis using a different function that had similar results (now in S1 Supplementary Methods). The function during the coupling period was as follows: $G\sqrt{\left( x_{1}-x_{1, rest} \right)^{2}+\left( y_{1}-y_{1, rest} \right)^{2}}$.

To further justify the coupling function, we have added the following paragraph to the discussion:

Discussion, Rationale for model development, Paragraph 4 (Page 30, Line 721):

*The novelty of our implementation is that, during coupling, the dynamics (parameter values) of the potential propagation zone are altered towards that of the onset zone. For this first exploration of recruitment among nodes with diverse dynamics, we moved node 2 along an arc path to node 1. This is the simplest method, but more complicated routes may be possible. As a first approximation, we chose the speed of movement of node 2 towards node 1 to be proportional to the square of the difference from rest in node 1’s x variable (Eqn. 3). In developing this coupling function, we reasoned that the novel activity in the seizure onset zone (i.e. the electric field generated, represented by the change in voltage: x1 – x1, rest) influences the propagation zone. The coupling is phenomenological and thus effectively summarizes the cumulative effect of a variety of processes which depend on the specific conditions and locations of the brain regions involved. We squared the change in voltage to avoid biasing recruitment towards bursting paths in LCs (which have a distinct separation between bursting and resting states; see [13]) compared to bursting paths in LCb (in which oscillations cross the resting state). To further ensure that the coupling function was not biasing results, we calculated the area under the entire coupling term G(x1 – x1, rest)2 during the period between node 1 and node 2 onset. We called this value the recruitment energy. If the coupling function biased recruitment towards certain dynamotypes, we would expect dynamotypes with elevated recruitment proportions to also demonstrate elevated recruitment energy. However, there was no consistent relationship between recruitment proportion and recruitment energy among node 1 onset bifurcations (Fig 5). We therefore infer that the differences in recruitment proportion are not due to biases or saturation effects introduced by the choice of coupling term. We also found no major difference in results with a different coupling function (S2 Supplementary Results). Nevertheless, other choices for the coupling term could be equally relevant and are an area for future study.*

In addition, this reviewer may be interested in our deeper explanation of our choice of coupling in our response #15 to Reviewer 3.

4. Mechanism A can require temporary violation of fast–slow timescale separation, but the implications of this assumption for model validity are not deeply examined.

We thank the reviewer for suggesting greater discussion of violation of fast-slow timescale separation. We have added additional details to our discussion, as shown below. Specifically, we now put greater emphasis on the mathematical impact of broken timescale separation and the consequences for node 2’s behavior. Additionally, we point out that recruitment behaviors achieved only through broken timescale separation were also found in clinical seizure propagation examples, providing additional physiological rationale. We also want to point out that clinical seizure recruitment occurs near-instantly in some cases and up to 3-5+ seconds later in other cases. We are able to account for both of these scenarios in our propagation model by altering the coupling factor (G), where increasing G generally reduces the recruitment delay. Breaking timescale separation is necessary with this form of coupling to describe cases where a region of the brain that never seizes on its own is recruited within milliseconds. Thus, while it is a non-ideal application of the model, it is plausible that this is what actually happens in vivo.

The full discussion paragraph now reads:

Discussion, Model limitations, Paragraph 1 (Page 31, Line 748):

*A key aspect of the Multiclass Epileptor is timescale separation between the fast and slow subsystems. Timescale separation follows from the progression toward seizure termination, over the course of seconds or minutes, being much slower than the oscillations during the ictal phase. With fast-to-slow coupling, timescale separation holds only for small values of coupling strength. However, we chose to investigate a wider range of coupling strengths, meaning that timescale separation did not hold in all examples of our propagation model. Specifically, timescale separation is temporarily broken for node 2 between node 1 onset and when node 2 reaches node 1, when coupling strength is sufficiently high. During the period of broken timescale separation, the topology of the parameter space map and invariant properties of bifurcations may not hold. As such, node 2’s EEG (x variable) may show unexpected behavior. Most obviously, node 2 may cross a bifurcation without demonstrating that bifurcations’ properties (scaling laws, presence/absence of baseline shifts). This further complicates identification of onset bifurcation in clinical data. Note that timescale separation is only broken for node 2, and only during the initial recruitment period. There is physiological rational for this situation. When a clinical seizure begins in the seizure onset zone, the propagation zone may be recruited quickly – often within milliseconds [38]. That recruitment can span the entirety of the brain, and it is unlikely every brain node is always at the same dynamical baseline. That the seizure onset zone can quickly recruit propagation zones from a range of initial dynamical conditions (e.g. Fig 9; evidenced by different onset behavior in each propagation zone) suggests that temporary breakage of timescale separation is plausible. Further, violation of timescale separation was required to produce certain recruitment behaviors. For example, node 2 could cross a supH bifurcation but lack amplitude scaling (as in Fig 11A) if the movement on the map was sufficiently fast, enabling arbitrary amplitude in node 2 when recruited by a supH-onset node 1. The propagation pattern in the human seizure in Fig 11B is consistent with this recruitment behavior.*

5. The return dynamics for node 2 (constant speed k2) may bias post-recruitment trajectories; physiological plausibility of this choice is not established.

We completely agree. That node 2 returns to its initial location in parameter space is supported by the clinical observation that seizures tend to follow a stereotyped progression in a given patient. If the propagation zones did not return to their starting locations in parameter space, then we would expect each seizure to propagate differently. Nevertheless, the specific choice of *how* node 2 returns was arbitrary. Importantly, none of the presented results depend on post-recruitment trajectories. We only consider the time period with coupling for evaluation of whether recruitment occurred, and if so, recruitment delay and energy. For analysis of recruitment behavior, we only consider the onset of node 2, which occurred during the node 1 burst. We do not report results that depend on the return dynamics. We added a few sentences to the methods to emphasize these points:

Model and methods, Connecting seizure onset and propagation zones, Paragraph 5 (Page 12, Line 268):

*That node 2 has an innate tendency to return to its initial location in parameter space is supported by the clinical observation that seizures tend to follow a stereotyped progression in a given patient. If propagation zones did not return to their “preferred” parameters, then we would expect each seizure to propagate differently. We implemented a simple method to return node 2 to its initial location in parameter space; however, note that none of our analyses were dependent on post-coupling trajectories.*

6. Requires a deeper comparison to Epileptor field modeling of propagation and recruitment mechanisms (e.g., Proix et al., 2017), including differences between fast vs slow permittivity couplings and the role of LVFA-mediated coupling suppression.

We completely agree. We added additional details to the discussion to compare fast vs slow coupling:

Discussion, Rationale for model development, Paragraph 3 (Page 29, Line 699):

*To connect two nodes of the model, we implemented a novel form of fast-to-slow coupling, allowing fast activity in node 1 (the seizure onset zone) to alter the dynamics of node 2 (the propagation zone). Fast-to-slow coupling has been used to connect brain regions with identical dynamics using the Epileptor in toy [24] and patient-specific [23,25,32] models of seizure propagation. Fast-to-slow coupling models the influence of fast variables (e.g. neural firing) in one brain region on slow variables (e.g. ion concentrations, ATP, tissue oxygenation) in another brain region. The perturbation of the slow variables may occur through any physiological process (e.g. synaptic transmission, ephaptic connections, gap junctions, or a combination therein), directly or indirectly. For example, a change in the homeostatic variables, like extracellular potassium concentration, in node 1 can indirectly alter those in node 2. Compared to fast-to-fast coupling, fast-to-slow coupling may better account for long recruitment times (up to seconds) during seizure propagation [24,25]. Further, fast-to-slow coupling has been shown to better capture patients’ seizure networks [25] than fast-to-fast coupling, although the timescale of coupling was not the most important factor in that study. Here, we extend fast-to-slow coupling to connect brain regions with diverse dynamics within the Multiclass Epileptor. While models implementing bifurcations as onset mechanisms have primarily used fast-to-slow coupling [23–25,32], fast-to-fast coupling is standard in models relying on noise to initiate a seizure [19–22,38,39]. The different coupling methods in the literature are not mutually exclusive. Indeed, a combination of coupling on different timescales may best predict the spatiotemporal diversity of propagation patterns in epilepsy [31]. Future work will investigate the interaction of coupling on different timescales in the Multiclass Epileptor.*

The role of LVFA-mediated coupling suppression is not captured in our propagation model. The Multiclass Epileptor does not have a specific representation of LVFA (although it often has a DC shift, in which it would be classified as a Saddle Node +DC). Further, we implemented one-directional coupling from the excitable onset zone to the non-excitable propagation zone, meaning we did not consider suppression effects. We newly acknowledge this limitation in the following paragraph in the discussion:

Discussion, Model limitations, Paragraph 2 (Page 32, Line 774):

*To focus on the dynamical conditions under which a seizure onset zone is more likely to recruit a propagation zone, we implemented a toy model of seizure propagation. While the use of such a toy model facilitated the study of dynamical factors and recruitment in isolation, it is a simplification of clinical seizure propagation. In our toy model, coupling was one-directional from a single, epileptogenic seizure onset zone to a single, non-epileptogenic propagation zone. We did not consider complex feedback that can occur in larger network models or with reciprocal coupling, thereby negating suppression effects.*

7. Limited discussion of how this framework complements patient-specific TVB-based recruitment models and emerging multi-scale co-simulation approaches (e.g., Arbor–TVB), or analytic advances in neural mass model inversion across phase-space regimes.

We completely agree. While those models incorporate increasingly complex physiological models of coupling, they also assume all nodes have the same dynamical signature (e.g. the TVB is typically run with a single class Epileptor at each node). The goal of the current study was not to explore the effects of white matter connectivity, but rather to explore how different dynamics at each node would affect propagation, which as the reviewer points out is complementary to those other models. In essence, this work helps determine whether it would be necessary to incorporate the Multiclass Epileptor, rather than single class Epileptor, in future TVB-like modeling. We added additional details to the discussion, as shown below.

Discussion, Toward improved analysis of seizure networks, Paragraphs 1 and 2
(Page 36, Line 869):

*A contributing factor to poor outcomes in resective surgery for drug-resistant focal epilepsy is a lack of understanding of patients’ seizure networks. A novel method used to better delineate onset and propagation zones is the use of the virtual epileptic patient (VEP), which seeks to recreate patients’ seizure networks in a computational model based on electrographic and imaging data [23,32]. Thus far, nodes in VEPs have been populated with Epileptors, which model the SN(+DC)/SH dynamotype. VEPs are created within the virtual brain framework [30], a platform for large-scale brain network models with realistic connectivity. Neural mass models other than the Epileptor can be implemented in the virtual brain framework [30]. Methods for multiscale simulation – i.e. combining phenomenological neural mass models with biophysical neuron models – in the virtual brain are an area of active development [42]. Patient-specific whole-brain seizure network models like the VEP seek to estimate the seizure onset zone and predict the outcomes of resective surgery, with an ultimate goal of providing a presurgical planning tool.*

*In demonstrating that seizure recruitment properties depend upon dynamotype, we motivate the inclusion of a range of dynamotypes in future whole-brain network dynamical models of epilepsy. Seizure dynamotypes are known to vary between patients and over time [16,18]. One seizure can also involve diverse dynamics in different areas of the brain (e.g. Fig 9). Thus, the inclusion of a wider range of dynamics in large scale dynamical models may provide more accurate and patient-specific results. There are several options for implementing the inclusion of richer dynamical detail in seizure network models. A recent analysis revealed that minor modifications of the Epileptor model can enable some additional bifurcations, albeit not all the bifurcations present in the Multiclass Epileptor [43]. The Epileptor is already integrated into the VEP workflow, so minor modifications may facilitate an increase in dynamical diversity without requiring major updates to the remainder of the pipeline. Another option is to populate each node in a seizure network model with a Multiclass Epileptor, which would capture a greater range of dynamotypes possible in epileptic brain. The cost of this greater dynamical detail would be more computationally intensive model inversion, given that the Multiclass Epileptor involves a larger parameter space. It is worth noting that despite increased computational complexity, current model inversion techniques can be successful even in high dimensional parameter spaces [44]. To reduce computational requirements, it may be helpful to identify the nearby bifurcations of each node in the virtual brain to partially constrain the choice of bursting path. This can be done through analysis of ictal timeseries [16] or in theory through perturbation analysis [17].*

**Reviewer #2**

8. Weak Link Between Model Assumptions and Biological Reality

The primary weakness of this study is the tenuous link between the mathematical assumptions of the Multiclass Epileptor and physiological reality. The phenomenological nature of the model eclipses the theoretical advances the paper claims to make. For instance, the model predicts that seizures with a baseline shift at onset are less likely to spread. However, without a clear mapping of what a "baseline shift" or specific bifurcation means in terms of underlying tissue excitability, ion concentrations, or synaptic kinetics, this prediction lacks biological grounding. In the clinical reality, a baseline shift can be a signal artifact, might depend on the recording location, electrode type and amplifier settings, with extensive literature in neurophyiology discussing this decades ago. Consequently, the clinical relevance of the model's predictions is highly questionable. The authors bury several key model assumptions in the text, making it difficult to assess the validity of their claims.

While we agree with the reviewer that the Multiclass Epileptor is a phenomenological model without a direct link to biology, we view this as a strength because it describes behaviors that are invariant to any specific physiology. While this may seem counterintuitive, it is well-established in the dynamical field. For instance, when using bifurcation theory to assess whether a structure is going to break or an earthquake/avalanche/volcano is going to start, the precise materials involved or cause of the change is not necessary to measure *how the system behaves.* While mechanistic information is clearly important, it is not necessary to describe the dynamics. This is precisely the method we have implemented here. Dynamical models like the Multiclass Epileptor capture invariant properties that are present regardless of the underlying epileptogenic mechanism.

In seizures, this approach has a crucial benefit: it overcomes the problem of degeneracy. Degeneracy describes conditions in which a system has multiple potential pathways to produce the same output, and it has been described extensively by Eve Marder over the past 15 years. Seizures are extremely degenerate: there are essentially an uncountable number of potential pathologies that generate seizures. Seizures are thus a final common pathway that yields very little insight into the underlying pathology. The result is that if you investigate the effects of one pathology on seizures (e.g. one animal model), your results may only pertain to that model.

The degenerate nature of seizures, and the ability of our model to reconcile it, was explained in great detail in two prior papers that introduced this model in epilepsy. In those papers, we provided extensive validation of this model for epilepsy. In the first (Jirsa et al. Brain 2014) we demonstrated that similar offset dynamics were conserved across species (bats, fruit flies, humans), human pathology (multiple patients with different locations and pathologies), and even propagation methods (non-synaptic seizures in zero calcium). In the second (Saggio et al., eLife, 2020), we identified seizures compatible with different dynamotypes via analysis of ictal EEG in humans. We identified signatures consistent with all dynamotypes in human EEG, that individual patients demonstrate different dynamotypes over time in the same location, and that epilepsy pathology is unrelated to the underlying dynamotype. We consider this model to be sufficiently validated to allow us to use it to make further predictions in this work, which will lead to future validation studies (see response to #9).

The result is that our model deliberately does not attempt to ascribe any behavior to a specific pathology, but that is not a weakness. The model is not attempting to explain the mechanism of a particular seizure; instead, it is describing how a seizure behaves if it has certain dynamical characteristics. If a seizure starts with a DC shift (saddle node bifurcation) vs. slowly increasing amplitude (supH bifurcation), the model predicts that the voltage output itself has key dynamical characteristics, regardless of the underlying pathology. Indeed, from the standpoint of a “node 2” that is not involved in the seizure generation, it is unlikely that the underlying physiology that caused “node 1” to seize matters—all that matters is the input to the neurons in node 2. This allows a much simpler analysis that is clinically implementable (i.e. measure the voltage with EEG, without need to assess ions/proteins/synaptic connectivity that would be impractical in a clinical scenario).

We have modified the introduction to add additional detail regarding the validity of the dynamical systems approach. Specifically, we now state:

Introduction, Paragraph 3 (Page 4, Line 81):

*Better understanding of propagation would benefit from the use of an appropriate model system that is relevant across a range of seizures. Epilepsy is a heterogenous disease, with many etiologies [9] and pathways to seizures, a concept called degeneracy [10]. Likewise, a particular seizure propagation pattern may emerge from a number of underlying pathologies. Research focused on a particular biological mechanism can provide important insights into a specific subset of epilepsies, but few findings generalize across a wider range of heterogeneity of the disease. Yet despite the biological diversity associated with the generation of epileptic seizures, the resulting dynamical mechanisms underlying seizure onset and termination seem to be more limited [11,12]. A complementary approach to epilepsy research is thus to focus on the invariant properties characterizing each dynamical mechanism [11]. One invariant property is how the brain transitions between resting state and seizure [11–13], with commonly proposed dynamical mechanisms including bifurcations and noise-induced transitions [12,14,15]. Work that assumes the former mechanism has shown that there are a limited number of bifurcations governing the transitions between resting and seizing states in human epilepsy (six total in a first approximation; four onset and four offset) [11,16,17]. These bifurcations can be inferred from their characteristic timeseries properties and are present across etiologies, brain regions, and species [11,16,18]. They are even observed in experimental conditions that prevent synaptic transmission [11]. Thus, while a focus on invariant properties necessarily sacrifices direct biological connection, the advantage of this approach is universality.*

We also added an additional paragraph to the discussion addressing this comment:

Discussion, Rationale for model development, Paragraph 1 (Page 28, Line 669):

*Seizures exhibit a high degree of degeneracy: there are many etiologies and mechanisms of seizure generation [9,10]. The degenerate nature of seizures forces a choice between biological detail and universality. Computational (or indeed, experimental) models tied to biological mechanisms are unable to reveal generic properties generalizable across epilepsies. Conversely, phenomenological models lack a direct connection to biology. We chose the latter approach, sacrificing biological detail to focus on universal emergent properties of epileptic brains. Note that the phenomenological approach does not negate the possibility of relating components of the model to specific biophysical processes. Indeed, state variables in the Epileptor, a phenomenological model for one seizure dynamotype, have been related to specific ion concentrations and glutamatergic and GABAergic activity in a low magnesium hippocampal preparation [11]. However, the model’s predictions held even when inhibiting neurotransmitter release, demonstrating that they are tied to the phenomenon of seizures, not the specific mechanisms. Likewise, the propagation model presented here constrains the dynamics of seizure propagation, but not the underlying biological mechanisms, which may vary in different experimental conditions or patients.*

Regarding the specific point about baseline shift, we refer to true baseline shifts produced by the tissue, not artifacts. With proper recording technique, baseline shifts have been documented in EEG at seizure onset in both animals and humans (e.g. Ikeda et al., Brain, 1999 in humans and Jirsa et al., Brain 2014 in mice). There are multiple studies showing they are specific to the seizure onset zone (e.g. Miller et al., NeuroImage, 2007; Kim et al., Journal of Clinical Neurophysiology, 2009) which confirms the baseline shift is a signal produced by the tissue. We have added a sentence clarifying this point into the methods, as indicated below.

Model and methods, Connecting seizure onset and propagation zones, Paragraph 1

(Page 10, Line 214):

*The Multiclass Epileptor describes the behavior of a cortical mass or “node”. Although the model describes seizure features that are present at different scales [11], in this work, it may be helpful to think of a node as the mass under a single SEEG electrode contact. The characteristic properties of bifurcations can be observed in the local field potential produced by this tissue.*

9. Lack of Statistical Rigor and Anecdotal Clinical Validation

The manuscript makes quantitative-sounding claims—such as certain dynamotypes being "less likely to spread" or spreading more slowly—but fails to support these assertions with statistical rigor. The clinical and real-life data section relies on anecdotal, cherry-picked examples of human intracranial EEG (iEEG) traces to support "unusual combinations" of seizure dynamics. Without systematically quantifying these occurrences across a sufficiently powered clinical dataset, the premise that the model reflects real-world seizure dynamics remains unsubstantiated. Claims regarding the probability of seizure spread must be backed by appropriate statistical testing.

We apologize for omitting statistical testing. As indicated below, we have added statistical support for our claims regarding recruitment proportion, delay, and energy. Note that, for results regarding recruitment proportion (Fig 5A), each datapoint now corresponds to the recruitment proportion for one node 2 location (calculated across all node 1 paths) as opposed to one node 1 path (calculated across all node 2 locations). This grouping increased the number of measurements from 6 to 71 so that we were better powered for statistical testing. Also, if we group recruitment by node 1 path, the measurements are not independent (measurements for dynamotypes with the same offset bifurcation are correlated, meaning we really have 8 groups of 3 as opposed to 4 groups of 6). Grouping by node 2 location results in better independence between measurements.

Model and methods, Recruitment analysis, Paragraph 2 (Page 14, Line 324):

*Statistical significance in recruitment properties between groups was evaluated via Kruskal-Wallis tests followed by multiple comparisons with Bonferroni correction, if appropriate.*

Results, Recruitment properties with respect to node 1 onset bifurcation, Paragraph 2-3

(Page 20, Line 469):

*Consistent with theoretical predictions, nodes in the LCb bistability region were more likely to recruit than nodes in the LCs bistability region. SN(-DC)- and subH-onset dynamotypes, located in LCb, showed elevated recruitment (Fig 5A) compared to SN(+DC)- and supH-onset dynamotypes, located in LCs. Recruitment proportion was particularly high for subH-onset dynamotypes, and particularly low for supH-onset dynamotypes.* ***All differences in recruitment proportion were statistically significant (Kruskal-Wallis test p = 2.5x10-184; post-hoc testing with Bonferroni correction yielded p < 0.001 for all pairwise comparisons).*** *Thus, the model predicts that seizures that do not start with baseline shifts are more likely to spread than seizures that display baseline shifts at onset. Seizures with amplitude scaling at onset are unlikely to spread.*

*SN(+DC)-onset node 1 dynamotypes also demonstrated longer recruitment delays (Fig 5B) compared to all other dynamotypes* ***(Kruskal-Wallis test p < 2.2x10-308; p < 1x10-190 for all pairwise comparisons with Bonferroni correction including SN(+DC)-onset dynamotypes).*** *Hence, the model predicts that seizures that exhibit baseline shifts at onset spread more slowly than seizures that do not exhibit baseline shifts at onset. Recruitment energy was highest for SN(+DC)-onset node 1 dynamotypes, followed by SN(-DC)-onset dynamotypes, then subH-onset dynamotypes, and finally lowest for supH-onset dynamotypes (Fig 5C).* ***All differences were statistically significant (Kruskal-Wallis test p < 2.2x10-308; p < 1x10-31 for all pairwise comparisons with Bonferroni correction).***

Results, Recruitment properties with respect to node 2 initial location, Paragraph 1

(Page 22, Line 527):

***The initial location of node 2 in parameter space also significantly influenced recruitment properties (Kruskal-Wallis tests yielded p<6.01x10-7 for comparisons of recruitment proportion, delay, and energy across node 2 initial locations).*** *For most node 2 initial locations, recruitment proportion was consistently low (Fig 7). Recruitment proportions increased and more node 2 initial locations were recruited at high excitability and coupling strength values (S2 Fig). Generally, recruitment delays and energy were consistent and decreased moving from near the LCs region to near the LCb region (Fig 7). These results are consistent with recruitment via mechanism A. Recall that in mechanism A, node 2 reaches node 1 in parameter space before node 1 leaves its onset bifurcation. Thus, recruitment through mechanism A is likely to be low and increase with node 1 excitability and coupling strength. Further, recruitment delay and energy are stereotyped because node 2 can only be recruited in a narrow timespan.*

We agree that the comparisons are proof-of-concept, and there are two reasons for this. First, it is critical to point out that these data are presented from a clinical viewpoint. Some of the examples are patterns seen commonly in human intracranial EEG, which are presented to show how such patterns can be explained via the lens of dynamotypes, i.e. why does the recruited node have different dynamics than the onset node, and how can we describe it? But more importantly, other examples are highly unusual patterns that clinicians might consider impossible, such as a DC shift in the recruited node but not the initial node. Indeed, our clinician author (WS) thought such a prediction impossible, and challenged the first author (DK) to find examples to show that such a situation was even plausible. Thus, while such examples may appear anecdotal, they serve a critical purpose: when disproving the hypothesis that something is impossible (or proving a high-risk hypothesis that something is plausible), a few examples are sufficient to establish plausibility. We found multiple examples even in our limited dataset, showing that these situations were uncommon but certainly found in nature. Thus, the examples serve two purposes: as visual demonstrations to show how our modeled data could translate to human EEG, and as proof that some of our unusual predictions can be found in nature.

Second, while we are certainly interested in applying these predictions to human data, it is also critical to point out the scope that would be necessary to prove these predictions rigorously. This will be a massive project far outside the scope of this initial study. We estimate needing thousands of seizures from intracranial EEG, each with the onset time and dynamotype marked on every channel. Thousands of seizures are necessary because many of the dynamotypes are much less common in humans, as we showed in our prior work (Saggio et al., eLife, 2020). Then, these studies usually have >100 channels, all of which must be categorized for onset time and type as well. Currently, this can only be done via visual analysis by a trained rater. In addition, the volume of tissue corresponding to each electrode/group of electrodes would need to be estimated, as well as the distance between every electrode pair. To be rigorous, this must account for the sylvian fissure and the intrahemispheric fissure at least, and potentially for white matter tracts. Further, the number of electrodes and electrode locations vary between patients; hence, a method of normalizing propagation analysis across patients would need to be developed. This will require literally thousands of hours of work. We are developing automated tools to speed up portions of this work, but they also must be validated on many hundreds of seizures that are similarly marked. These endeavors must be justified by computational modeling. The current paper motivates this validation work in the future.

We clarified the intention of our comparisons to human data with the following addition to the discussion:

Discussion, Model limitations, Paragraph 4 (Page 34, Line 812):

*These examples are not meant to be a robust validation of the predictions; rather, they are a demonstration that the recruitment behaviors predicted using our propagation model, several of which were unusual and unexpected, were plausible and could be found even in our dataset of under 200 patients. Robust validation of all model predictions would require labeling exact onset times and compatible bifurcations in every channel (up to hundreds of channels) for tens to hundreds of seizures for every dynamotype in large human EEG datasets, a task far beyond the scope of this initial study. Our results motivate that future work, as well as the development of automated multi-channel dynamotype classification methods to enable such an undertaking.*

However, we also appreciate this reviewer’s concern that additional validation would be helpful to assess the plausibility of our results. As an initial analysis, we tested whether seizures with DC shift propagate differently than those with arbitrary amplitude in the seizure onset zone. In preparing the data, we realized how complicated this comparison would be given the heterogeneous nature of patients with epilepsy. We thus restricted our analysis to temporal lobe epilepsy when searching our database of patients undergoing intracranial EEG for potential epilepsy surgery. We selected 20 consecutive patients with temporal lobe epilepsy that demonstrated seizure onset patterns that were unambiguously consistent with either a SN(+DC) or SN(-DC)/SubH bifurcation. We did not consider seizures with amplitude scaling at onset (consistent with a supH bifurcation) for this initial analysis since these are both relatively rare in our dataset and more difficult to unambiguously identify via visual analysis. We analyzed one seizure per patient.

For the analysis, we identified whether there was a baseline shift at the onset of the seizure, as well as the number of iEEG channels involved in the seizure. Whether a channel was involved in the seizure was determined by our clinical expert (WS). We found 11 seizures with baseline shifts and 9 without. We found there to be significantly more channels involved in seizures that did not have baseline shifts at onset, as predicted by the model. Both the number of propagation channels (p = 0.00921) and the proportion of channels the seizure propagated to (p = 0.02) were significantly higher for seizures without baseline shifts at onset at a significance level of 0.05 (one-sided Wilcoxon rank sum tests). The number of channels was not significantly different between groups (p = 0.224; two-sided Wilcoxon rank sum test). Results are shown below.

However, in doing this analysis we realized just how difficult model validation will be. There is tremendous variability between patients: the electrode location and number is different, the pathology is different, the seizures are different even in the same patient. Medications are changing. The speed of propagation would have to be measured using 3D coordinates of the electrodes, and it is unclear what pathway to use. Thus, we must strongly stress that the analysis above is NOT a robust validation: it does not consider nearly enough seizures, electrode locations and configurations were not standardized across patients, and we did not identify amplitude scaling at onset since this is less visually obvious. **For these reasons, while we provide it to this reviewer as a demonstration of plausibility, we will not include this figure in the manuscript.** Such future work will require vastly larger amounts of data and analysis, as described above.

Finally, we would like to point out that PLoS Computational Biology regularly publishes modeling papers without clinical or experimental data, including one using the Epileptor. Some examples are listed below. We actually consider our inclusion of multiple examples from human patients to be a strength of this submission.

- Danilczuk M, Pokropski M, Suffczynski P (2026) The integrated information Φ of an integrate and fire network. PLoS Comput Biol 22(3): e1014085. https://doi.org/10.1371/journal.pcbi.1014085
- Singh P, Sheen J, Saad-Roy CM, Levy MZ, Metcalf CJE (2026) Coevolution of host resistance and pathogen exploitation in a propagule-mediated infection model. PLoS Comput Biol 22(3): e1013999. https://doi.org/10.1371/journal.pcbi.1013999
- Vallet A, Blanco S, Chevallier C, Eustache F, Gautrais J, Grandpeix J-Y, et al. (2026) Sensitivity analysis enlightens effects of connectivity in a Neural Mass Model under Control-Target mode. PLoS Comput Biol 22(3): e1014035. https://doi.org/10.1371/journal.pcbi.1014035
- Saggio ML, Jirsa V (2024) Bifurcations and bursting in the Epileptor. PLoS Comput Biol 20(3): e1011903. https://doi.org/10.1371/journal.pcbi.1011903
- Beiran M, Ostojic S (2019) Contrasting the effects of adaptation and synaptic filtering on the timescales of dynamics in recurrent networks. PLoS Comput Biol 15(3): e1006893. https://doi.org/10.1371/journal.pcbi.1006893
- Lade SJ, Gross T (2012) Early Warning Signals for Critical Transitions: A Generalized Modeling Approach. PLoS Comput Biol 8(2): e1002360. <https://doi.org/10.1371/journal.pcbi.1002360>

10. Omission of Anatomical Constraints in Propagation

In evaluating the iEEG data, the authors fail to acknowledge the profound role that brain anatomy and structural connectivity play in seizure propagation at the cellular and network level. Seizure spread in the human brain is not merely a function of abstract node-to-node dynamical coupling; it is heavily constrained by white matter tracts, spatial proximity, local circuitry, and network topology. Indeed if the authors could link dynamotypes to anatomical locations and their onset and spread pattern in clinical data, that would form a far more convincing paper.

We thank the reviewer for bringing up the role of anatomy and structural connectivity in seizure propagation. Our toy model was designed to investigate the impact of seizure dynamics on propagation, not to recreate specific seizure networks. Our toy model enables us to identify some general rules regarding seizure propagation; for example, that subH-onset dynamotypes demonstrate elevated recruitment. In demonstrating that seizure dynamotype impacts propagation, we motivate the inclusion of richer dynamical information in future patient-specific modeling. However, the nuances of recruitment in a specific patient will of course also be affected by anatomical and functional aspects of the seizure network. As mentioned by reviewer 1, the spatial behavior of propagation is being modeled in advanced patient-specific models such as TVB, but that is so complex that they sacrifice dynamical diversity, instead modeling each brain region using the same dynamical model for one seizure dynamotype. It requires supercomputers to render each patient, and even then there are significant assumptions that must be made. For this initial analysis of how dynamotypes affect propagation, we ignore the extra constraint of anatomical constraints. We newly acknowledge this limitation in the discussion:

Discussion, Model limitations, Paragraph 2 (Page 32, Line 774):

*To focus on the dynamical conditions under which a seizure onset zone is more likely to recruit a propagation zone, we implemented a toy model of seizure propagation. While the use of such a toy model facilitated the study of dynamical factors and recruitment in isolation, it is a simplification of clinical seizure propagation…* ***Further, biological (e.g. anatomy, structural connectivity) and dynamical factors will interact to produce a patient’s specific propagation patterns. For example, we found that, under sufficiently high coupling strength, SN(+DC)-onset dynamotypes can demonstrate recruitment proportions on par with SN(-DC)- and subH-onset dynamotypes (Fig 6). High coupling strength could correspond to increased structural or functional connectivity, or other physiological processes that increase the relative influence of the seizure onset zone on a brain region. Our use of a toy model is not meant to imply patient-specific anatomy and physiology is unimportant, but rather to motivate the inclusion of dynamical conditions in addition to these biological ones in future studies.***

11. Target Audience and Theoretical Contribution

The paper struggles to find its audience. For experts in dynamical systems, the insights into coupled oscillators and bifurcations may appear trivial or derivative of existing nonlinear dynamics literature. Conversely, for the non-expert (e.g., clinical epileptologists or systems neuroscientists), the dense mathematical focus and lack of biological translation make the paper impenetrable. The authors need to clearly articulate the novel biological insight generated by this mathematical exercise.

We hope to appeal to audience members with both mathematical and biological backgrounds. We thank the reviewer for identifying the requirements to make this paper more accessible to non-mathematical audience members. We have moved some of the mathematical aspects of the paper to the Supporting Information (particularly S1 Supplementary Methods and S1 Fig) to make the paper more approachable. We have also expanded our discussion section ‘Clinical relevance’ to further highlight the utility of our findings for a non-mathematical audience.

We do not agree that experts in dynamical systems would find our result trivial. Among dynamical systems models of neural activity, the Multiclass Epileptor uniquely describes the relationships between multiple bifurcations via the parameter space map. By establishing, for the first time, a coupling method in the Multiclass Epileptor, we are able to study recruitment in this rich dynamical space. This is a unique aspect of our propagation model, which we now emphasize in the discussion section ‘Rationale for model development’. Specifically, we state:

Discussion, Rationale for model development, Paragraph 2 (Page 29, Line 685):

*To investigate how a rich array of seizure onset and propagation zone dynamics influence recruitment, we introduced a novel method to connect two nodes in the Multiclass Epileptor. To our knowledge, the Multiclass Epileptor is the only model in the literature that can reproduce a variety of dynamotypes (i.e. a range of onset and offset bifurcations) seen in human seizures in an easily controllable way. The Multiclass Epileptor describes the relationships between multiple bifurcations via the parameter space map; by establishing a coupling method in the Multiclass Epileptor, we enabled the first study of recruitment in this rich dynamical space. While different onset/offset bifurcations can also be obtained by tuning the parameters of well-known models such as the Jansen-Rit [36] and its extension, the Wendling-Chauvel [37], these models typically lack slow dynamics for autonomous seizure generation. Further, previous dynamical models of epilepsy networks have focused on single dynamotypes and generally require bistability or excitability [19–21,23,24,32,38,39]. Thus, a unique aspect of our study is the simulation of a wide range of dynamics in the seizure onset and propagation zones.*

12. Manuscript Structure, Length, and Clarity

The manuscript is excessively long and lacks a cohesive narrative arc.

Obscured Main Results: The primary findings are difficult to locate amidst dense, poorly structured sections.

Figure Clarity: The figures and their captions are overly abstract. They do not guide the reader toward a cohesive story or clearly illustrate the biological equivalents of the mathematical phenomena.

Need for Streamlining: Entire sections detailing the mathematical derivations or exhaustive parameter sweeps should be moved to the Supplementary Material to allow the main text to focus on the core biological hypotheses and results.

We thank the reviewer for their suggestions. We have moved parts of several sections to the supporting materials, along with some figures. Namely:

- The following sections of the methods have been moved in part or in full to S1 Supplementary Methods:
  - Description of the Multiclass Epileptor
  - Connecting seizure onset and propagation zones
  - Simulation parameters
  - Identification of recruitment
  - Identification of burst onset and offset
- The following sections of the results have been moved in part or in full to S2 Supplementary Results:
  - Results analyzing the influence of parameter space geometry
  - Results for SN followed by SupH-onset dynamotypes
  - Results analyzing the influence of the coupling term
  - Model predictions and clinical examples of propagation zone dynamics
- Figures moved in full or in part to the supplementary materials: 3, 6, 11, 12, 16

We have also restructured the introduction, parts of the results, and the discussion. We hope that these modifications improve the clarity and cohesion of the paper.

**Reviewer #3:**

13. It is unclear to me why the Authors focus on hysteresis loops for onset and offset of the seizure dynamics. Why do they exclude the case of moving in and out of the ictal only regime from active rest or rest, i.e. the slow wave mechanism they mention. The model is phenomenological, therefore placing restrictions such as generation of "autonomous" seizures seems arbitrary. Could the Authors explain in further detail why some autonomous mechanism cannot be achieved in the slow wave scenario, for example by implementing an autonomous control mechanism using the amplitude of the simulated signal?

We thank the reviewer for raising this question. The model being phenomenological doesn’t imply it is unconstrained, rather it is constrained by the dynamics we can hypothesize based on data. The statistics of seizure duration (Suffczynski et al., IEEE Transactions on Biomedical Engineering, 2006.) point to a deterministic process governing seizure termination. In addition, many processes contributing to seizure termination are triggered by the abnormal fast activity itself (Lado & Moshe, Epilepsia, 2008), so that a feedback mechanism between fast and slow variables plays an important role. Both constraints are satisfied by the classic hysteresis-loop formulation (Izhikevich, MIT Press, 2007). Not only is the role of feedback not accounted for by the slow-wave mechanism by definition (Izhikevich, MIT Press, 2007), but this implies that the slow-wave formulation requires a very careful tuning of the path in parameter space because slow-wave paths can’t autonomously adjust to the ongoing activity. There are thus two reasons why we limited the analysis to hysteresis-loop: autonomy and robustness of seizure termination. We made it more clear in the text what are our dynamical constraints and why hysteresis-loop is the natural choice given classic works on bursting dynamics (see for example Izhikevich, MIT Press, 2007), and in line with the choices made in the Epileptor work (Jirsa et al., Brain, 2014). We do not have an answer on how to build a robust autonomous burster in the absence of bistability. If possible, the control mechanism could not rely only on the current amplitude because this cannot distinguish whether the system is moving towards or away the bifurcation. Maybe this could be possible with more complex feedback including some form of memory.

We have added a segment to S1 Supplementary Methods to emphasize the differences between hysteresis and slow-wave implementations. The relevant paragraph now reads:

S1 Supplementary Methods Page 2, Paragraph 2:

*Multiple realizations of the slow subsystem are possible [1,3,4]. In particular, hysteresis-loop implementations exploit bistability in the fast subsystem and feedback among fast and slow variables to produce autonomous bursting [12]. These paths capture the endogenous mechanisms that move the brain towards seizure termination upon onset, which occurs in all cases except status epilepticus [2]. The statistics of seizure termination support an underlying deterministic process [13]. In contrast, slow-wave implementations lack this feedback, may occur without bistability, and require slow variables to change independently to force the fast ones into bursting. The complexity of slow-wave implementations is also higher than that of hysteresis-loop implementations, requiring a minimum of two slow variables instead of one. For the present work, we chose hysteresis-loop paths for the node representing the active seizure focus (node 1), enabling node 1 to start and end a seizure autonomously. This is the simplest choice that enables seizures that progress towards termination through feedback from the fast activity. A single implementation was chosen for comparison among bursting paths.*

14. In terms of onset and offset of seizures, why would we assume that changes in parameter values are restricted to an arc between onset and offset points? Is there a biological reason for this, or could more complicated or "wandering" paths occur? This could affect the conclusions that are drawn, e.g. about the relationship between seizure onset dynamics and propagation dynamics.

The reviewer is correct that, in general, restricting the exploration of a three-dimensional space to a spherical surface is unjustified. However, this parameter space has a certain structure that guarantees that the same topology of bifurcation structure is found on any sphere with small enough radius (R<0.5), so that the topology on any of these spheres is informative of the entire repertoire around the origin. (In technical terms, this is due to the origin being a codimension 3 singularity, and its unfolding, that is our parameter space, having *cone structure* up to R=0.5; see Saggio et al., Journal of Mathematical Neuroscience, 2017)

We clarified the reasons behind the choice of restricting our exploration to a spherical surface in S1 Supplementary Methods:

S1 Supplementary Methods, Page 1, Paragraph 3 (after Eqn. 4):

*The state variable x is the output of the system, and tracks voltage content during a seizure, the equivalent of an EEG signal. Within the (x, y) state space of the model, resting states are represented by stable fixed points, and ictal states are represented by stable limit cycles. The existence of stable fixed points and limit cycles and therefore the behavior of the fast subsystem vary depending on the values of its parameters.* ***Because of the properties of this three-dimensional space, the relevant behaviors for this work can be captured by constraining the parameters of the fast subsystem to the surface of a sufficiently small sphere centered at the origin [5]. Note that the choice of parameter space geometry is arbitrary; only the relationships between parameter space regions with different dynamical repertoires are relevant, as captured, for example, using the spherical surface. Thus, as in previous work [1,4,6,7], we constrain the parameters of the fast subsystem to the surface of a sphere centered at the origin with radius 0.4 (Fig 1).*** *This reduces the dimensionality of the parameter space to two (the angular coordinates theta and phi).*

We also added the following to the main text:

Model and methods, The Multiclass Epileptor, Paragraph 4 (Page 7, Line 164)

*Note that the choice of parameter space geometry is arbitrary; only the relationships between regions on the sphere are relevant. It can be demonstrated for this model that the spherical surface captures these relationships well (see S1 Supplementary Methods).*

Once on the spherical surface, all paths traversing the same sequence of regions in parameter space are equivalent. We have previously shown (Saggio et al 2017) that for hysteresis-loop bursters in this space, arcs of great circle are enough to represent all the possibilities. We clarified this in the supporting information:

S1 Supplementary Methods, Page 2, Paragraph 4 (after Eqn. 5):

*A necessary condition for Eqn. 5 is that the bursting path exists in a bistable region of parameter space. We chose the bursting paths to be arcs of great circles that connect onset and offset bifurcations [1].* *As mentioned above for the choice of the spherical surface, the validity of the model is only in terms of the relationships between parameter space regions (how they are placed with respect one to the other) and not in terms of their geometry (such as size and precise shape). Thus, paths that traverse the same sequence of regions are equivalent in this framework and we can choose the simplest one without loss of generality [1]. Details of the implementation method can be found in [1].*

Another possibility is to move at different radii of the sphere or not restrict the movement to the surface of the sphere. As the parameters “under the spherical surface” have the same topology—i.e. pulling node 2 along a chord through the sphere’s interior will generate the same dynamical properties as pulling it along the arc surface. However, at larger radii the topology becomes much more complex, and bursting types may not always be achieved with arc paths (see Fig. 15 from Saggio et al 2017, which we include below for reference). We leave the study of these bigger parameter values for future work.

Finally, with regards to the movement of node 2 towards node 1, we chose the most direct route (an arc path) to node 1 as a first approximation. It is possible that more complicated or wandering paths could occur. Differences are expected only if the path traverses a different sequence of regions, and there are some sequences we do not obtain with the direct route. However, at the moment, there is no clinical or experimental data to support or constrain the implementation of more complicated paths. Hence, for this first exploration we focus on the simplest path, which is an arc. Notably, we already see all possible combinations of onset behavior (amplitude scaling, arbitrary amplitude, changes in baseline) using the arc path method. We added a few sentences to the discussion to acknowledge this, as shown below.

Discussion, Rationale for model development, Paragraph 4 (Page 30, Line 721):

*The novelty of our implementation is that, during coupling, the dynamics (parameter values) of the potential propagation zone are altered towards that of the onset zone. For this first exploration of recruitment among nodes with diverse dynamics, we moved node 2 along an arc path to node 1. This is the simplest method, but more complicated routes may be possible, where differences are expected only if the route traverses a different sequence of regions.*

From Saggio et al., Journal of Mathematical Neuroscience, 2017, DOI: 10.1186/s13408-017-0050-8

15. Similarly, equation 9 prescribes a specific form for the speed of movement along the arc. Looking at the supplemental video, it seems that G is doing most of the work here, since attraction of node 2 to the position of node 1 is almost instantaneous. What are the biophysical motivations for the choice of equation 9? Why would the biophysical mechanisms of recruitment unfold in this way? This affects some of the conclusions that are drawn, e.g. in the description of mechanism A, it is unclear why recruitment should occur early in the burst in node 1. This would simply depend on the dwell time in each part of the path of node 1 from onset to offset. The Authors state that the form of coupling captures the influence of the change in voltage in the seizure onset zone on the activity of the propagation zone. Could they please clarify what this influence is, biophysically?

16. In motivating the form of coupling chosen the authors argue that the propagation zone often exhibits the same onset patterns as the seizure onset zone. This could be achieved in the model by implementing some form of synaptic driving: e.g. the node 2 is in a rest state and simply responds to the oscillations of node 1. This would not always lead to identical dynamics in both nodes, due to the dynamics of the response in node 2 and any delays etc. Therefore I do not follow the logic in the passage beginning "First, the highly excitable onset zone… ". Node 2 could be in any region of resting dynamics for this to occur (i.e. wouldn't need to be in the same part of this space as node 2).

We thank the reviewer for highlighting the need to justify our choice of coupling method and function with comments 15 and 16. The Multiclass Epileptor is not designed to capture a specific biophysical mechanism, but rather to model invariant properties that can result from many different biophysical mechanisms. Likewise, our propagation model is not meant to recreate a specific biophysical mechanism of recruitment (e.g. synaptic driving), but rather to constrain the dynamics that can result from the aggregate influence of biophysical mechanisms. While the reviewer gives very likely scenarios that we agree with, they are not the only scenarios that are possible. Our model is designed to be independent of specific physiological mechanisms.

However, we can explain the motivation behind these choices: in general, the coupling should be capable of reproducing what happens in human intracranial EEG recordings. Specifically, it should be able to reconcile the following conditions that we see clinically:

1. We see clinically that a clear seizure focus can start firing, and can recruit the entire brain on both sides in less than a second, even regions that never produce any epileptic activity. This suggests that the coupling mechanism should be able to rapidly recruit from every region quickly.

2. We see that seizures can jump across fissures, spread slowly in an increasing volume as a “spreading field”, and we had previously shown they can occur without synaptic transmission (Jirsa et al Brain 2014). This suggests it should not depend necessarily on synaptic transmission. While we expect synaptic transmission to be the major player, it cannot be the only mechanism.

3. While it is common for the recruited brain to adopt the same dynamics as the seizure focus, this is not required. There are examples when ‘node 2’ adopts completely independent dynamics, not simply a filtered response from ‘node 1’. Node 2 can have a different dynamotype completely, can continue firing after node 1 is done, etc. This also argues strongly that node 2 should not simply be driven by node 1.

4. One historical method for epilepsy surgery was subpial transections, in which the U fibers connecting neighboring cortex are cut to abolish synaptic transmission. This sometimes works, but sometimes does not. When it fails, nonsynaptic mechanisms (e.g. ephaptic transmission) are highly likely.

5. In many cases the surgical removal of the seizure focus completely abolishes all seizures. Although we cannot comment on mechanisms, we can heuristically state confidently that this condition appears to be a “node 1” that resides in a region prone to seizures, and “node(s) 2” that reside in a region very far from seizures in parameter space.

6. Time in our model is arbitrary, as is the distance and speed by which the nodes traverse the parameter space. Thus, we have been careful to assure our results do not depend on specific “time” and make sure the comparisons are normalized, i.e. can node 1 make a ‘distant’ node 2 seize nearly immediately?

In general, we envision three primary methods of transmission: synaptic, ephaptic, and gap junctions. But we do not ascribe any particular mechanism—like the bursting, we designed the coupling to capture invariant properties rather than try to fit specific physiology. Nevertheless, all of these can be modeled as node 2 receiving some transformation of the extracellular voltage in node 1, so it is physiologically plausible. Of note, the last author has modeled each of those effects in similar fashion; the primary difference is that synaptic transmission has a short (< 1 second) delay which is arbitrary in the current paper and thus not necessary. The general form in this paper was inspired by those prior works (<https://www.ncbi.nlm.nih.gov/pubmed/21273309>, <https://www.ncbi.nlm.nih.gov/pubmed/19657077>

<https://www.ncbi.nlm.nih.gov/pubmed/12424297>

<https://www.ncbi.nlm.nih.gov/pubmed/26146658>)

We would like to address our choice of dynamical mechanism for coupling as well. The form of synaptic driving the reviewer mentioned would be fast-to-fast coupling. We implemented fast-to-slow coupling. If the reviewer is interested in the comparison between these coupling types, please see the response to comment #3.

Combining all of these observations, we developed the coupling mechanism in this work. It is flexible enough to satisfy all of these points, yet simple enough to avoid overfitting. We have added paragraphs to the introduction and to the discussion to clarify the phenomenological nature of the Multiclass Epileptor. In the introduction, we state:

Introduction, Paragraph 3 (Page 4, Line 81):

*Better understanding of propagation would benefit from the use of an appropriate model system that is relevant across a range of seizures. Epilepsy is a heterogenous disease, with many etiologies [9] and pathways to seizures, a concept called degeneracy [10]. Likewise, a particular seizure propagation pattern may emerge from a number of underlying pathologies. Research focused on a particular biological mechanism can provide important insights into a specific subset of epilepsies, but few findings generalize across a wider range of heterogeneity of the disease. Yet despite the biological diversity associated with the generation of epileptic seizures, the resulting dynamical mechanisms underlying seizure onset and termination seem to be more limited [11,12]. A complementary approach to epilepsy research is thus to focus on the invariant properties characterizing each dynamical mechanism [11]. One invariant property is how the brain transitions between resting state and seizure [11–13], with commonly proposed dynamical mechanisms including bifurcations and noise-induced transitions [12,14,15]. Work that assumes the former mechanism has shown that there are a limited number of bifurcations governing the transitions between resting and seizing states in human epilepsy (six total in a first approximation; four onset and four offset) [11,16,17]. These bifurcations can be inferred from their characteristic timeseries properties and are present across etiologies, brain regions, and species [11,16,18]. They are even observed in experimental conditions that prevent synaptic transmission [11]. Thus, while a focus on invariant properties necessarily sacrifices direct biological connection, the advantage of this approach is universality.*

In the discussion, we state:

Discussion, Rationale for model development, Paragraph 1 (Page 28, Line 669):

*Seizures exhibit a high degree of degeneracy: there are many etiologies and mechanisms of seizure generation [9,10]. The degenerate nature of seizures forces a choice between biological detail and universality. Computational (or indeed, experimental) models tied to biological mechanisms are unable to reveal generic properties generalizable across epilepsies. Conversely, phenomenological models lack a direct connection to biology. We chose the latter approach, sacrificing biological detail to focus on universal emergent properties of epileptic brains. Note that the phenomenological approach does not negate the possibility of relating components of the model to specific biophysical processes. Indeed, state variables in the Epileptor, a phenomenological model for one seizure dynamotype, have been related to specific ion concentrations and glutamatergic and GABAergic activity in a low magnesium hippocampal preparation [11]. However, the model’s predictions held even when inhibiting neurotransmitter release, demonstrating that they are tied to the phenomenon of seizures, not the specific mechanisms. Likewise, the propagation model presented here constrains the dynamics of seizure propagation, but not the underlying biological mechanisms, which may vary in different experimental conditions or patients.*

In developing our coupling function, we reasoned that the emergent property of many biophysical mechanisms is that the novel activity in the seizure onset zone (the change in voltage: x_1_ – x_1, rest_) influences the propagation zone. We squared this term to avoid biasing recruitment towards bursting paths in the ‘limit cycle small’ region. We added a paragraph to the discussion justifying our choice of coupling function, while also acknowledging that other choices may be equally valid:

Discussion, Rationale for model development, Paragraph 4 (Page 30, Line 721):

*The novelty of our implementation is that, during coupling, the dynamics (parameter values) of the potential propagation zone are altered towards that of the onset zone. For this first exploration of recruitment among nodes with diverse dynamics, we moved node 2 along an arc path to node 1. This is the simplest method, but more complicated routes may be possible. As a first approximation, we chose the speed of movement of node 2 towards node 1 to be proportional to the square of the difference from rest in node 1’s x variable (Eqn. 3). In developing this coupling function, we reasoned that the novel activity in the seizure onset zone (i.e. the electric field generated, represented by the change in voltage: x1 – x1, rest) influences the propagation zone. The coupling is phenomenological and thus effectively summarizes the cumulative effect of a variety of processes which depend on the specific conditions and locations of the brain regions involved. We squared the change in voltage to avoid biasing recruitment towards bursting paths in LCs (which have a distinct separation between bursting and resting states; see [13]) compared to bursting paths in LCb (in which oscillations cross the resting state). To further ensure that the coupling function was not biasing results, we calculated the area under the entire coupling term G(x1 – x1, rest)2 during the period between node 1 and node 2 onset. We called this value the recruitment energy. If the coupling function biased recruitment towards certain dynamotypes, we would expect dynamotypes with elevated recruitment proportions to also demonstrate elevated recruitment energy. However, there was no consistent relationship between recruitment proportion and recruitment energy among node 1 onset bifurcations (Fig 5). We therefore infer that the differences in recruitment proportion are not due to biases or saturation effects introduced by the choice of coupling term. We also found no major difference in results with a different coupling function (S2 Supplementary Results). Nevertheless, other choices for the coupling term could be equally relevant and are an area for future study.*

Regarding this specific point: “in the description of mechanism A, it is unclear why recruitment should occur early in the burst in node 1,” please see comment 20.

17. As an aside, I am unsure how much relevance to place on the size of these different regions since the model is phenomenological: we have no reference point for which regions of the parameter space it could or could not inhabit, nor the relative sizes of these. In this same section the authors state ".. seizures often propagate to brain regions that are not independently epileptogenic". How do we know this is the case? How could we verify that a propagation zone is not inherently epileptogenic? Presumably by looking into post-surgical seizure freedom over a sufficient time scale?

We thank the reviewer for their comments. We agree that the shape of the parameter sphere is arbitrary, so the size of the different regions in parameter space (i.e. the geometry) is not relevant. Our choice of geometry was for consistency with prior work. However, the important aspect is the relative relationships between/positions of the bifurcations and regions; those are mathematically precise. We have clarified the reasons behind the choice of restricting our exploration to a spherical surface in S1 Supplementary Methods:

S1 Supplementary Methods, Page 1, Paragraph 3 (after Eqn. 4):

*The state variable x is the output of the system, and tracks voltage content during a seizure, the equivalent of an EEG signal. Within the (x, y) state space of the model, resting states are represented by stable fixed points, and ictal states are represented by stable limit cycles. The existence of stable fixed points and limit cycles and therefore the behavior of the fast subsystem vary depending on the values of its parameters. Because of the properties of this three-dimensional space, the relevant behaviors for this work can be captured by constraining the parameters of the fast subsystem to the surface of a sufficiently small sphere centered at the origin [5]. Note that the choice of parameter space geometry is arbitrary; only the relationships between parameter space regions with different dynamical repertoires are relevant, as captured, for example, using the spherical surface. Thus, as in previous work [1,4,6,7], we constrain the parameters of the fast subsystem to the surface of a sphere centered at the origin with radius 0.4 (Fig 1). This reduces the dimensionality of the parameter space to two (the angular coordinates theta and phi).*

We also added the following to the main text:

Model and methods, The Multiclass Epileptor, Paragraph 4 (Page 7, Line 164):

*Note that the choice of parameter space geometry is arbitrary; only the relationships between regions on the sphere are relevant. It can be demonstrated for this model that the spherical surface captures these relationships well (see S1 Supplementary Methods).*

We previously analyzed the influence of distance on bifurcation properties (now in S2 Supplementary Results) to ensure the parameter space geometry was not biasing results, specifically because of this concern.

It is true that we do not yet have methods for mapping brain regions in the resting state to parameter space locations. For brain regions at rest, we only know that they cannot inhabit the ‘ictal-only’ region. Hence, we explored all possibilities. Future investigations on clinical data will restrict the possibilities.

From our (WS) clinical experience, we know that patients with focal seizures that generalize can achieve seizure freedom after resective surgery of the onset zone. Hence, we conclude that non-resected propagation zones are not inherently epileptogenic, meaning they do not seize in the absence of coupling to the seizure onset zone. We added a sentence to highlight this justification:

Model and methods, Connecting seizure onset and propagation zones, Paragraph 2 (Page 10, Line 222):

*However, the propagation zone does not always adopt the same dynamics as the seizure onset zone, and seizures often propagate to brain tissue that is not independently epileptic.* ***The latter point is evidenced by seizure freedom after resective surgery of the seizure onset zone in patients that experience seizures with focal onset followed by generalization.***

18. In terms of the theoretical prediction: I'm not sure what "more likely to recruit" means: each node 2 will follow its path through to the spiking dynamics, given sufficient coupling and speed. Also, that nodes in active rest are more likely to be recruited than nodes in rest. I guess there is an implicit assumption here that the relative size of the parameter space is meaningful. It would be helpful if these assumptions were spelled out and justified.

We thank the reviewer for pointing out a lack of clarity in our use of “more likely to recruit.” Nodes that are more likely to recruit will tend to recruit more node 2s at some fixed coupling strength G. We have modified the relevant sentence:

Results, Theoretical predictions, Paragraph 1 (Page 17, Line 404):

*First, under equal coupling strength, nodes in the LCb bistability region will recruit node 2s from more initial locations than nodes in the LCs bistability region.*

It is true that increasing the coupling strength G will eventually guarantee recruitment for SN- and subH-onset node 1 dynamotypes. However, some node 1 dynamotypes can recruit node 2s with lower G than others. For supH-onset node 1 dynamotypes, the onset bifurcation is not embedded in the ‘ictal-only’ region. As such, even infinitely high G (such that node 2 instantaneously reaches node 1) is not sufficient for recruitment.

Similarly, by “nodes in active rest are more likely to be recruited that nodes in rest,” we refer to a tendency toward increased recruitment for any given combination of coupling strength and node 1 dynamotype. We have modified the relevant sentence:

Results, Theoretical predictions, Paragraph 2 (Page 18, Line 414):

*Second, nodes in active rest are more likely to be recruited than nodes in rest for a given combination of node 1 dynamotype and coupling strength.*

This is not due to the relative size of the parameter space, but rather the location of active rest relative to the ictal-only region and the state space structure of active rest compared to rest. Fig 4 and S1 Fig illustrate these arguments.

We further address the issue of size in comments 14, 17.

19. "The variability of ictal inter-spike intervals across bursting paths was confirmed to be within the expected physiological range": Since the time scale in the model is arbitrary, what is the reference point for this?

We compared the inter-spike intervals (in arbitrary units) to each other. Based on our (WS) clinical expertise, we expect seizures to involve spiking at frequencies between 2 Hz and 30 Hz, which is an order-of-magnitude range. Inter-spike intervals among the bursting paths were also within an order-of-magnitude range.

20. "Given that mechanism B does not rely as heavily on node 2's speed".. I'm not sure I follow: speed is still important since node 2 has to be moved across bifurcations.

We thank the reviewer for pointing out our lack of clarity. To clarify this point, we have updated our description of mechanism A and B:

Results, Possible mechanisms of recruitment, Paragraph 2 (Page 16, Line 370):

*In mechanism A, node 2 does not cross onset bifurcations before reaching node 1 and reaches node 1 before node 1 has left the proximity of its onset bifurcation. Node 2 can be recruited if node 1’s onset bifurcation is SN or subH since these bifurcations are embedded in the ictal-only region. Crossing these onset bifurcations destabilizes the stable fixed point (i.e. resting state) for node 2, leading to recruitment. Recruitment through mechanism A must occur early in the burst in node 1. If node 2 reaches node 1 after node 1 leaves its onset bifurcation, then node 2’s resting state will not be destabilized, and node 2 will follow node 1 in parameter space without leaving the stable fixed point in state space. Even if the two nodes are in the same location in parameter space, node 1 will seize and node 2 will not seize because of bistability.*

Results, Possible mechanisms of recruitment, Paragraph 3 (Page 17, Line 392):

*In contrast to mechanism A, mechanism B can facilitate recruitment at any point in the burst in node 1 because it is a bifurcation along the route to node 1 that destabilizes node 2’s stable fixed point. Since the window for recruitment is longer in mechanism B, node 2 can be recruited through mechanism B at slower speeds than through mechanism A. Given that mechanism B does not rely on reaching node 1 before node 1 leaves its onset bifurcation, recruitment through mechanism B should also be less sensitive to coupling strength (G) and node 1 excitability (d*).*

21. In results the Authors state that dynamotypes in LCb recruit faster. Are there some statistics here? The figure suggests that many simulations of supH, for example, are faster than the other dynamotypes. I see more difference between each distribution in C, than in B (with C apparently demonstrating similarity). I would suggest some statistics are required here to support the claims.

We apologize for omitting statistical testing. As indicated below, we have added statistical support for our claims regarding recruitment proportion, delay, and energy. Note that, for results regarding recruitment proportion (Fig 5A), each datapoint now corresponds to the recruitment proportion for one node 2 location (calculated across all node 1 paths) as opposed to one node 1 path (calculated across all node 2 locations). This grouping increased the number of measurements from 6 to 71 so that we were better powered for statistical testing. Also, if we group recruitment by node 1 path, the measurements are not independent (measurements for dynamotypes with the same offset bifurcation are correlated, meaning we really have 8 groups of 3 as opposed to 4 groups of 6). Grouping by node 2 location results in better independence between measurements.

Model and methods, Recruitment analysis, Paragraph 2 (Page 14, Line 324):

*Statistical significance in recruitment properties between groups was evaluated via Kruskal-Wallis tests followed by multiple comparisons with Bonferroni correction, if appropriate.*

Results, Recruitment properties with respect to node 1 onset bifurcation, Paragraph 2-3

(Page 20, Line 469):

*Consistent with theoretical predictions, nodes in the LCb bistability region were more likely to recruit than nodes in the LCs bistability region. SN(-DC)- and subH-onset dynamotypes, located in LCb, showed elevated recruitment (Fig 5A) compared to SN(+DC)- and supH-onset dynamotypes, located in LCs. Recruitment proportion was particularly high for subH-onset dynamotypes, and particularly low for supH-onset dynamotypes.* ***All differences in recruitment proportion were statistically significant (Kruskal-Wallis test p = 2.5x10-184; post-hoc testing with Bonferroni correction yielded p < 0.001 for all pairwise comparisons).*** *Thus, the model predicts that seizures that do not start with baseline shifts are more likely to spread than seizures that display baseline shifts at onset. Seizures with amplitude scaling at onset are unlikely to spread.*

*SN(+DC)-onset node 1 dynamotypes also demonstrated longer recruitment delays (Fig 5B) compared to all other dynamotypes* ***(Kruskal-Wallis test p < 2.2x10-308; p < 1x10-190 for all pairwise comparisons with Bonferroni correction including SN(+DC)-onset dynamotypes).*** *Hence, the model predicts that seizures that exhibit baseline shifts at onset spread more slowly than seizures that do not exhibit baseline shifts at onset. Recruitment energy was highest for SN(+DC)-onset node 1 dynamotypes, followed by SN(-DC)-onset dynamotypes, then subH-onset dynamotypes, and finally lowest for supH-onset dynamotypes (Fig 5C).* ***All differences were statistically significant (Kruskal-Wallis test p < 2.2x10-308; p < 1x10-31 for all pairwise comparisons with Bonferroni correction).***

Results, Recruitment properties with respect to node 2 initial location, Paragraph 1

(Page 22, Line 527):

***The initial location of node 2 in parameter space also significantly influenced recruitment properties (Kruskal-Wallis tests yielded p<6.01x10-7 for comparisons of recruitment proportion, delay, and energy across node 2 initial locations).*** *For most node 2 initial locations, recruitment proportion was consistently low (Fig 7). Recruitment proportions increased and more node 2 initial locations were recruited at high excitability and coupling strength values (S2 Fig). Generally, recruitment delays and energy were consistent and decreased moving from near the LCs region to near the LCb region (Fig 7). These results are consistent with recruitment via mechanism A. Recall that in mechanism A, node 2 reaches node 1 in parameter space before node 1 leaves its onset bifurcation. Thus, recruitment through mechanism A is likely to be low and increase with node 1 excitability and coupling strength. Further, recruitment delay and energy are stereotyped because node 2 can only be recruited in a narrow timespan.*

22. Several points of clarity are required to make the article more accessible, here are some examples:

"In the propagation model, node 2 is initially placed at rest…" Presumably this should be a stable stationary point, since an unstable stationary point would be potentially "intrinsically epileptogenic", e.g. if in reality it would escape to a limit cycle?

Updated as shown below. Also, for clarity, we have changed the ‘resting’ region label in parameter space to ‘resting only’.

Model and methods, Connecting seizure onset and propagation zones, Paragraph 4 (Page 11, Line 256):

*In the propagation model, node 2 is initially placed on a stable fixed point in a resting-only or bistable region of parameter space and does not move autonomously.*

Can you please make sure all subscripts are defined (x_1 etc)

We thank the reviewer for noting we missed some subscripts, and we have fixed this.

The figures showing movement in parameter space require direction arrows to indicate the path through time.

Done to the extent possible.

Figure 3a is difficult to interpret since the markers overlap. It is also impossible to see the bistable regions indicated in the legend. Maybe a zoom would help? I found myself having to check back to previous figures in which I could see different details of the parameter map.

Fig. 3 is now in S1 Supplementary Methods. We have removed the markers from panel A and use panel A only to show where we are zooming in in panels C and D.

"Note that only parts of the SN and supH bifurcations can induce bursting" is confusing, for example what is a part of a bifurcation?

Changed to:

Model and methods, Possible mechanisms of recruitment, Paragraph 1
(Page 15, Line 356):

*Note in Fig 3A that only certain portions of the SN and supH bifurcation curves (those that are highlighted) can induce bursting.*

What is an "unmodified" hysteresis loop burster?

We meant that our implementation was the same as that previously published. Since this is confusing, we removed the word “unmodified.”

Why did you stipulate that each simulation should have 10 peaks (spikes), when the focus was on seizure onset?

When node 2 was not recruited (no burst), it was possible to observe transient responses (e.g. slow change in baseline, 1-2 spikes) at the onset and offset of coupling. We required 10 peaks in node 2 to quickly filter out these non-burst responses. We added the following explanation to the relevant methods section, now located in S1 Supplementary Methods:

S1 Supplementary Methods, Page 7, Paragraph 1

*Simulations with at least 10 peaks (spikes) in node 2 were considered for potential recruitment. Removing simulations with less than 10 peaks in node 2 filtered out simulations with only transient responses at the onset and/or offset of coupling.*

Why does the SN(+DC)/SupH path in Figure 3c extend to the SN point? This is a more complex burster than the other types since it crosses 3 bifurcation points. Why does the trajectory extend beyond the offset point in this and the SupH/SupH case?

In the hysteresis-loop implementation, supH bifurcations must be paired with SN bifurcations (see Izhikevich 2000, 2007 and Saggio et al., Journal of Mathematical Neuroscience, 2017). Dynamotypes with a supH bifurcation may have paths that extend beyond the onset/offset points for this reason. Note that the supH bifurcation is still the onset/offset bifurcation in the sense that it is the bifurcation responsible for transitions between stable fixed point and stable limit cycle. This is indeed a slightly more complex path. We actually used this criterion of ‘how many bifurcation curves need to be crossed’ as a way to stratify burster’s complexity in our previous work (Saggio et al., Journal of Mathematical Neuroscience, 2017). However, in analyzing the figures for this paper we realized that many human seizures demonstrate a DC shift, followed by a prolonged flat region (known as desynchronization to clinicians), followed by steadily rising amplitude spikes at onset, which is consistent with a SN(+DC) followed by supH-onset dynamotype (e.g. Fig 11B). While we cannot prove that such a pattern is the true SN(+DC) to supH bifurcation, it is remarkably similar to the model data.

For this first consideration of how diverse dynamics impact seizure propagation, we chose to isolate the supH bifurcation by adjusting the initial conditions for the simulation. This enabled the study of the recruitment properties of the supH bifurcation alone. We also considered SN followed by supH-onset dynamotypes separately; that analysis is now in S2 Supplementary Results. The offset bifurcation of node 1 was not a factor of interest in this work. We have added a sentence to the caption of Fig. 3, now in S1 Supplementary Methods, stating: *some bursting paths extend beyond the onset/offset points to close the hysteresis loop.*
