# Supplementary material for: Seizure recruitment properties are dependent upon dynamotype: A modeling study
Source: PLoS Comput Biol. 2026 Sep 21;22(9):e1013975. doi: 10.1371/journal.pcbi.1013975 (PMC13626473; doi:10.1371/journal.pcbi.1013975)
Supplement: Supplementary file 4 [file pcbi.1013975.s010.docx]

**Response to the Reviewers**

We would like to thank the reviewers for their detailed feedback on our manuscript. Please see our detailed responses below.

**Note on Code Availability**

We have updated our code. Specifically, file names have been changed to match figure numbers. We also added statistical analysis to the files corresponding to Fig 5 and Fig 6. Minor changes have been made to the code for S7 Fig in S2 Supplementary Results. We will upload the revised code with those minor changes to Deep Blue Data Repository when we know no further changes are required. In case any of the reviewers would like to access the updated code, we previously uploaded the scripts corresponding to the figures with our resubmission in S1 Code. All other code was unchanged and can be found using the provided link to Deep Blue Data Repository.

**Reviewer #3:**

1. I remain unconvinced that the work offers insight into the mechanisms of seizures. For example, I am unsatisfied by the response to the question of requirement for hysteresis. An external slow mechanism would itself be deterministic and cover offset not governed by fast activity. The model framework is phenomenological, therefore it is difficult to argue against another phenomenological component.

First, we must point out that this work is not focused on exploring the mechanisms of seizures, but rather propagation within the context of seizure dynamics, which is an underexplored area of research. The conclusions of this paper regard the response of node 2, which is governed by coupling to node 1 rather than the effect of hysteresis in node 1. The important limitation is that constraining to hysteresis loops limited the number of dynamotypes and pathways that could be simulated. Pathways that were not possible with hysteresis-loops included those that enter the ictal-only region of parameter space. For these pathways, we would expect node 2 to start bursting as soon as it crossed into the ictal-only region, similar to what occurs when node 2 enters the ictal-only region in several of the simulations. The effect is shown directly in Fig. 8C, graphically in S1_Fig B Active Rest, and several were included in the statistical analyses in S2_Fig).

Regarding the hysteresis, we thank the reviewer for insisting on this point. The reviewer is correct that the hysteresis-loop is not the only method capable of creating seizures with deterministic offset, and that an external control mechanism could potentially be implemented to generate autonomous seizures in the absence of hysteresis. We have sharpened our discussion of the choice to use hysteresis-loops and acknowledge areas for future work.

The main focus of this work is on propagation from an autonomous seizure onset zone within the established framework in the literature. Our method of propagation/coupling between the two nodes was to incorporate fast-slow coupling. Thus, it was logical to also incorporate fast-slow coupling into the seizure generation mechanism of Node 1. In the Multiclass Epileptor, there are two established methods for generating seizures: hysteresis-loops and slow-waves. Only the hysteresis-loop implementation exploits fast-to-slow feedback to enable autonomous seizures. Note that the use of fast-slow feedback for seizure generation is not isolated to the Multiclass Epileptor. Biophysical neural mass models for autonomous seizures, which incorporate slow dynamics (e.g. Lopez-Sola et al., Journal of Neural Engineering, 2022; Gentiletti et al., eLife, 2022; Rabuffo et al., eLife, 2025) support a key role for fast-slow feedback. These models incorporate multiple slow variables with fast-to-slow feedback, suggesting fast-to-slow feedback is physiologically important. (We note that bifurcation analysis of these biophysical models is complicated by their high dimensionality, so the question remains as to whether the slow dynamics are of hysteresis-loop type). It may be possible to phenomenologically incorporate fast-to-slow feedback in ways other than through hysteresis-loops in the Multiclass Epileptor, but no such methods are established in the literature.

Further, we are aware of two other phenomenological models for autonomous seizures, used to study the propagation of focal seizures in networks, with bifurcations as the dynamical mechanism for seizure onset: the Epileptor and the model used by Hebbink et al., Epilepsia, 2017. Both rely on hysteresis-loop slow dynamics, which allows a minimal implementation of fast-to-slow feedback with one slow variable. The role of hysteresis in these phenomenological models, especially in the Epileptor, which is a well-established model for the study of seizure propagation (e.g. Jirsa et al., Brain, 2014; Proix et al., Brain, 2017; Wang et al., Science Translational Medicine, 2023; Zhou et al., IEEE Transactions on Neural Systems and Rehabilitation Engineering, 2025) justifies our modeling choice for the current work.

Finally, restricting node 1 pathways to those between onset and offset bifurcations and contained in the bistability region (which is how hysteresis-loop pathways are defined) provides an important constraint for exploring parameter space. In particular, because of equivalence of the bursting produced along any path joining a given onset and offset bifurcation and contained in the bistability region (equivalence in terms of Izhikevich’s classification), we can further restrict to the shortest arc between the onset and offset bifurcations. The parameter space of the Multiclass Epileptor is dynamically rich, enabling the study of how different onset bifurcations impact recruitment. However, without any constraints on the node 1 path, the uncountable number of potential paths becomes impossible to explore. For this initial work, it was useful to focus only on the simplest paths – arcs between onset and offset bifurcations.

We have updated the discussion to address the reviewer’s comment, as shown below. We also updated our discussion of the hysteresis-loop implementation in S1 Supplementary Methods.

Discussion, Rationale for model development, paragraph 3 (page 29, line 704).

*There are multiple established methods for seizure generation in the Multiclass Epileptor [13, 28]. For this work, we chose to model the seizure onset zone using the hysteresis-loop implementation, which is the dynamical mechanism exploited by the well-established Epileptor model [11, 13, 24, 25, 27, 32, 41]. The advantages of the hysteresis-loop implementation include that it produces autonomous seizures with generation and termination that rely on feedback from fast activity (see S1 Supplementary Methods for expanded discussion). The strategy of fast-slow feedback is not unique to these phenomenological models: fast-to-slow feedback also tends to play a role in biophysically-inspired neural mass models for autonomous seizures [42-44]. The presence of hysteresis-loop slow dynamics in such high-dimensional models is a more complex question that still needs to be investigated. It is important to note that the primary results regard the pathway of node 2, which is not directly dependent on the implementation of node 1. However, using the hysteresis-loop implementation constrained the number of node 1 pathways and dynamotypes tested. In particular, node 1 did not enter the ictal-only region of parameter space. In the case in which node 1 paths move through the ictal-only region, we would expect node 2 to burst as soon as it is recruited into the ictal-only region (similar to the example in Fig 8B).*

S1 Supplementary Methods, page 2, paragraph 2 (new sentence bolded).

*Multiple realizations of the slow subsystem are possible [1,3,4]. In particular, hysteresis-loop implementations exploit bistability in the fast subsystem and feedback among fast and slow variables to produce autonomous bursting [12]. These paths capture the endogenous mechanisms that move the brain towards seizure termination upon onset, which occurs in all cases except status epilepticus [2]. The statistics of seizure termination support an underlying deterministic process [13]. In contrast, slow-wave implementations, while deterministic, lack this feedback, may occur without bistability, and require slow variables to change independently to force the fast ones into bursting. The complexity of slow-wave implementations is also higher than that of hysteresis-loop implementations, requiring a minimum of two slow variables instead of one. For the present work, we chose hysteresis-loop paths for the node representing the active seizure focus (node 1), enabling node 1 to start and end a seizure autonomously. This is the simplest choice that enables seizures that progress towards termination through feedback from the fast activity.* ***Also, in the hysteresis-loop implementation, paths can be realized as arcs between onset and offset bifurcations, which is a useful constraint for this initial work.*** *A single implementation was chosen for comparison among bursting paths.*

2. Regarding parameter changes on arcs and the speed of movement on the arc, the authors do not address my point about the influence on any conclusions drawn. For example, the authors state that paths that traverse the same sequence of regions are equivalent, but paths that are not constrained to an arc would permit additional sequences of crossings in and out of the different regions (essentially nullifying the particular arrangement of regions on the sphere).

The reviewer is correct in stating that unconstrained paths could permit additional bifurcation sequences for node 2. Our conclusions are based on an important assumption: that node 2 tends to move towards node 1 in parameter space. Under this assumption, the key results will be robust to different strategies for parameter changes and speed. We state and provide justification for the assumption on page 10, paragraph 2, lines 221-240 (Model and methods, ‘Connecting seizure onset and propagation zones’) of the manuscript. However, without that assumption, the pathways for node 2 are unconstrained and could take any arbitrary path, which would have a wide range of different effects.

Specifically, our theoretical predictions (page 17, paragraph 2, line 404, ‘Theoretical predictions’) depend strongly on the relative locations of regions on the sphere, in particular on the immediate neighboring regions of a given node 1’s onset bifurcation curve, and not on the path that node 2 traverses to reach node 1. First, under equal coupling strengths, we expect nodes in the LCb bistability region to recruit node 2’s from more initial locations than nodes in the LCs bistability region because the onset bifurcation curves in the LCb region are fully embedded in the ictal-only region. So long as node 2’s path is not biased against entering the ictal-only region (for which we can think of no supportive reasoning), this prediction is independent of the path node 2 traverses on the way to node 1. Further, we predicted that nodes in active rest were more likely to be recruited than nodes in rest (for a given combination of node 1 dynamotype and coupling strength) due to their proximity to the ictal-only region and nature of the state space. Both of these arguments would apply even if node 2 was permitted to move along a wandering or indirect path to node 1. Node 2 would be more likely to wander into the ictal-only region from active rest than rest due to the proximity of active rest to the ictal-only region. Finally, we show that the onset dynamics of the propagation zone may differ from or be the same as those of the seizure onset zone, depending on whether recruitment is through mechanism A or B. Mechanisms A and B are applicable regardless of node 2 path type.

However, we cannot predict how numerical findings regarding the speed and energy of recruitment may change if node 2 reaches node 1 via a different path. That is a subject for future work.

Regarding speed of movement of node 2 along the arc, our primary results were with one speed (${\dot{z}=G(x_{1}-x_{1, rest})}^{2}$). However, we repeated our simulations with an alternative speed ($\dot{z}=G\sqrt{\left( x_{1}-x_{1, rest} \right)^{2}+\left( y_{1}-y_{1, rest} \right)^{2}}$) and found no qualitative differences in the results (Figs S10 and S11 in Supplementary Results). The only potential changes with other speeds are that slower speed would make it more likely for node 2 to not seize (due to node 1 finishing before node 2 reached it), and that faster speed would reduce the delay for node 2 to seize. These would not change the conclusions of the paper unless biased toward faster speeds for some dynamotypes and not others.

We have modified the discussion to reflect the above points. The relevant paragraphs now read:

Discussion, Rationale for model development, paragraphs 5 and 6 (page 31, line 743).

*The novelty of our implementation is that, during coupling, the dynamics (parameter values) of the potential propagation zone are altered towards that of the onset zone. For this first exploration of recruitment among nodes with diverse dynamics, we moved node 2 along an arc path to node 1. This is the simplest method and assumes that node 2 moves in the shortest path toward node 1. However, it is possible that node 2 would take other pathways, which would not necessarily follow the same sequences of bifurcation crossings nor necessarily follow an arc path. However, even in the context of more wandering paths, many of our results are still applicable. The two mechanisms of recruitment are independent of the path node 2 travels to reach node 1. Theoretical predictions regarding the likelihood of recruitment rely on the relative arrangement of regions in parameter space, in particular on the immediate neighboring regions of a given node 1’s onset bifurcation curve, and are expected to be robust to path. More complex paths may enable more complicated propagation zone behavior by crossing additional bifurcations. We already found that node 2 onset behavior could be the same as or differ from that of node 1; more complex paths would increase the potential for variations in onset behavior. Conversely, quantitative results, particularly those regarding recruitment speed and energy, may be altered in consideration of alternative node 2 paths. We leave the study of more complicated routes, and the behaviors and numerical results that may arise, to future work.*

*As a first approximation, we chose the speed of movement of node 2 towards node 1 to be proportional to the square of the difference from rest in node 1’s x variable (Eqn. 3). In developing this coupling function, we reasoned that the novel activity in the seizure onset zone (i.e. the electric field generated, represented by the change in voltage: x_1_ – x_1, rest_) influences the propagation zone. The coupling is phenomenological and thus effectively summarizes the cumulative effect of a variety of processes which depend on the specific conditions and locations of the brain regions involved. We squared the change in voltage to avoid biasing recruitment towards bursting paths in LCs (which have a distinct separation between bursting and resting states; see [13]) compared to bursting paths in LCb (in which oscillations cross the resting state). To further ensure that the coupling function was not biasing results, we calculated the area under the entire coupling term G(x_1_ – x_1, rest_)^2^ during the period between node 1 and node 2 onset. We called this value the recruitment energy. If the coupling function biased recruitment towards certain dynamotypes, we would expect dynamotypes with elevated recruitment proportions to also demonstrate elevated recruitment energy. However, there was no consistent relationship between recruitment proportion and recruitment energy among node 1 onset bifurcations (Fig 5). We therefore infer that the differences in recruitment proportion are not due to biases or saturation effects introduced by the choice of coupling term. We also found no major difference in results with a different coupling function (S2 Supplementary Results). Nevertheless, other choices for the coupling term could be equally relevant and are an area for future study. Other coupling terms may change the speed of movement of node 2 toward node 1, which could influence the likelihood of recruitment and recruitment time.*
