# Supplementary material for: Seizure recruitment properties are dependent upon dynamotype: A modeling study
Source: PLoS Comput Biol. 2026 Sep 21;22(9):e1013975. doi: 10.1371/journal.pcbi.1013975 (PMC13626473; doi:10.1371/journal.pcbi.1013975)
Supplement: Peer review history

# Peer review: Decision Letter 0

**DOI:** 10.1371/journal.pcbi.1013975.r001

**Authors:** Thomas Serre, Jian Liu

13 Mar 2026

Seizure recruitment properties are dependent upon dynamotype: A modeling study

PLOS Computational Biology

Dear Dr. Stacey,

Thank you for submitting your manuscript to PLOS Computational Biology. After careful consideration, we feel that it has merit but does not fully meet PLOS Computational Biology's publication criteria as it currently stands. Therefore, we invite you to submit a revised version of the manuscript that addresses the points raised during the review process.

Please submit your revised manuscript by May 13 2026 11:59PM. If you will need more time than this to complete your revisions, please reply to this message or contact the journal office at ploscompbiol@plos.org. When you're ready to submit your revision, log on to https://www.editorialmanager.com/pcompbiol/ and select the 'Submissions Needing Revision' folder to locate your manuscript file.

Please include the following items when submitting your revised manuscript:

* A letter that responds to each point raised by the editor and reviewer(s). You should upload this letter as a separate file labeled 'Response to Reviewers'. This file does not need to include responses to formatting updates and technical items listed in the 'Journal Requirements' section below.

* A marked-up copy of your manuscript that highlights changes made to the original version. You should upload this as a separate file labeled 'Revised Manuscript with Track Changes'.

* An unmarked version of your revised paper without tracked changes. You should upload this as a separate file labeled 'Manuscript'.

If you would like to make changes to your financial disclosure, competing interests statement, or data availability statement, please make these updates within the submission form at the time of resubmission. Guidelines for resubmitting your figure files are available below the reviewer comments at the end of this letter

We look forward to receiving your revised manuscript.

Kind regards,

Jian Liu

Academic Editor

PLOS Computational Biology

Thomas Serre

Section Editor

PLOS Computational Biology

**Additional Editor Comments:**

This work has merit and addresses an interesting problem. However, substantial revisions are required to address the concerns raised by the reviewers. Please see their detailed comments and respond accordingly.

**Journal Requirements:**

1) We ask that a manuscript source file is provided at Revision. Please upload your manuscript file as a .doc, .docx, .rtf or .tex. If you are providing a .tex file, please upload it under the item type u2018LaTeX Source Fileu2019 and leave your .pdf version as the item type u2018Manuscriptu2019.

2) Please upload all main figures as separate Figure files in .tif or .eps format. For more information about how to convert and format your figure files please see our guidelines:

https://journals.plos.org/ploscompbiol/s/figures

3) We notice that your supplementary Figures, and Tables are included in the manuscript file. Please remove them and upload them with the file type 'Supporting Information'. Please ensure that each Supporting Information file has a legend listed in the manuscript after the references list.

4) Please amend your detailed Financial Disclosure statement. This is published with the article. It must therefore be completed in full sentences and contain the exact wording you wish to be published.

- State the initials, alongside each funding source, of each author to receive each grant. For example: "This work was supported by the National Institutes of Health (####### to AM; ###### to CJ) and the National Science Foundation (###### to AM)."

- State what role the funders took in the study. If the funders had no role in your study, please state: "The funders had no role in study design, data collection and analysis, decision to publish, or preparation of the manuscript.".

If you did not receive any funding for this study, please simply state: u201cThe authors received no specific funding for this work.u201d

**Reviewers' comments:**

Reviewer's Responses to Questions

**Comments to the Authors:**

**Please note here if the review is uploaded as an attachment.**

Reviewer #1: The manuscript introduces a parameter-space coupling mechanism to study seizure recruitment across multiple seizure dynamotypes using the Multiclass Epileptor (DBT Burster). The authors analyze and simulate two coupled nodes—a bursting onset zone (node 1) and a non-epileptogenic potential propagation zone (node 2)—to determine how coupling strength, excitability, and the specific onset bifurcation in node 1 shape recruitment likelihood, delay, and onset morphology in node 2. The study provides a theoretical account of two recruitment mechanisms, performs an extensive simulation sweep, predicts that dynamotypes with baseline shifts and those with amplitude-scaling onsets are less prone to propagate, and spread more slowly, and presents human intracranial EEG examples consistent with several predicted “mismatched” onset behaviors between onset and recruited nodes. It leverages the Multiclass Epileptor to explicitly interrogate how onset bifurcation class (SN(+DC), supH, SN(−DC), subH) influences recruitment, an aspect often homogenized in prior whole-brain modeling, and introduces a conceptually original coupling method that moves the propagation node along geodesics in the dynamical parameter space toward the onset node, enabling heterogeneous local dynamics across regions. The results provide a theoretical framing with two recruitment mechanisms (A and B) grounded in the geometry of the DBT bursting landscape and bifurcation topology. Methods are carefully documented and assumptions (e.g., one-directional coupling, hysteresis-loop for node 1, slow-wave for node 2) are articulated for the modeling choices. The manuscript addresses an important gap by examining dynamotype-dependent propagation, informing potential interpretation of seizure networks and aiding delineation of onset vs propagation zones. The framework is general to fast–slow bursters and could be extended to more realistic networks and couplings, potentially supporting standardized quantification of propagation patterns beyond latency/synchrony heuristics. However, there are still some concerns which should be clarified for publication.

1. No stochasticity or measurement noise is incorporated; robustness of recruitment predictions to realistic noise remains untested.

2. Human data serve primarily as proof-of-concept illustrations. Validation on human data is qualitative and limited to a few examples; no quantitative assessment (e.g., rates of predicted mismatches, delay distributions, or dependence on onset patterns) is provided.

3. The coupling function for node 2’s slow variable is ad hoc (proportional to (x1 − x1,rest)^2), with no comparison to alternative fast-to-slow or slow-to-slow couplings, saturation effects, or normalization across dynamotypes.

4. Mechanism A can require temporary violation of fast–slow timescale separation, but the implications of this assumption for model validity are not deeply examined.

5. The return dynamics for node 2 (constant speed k2) may bias post-recruitment trajectories; physiological plausibility of this choice is not established.

6. Requires a deeper comparison to Epileptor field modeling of propagation and recruitment mechanisms (e.g., Proix et al., 2017), including differences between fast vs slow permittivity couplings and the role of LVFA-mediated coupling suppression.

7. Limited discussion of how this framework complements patient-specific TVB-based recruitment models and emerging multi-scale co-simulation approaches (e.g., Arbor–TVB), or analytic advances in neural mass model inversion across phase-space regimes.

Reviewer #2: Summary

In this manuscript, the authors utilize a dynamical modeling approach, specifically the Multiclass Epileptor, to investigate how the underlying dynamical mechanisms (dynamotypes) of a seizure onset zone influence the recruitment and propagation of seizures to secondary brain regions. The authors conclude that certain seizure onset bifurcations (e.g., those with baseline shifts) are less likely to spread. While the mathematical exploration of node-to-node coupling of varied dynamotypes represents a theoretical step forward, the manuscript in its current form suffers from a severe disconnect between its theoretical models and biological reality. Furthermore, the clinical validation is highly anecdotal, and the manuscript lacks the statistical rigor necessary to support its primary claims. Significant structural and substantive revisions are required.

Major Comments

1. Weak Link Between Model Assumptions and Biological Reality

The primary weakness of this study is the tenuous link between the mathematical assumptions of the Multiclass Epileptor and physiological reality. The phenomenological nature of the model eclipses the theoretical advances the paper claims to make. For instance, the model predicts that seizures with a baseline shift at onset are less likely to spread. However, without a clear mapping of what a "baseline shift" or specific bifurcation means in terms of underlying tissue excitability, ion concentrations, or synaptic kinetics, this prediction lacks biological grounding. In the clinical reality, a baseline shift can be a signal artifact, might depend on the recording location, electrode type and amplifier settings, with extensive literature in neurophyiology discussing this decades ago. Consequently, the clinical relevance of the model's predictions is highly questionable. The authors bury several key model assumptions in the text, making it difficult to assess the validity of their claims.

2. Lack of Statistical Rigor and Anecdotal Clinical Validation

The manuscript makes quantitative-sounding claims—such as certain dynamotypes being "less likely to spread" or spreading more slowly—but fails to support these assertions with statistical rigor. The clinical and real-life data section relies on anecdotal, cherry-picked examples of human intracranial EEG (iEEG) traces to support "unusual combinations" of seizure dynamics. Without systematically quantifying these occurrences across a sufficiently powered clinical dataset, the premise that the model reflects real-world seizure dynamics remains unsubstantiated. Claims regarding the probability of seizure spread must be backed by appropriate statistical testing.

3. Omission of Anatomical Constraints in Propagation

In evaluating the iEEG data, the authors fail to acknowledge the profound role that brain anatomy and structural connectivity play in seizure propagation at the cellular and network level. Seizure spread in the human brain is not merely a function of abstract node-to-node dynamical coupling; it is heavily constrained by white matter tracts, spatial proximity, local circuitry, and network topology. Indeed if the authors could link dynamotypes to anatomical locations and their onset and spread pattern in clinical data, that would form a far more convincing paper.

4. Target Audience and Theoretical Contribution

The paper struggles to find its audience. For experts in dynamical systems, the insights into coupled oscillators and bifurcations may appear trivial or derivative of existing nonlinear dynamics literature. Conversely, for the non-expert (e.g., clinical epileptologists or systems neuroscientists), the dense mathematical focus and lack of biological translation make the paper impenetrable. The authors need to clearly articulate the novel biological insight generated by this mathematical exercise.

5. Manuscript Structure, Length, and Clarity

The manuscript is excessively long and lacks a cohesive narrative arc.

Obscured Main Results: The primary findings are difficult to locate amidst dense, poorly structured sections.

Figure Clarity: The figures and their captions are overly abstract. They do not guide the reader toward a cohesive story or clearly illustrate the biological equivalents of the mathematical phenomena.

Need for Streamlining: Entire sections detailing the mathematical derivations or exhaustive parameter sweeps should be moved to the Supplementary Material to allow the main text to focus on the core biological hypotheses and results.

Recommendation: Major Revision (or Reject, with encouragement to resubmit a substantially restructured manuscript). In my opinion, to be considered suitable for publication at a computational biology journal, the authors must explicitly link their abstract bifurcations to some level of biological mechanisms, provide statistical validation for their claims using a comprehensive clinical dataset, account for anatomical constraints, and drastically streamline the text to highlight a clear, biologically relevant main result.

Reviewer #3: The Authors use a phenomenological model of bursting dynamics, the epileptor, to study dynamics of propagation of seizures. They introduce a new model of coupling between epileptor nodes motivated by the observation that dynamics in seizure onset and propagation regions can be different. They produce a thorough analysis of the different kinds of propagation dynamics that can occur when different onset dynamics are employed in the seizure generating node. I thought that the manuscript was unclear in places and I was not entirely convinced that we can gain insight into the brain from this approach, but am open to be convinced otherwise. Please see some details below.

As in all modelling studies, several assumptions are made regarding the choice of model. The epileptor has been a popular choice and is worth further exploration, but is phenomenological. I think this makes it difficult to justify some of the choices made here:

- It is unclear to me why the Authors focus on hysteresis loops for onset and offset of the seizure dynamics. Why do they exclude the case of moving in and out of the ictal only regime from active rest or rest, i.e. the slow wave mechanism they mention. The model is phenomenological, therefore placing restrictions such as generation of "autonomous" seizures seems arbitrary. Could the Authors explain in further detail why some autonomous mechanism cannot be achieved in the slow wave scenario, for example by implementing an autonomous control mechanism using the amplitude of the simulated signal?

- In terms of onset and offset of seizures, why would we assume that changes in parameter values are restricted to an arc between onset and offset points? Is there a biological reason for this, or could more complicated or "wandering" paths occur? This could affect the conclusions that are drawn, e.g. about the relationship between seizure onset dynamics and propagation dynamics.

- Similarly, equation 9 prescribes a specific form for the speed of movement along the arc. Looking at the supplemental video, it seems that G is doing most of the work here, since attraction of node 2 to the position of node 1 is almost instantaneous. What are the biophysical motivations for the choice of equation 9? Why would the biophysical mechanisms of recruitment unfold in this way? This affects some of the conclusions that are drawn, e.g. in the description of mechanism A, it is unclear why recruitment should occur early in the burst in node 1. This would simply depend on the dwell time in each part of the path of node 1 from onset to offset. The Authors state that the form of coupling captures the influence of the change in voltage in the seizure onset zone on the activity of the propagation zone. Could they please clarify what this influence is, biophysically?

- In motivating the form of coupling chosen the authors argue that the propagation zone often exhibits the same onset patterns as the seizure onset zone. This could be achieved in the model by implementing some form of synaptic driving: e.g. the node 2 is in a rest state and simply responds to the oscillations of node 1. This would not always lead to identical dynamics in both nodes, due to the dynamics of the response in node 2 and any delays etc. Therefore I do not follow the logic in the passage beginning "First, the highly excitable onset zone… ". Node 2 could be in any region of resting dynamics for this to occur (i.e. wouldn't need to be in the same part of this space as node 2). As an aside, I am unsure how much relevance to place on the size of these different regions since the model is phenomenological: we have no reference point for which regions of the parameter space it could or could not inhabit, nor the relative sizes of these. In this same section the authors state ".. seizures often propagate to brain regions that are not independently epileptogenic". How do we know this is the case? How could we verify that a propagation zone is not inherently epileptogenic? Presumably by looking into post-surgical seizure freedom over a sufficient time scale?

- In terms of the theoretical prediction: I'm not sure what "more likely to recruit" means: each node 2 will follow its path through to the spiking dynamics, given sufficient coupling and speed. Also, that nodes in active rest are more likely to be recruited than nodes in rest. I guess there is an implicit assumption here that the relative size of the parameter space is meaningful. It would be helpful if these assumptions were spelled out and justified.

- "The variability of ictal inter-spike intervals across bursting paths was confirmed to be within the expected physiological range": Since the time scale in the model is arbitrary, what is the reference point for this?

"Given that mechanism B does not rely as heavily on node 2's speed".. I'm not sure I follow: speed is still important since node 2 has to be moved across bifurcations.

In results the Authors state that dynamotypes in LCb recruit faster. Are there some statistics here? The figure suggests that many simulations of supH, for example, are faster than the other dynamotypes. I see more difference between each distribution in C, than in B (with C apparently demonstrating similarity). I would suggest some statistics are required here to support the claims.

Several points of clarity are required to make the article more accessible, here are some examples:

"In the propagation model, node 2 is initially placed at rest…" Presumably this should be a stable stationary point, since an unstable stationary point would be potentially "intrinsically epileptogenic", e.g. if in reality it would escape to a limit cycle?

Can you please make sure all subscripts are defined (x_1 etc)

The figures showing movement in parameter space require direction arrows to indicate the path through time.

Figure 3a is difficult to interpret since the markers overlap. It is also impossible to see the bistable regions indicated in the legend. Maybe a zoom would help? I found myself having to check back to previous figures in which I could see different details of the parameter map.

"Note that only parts of the SN and supH bifurcations can induce bursting" is confusing, for example what is a part of a bifurcation?

What is an "unmodified" hysteresis loop burster?

Why did you stipulate that each simulation should have 10 peaks (spikes), when the focus was on seizure onset?

Why does the SN(+DC)/SupH path in Figure 3c extend to the SN point? This is a more complex burster than the other types since it crosses 3 bifurcation points. Why does the trajectory extend beyond the offset point in this and the SupH/SupH case?

**********

**Have the authors made all data and (if applicable) computational code underlying the findings in their manuscript fully available?**

The PLOS Data policy requires authors to make all data and code underlying the findings described in their manuscript fully available without restriction, with rare exception (please refer to the Data Availability Statement in the manuscript PDF file). The data and code should be provided as part of the manuscript or its supporting information, or deposited to a public repository. For example, in addition to summary statistics, the data points behind means, medians and variance measures should be available. If there are restrictions on publicly sharing data or code —e.g. participant privacy or use of data from a third party—those must be specified.

Reviewer #1: Yes

Reviewer #2: None

Reviewer #3: None

**********

PLOS authors have the option to publish the peer review history of their article (what does this mean?). If published, this will include your full peer review and any attached files.

If you choose “no”, your identity will remain anonymous but your review may still be made public.

**Do you want your identity to be public for this peer review?**  For information about this choice, including consent withdrawal, please see our Privacy Policy.

Reviewer #1: **Yes:** Dongping Yang

Reviewer #2: No

Reviewer #3: No

[NOTE: If reviewer comments were submitted as an attachment file, they will be attached to this email and accessible via the submission site. Please log into your account, locate the manuscript record, and check for the action link "View Attachments". If this link does not appear, there are no attachment files.]

**Figure resubmission:**

After uploading your figures to PLOS’s NAAS tool - https://ngplosjournals.pagemajik.ai/artanalysis, NAAS will process the files provided and display the results in the "Uploaded Files" section of the page as the processing is complete. If the uploaded figures meet our requirements (or NAAS is able to fix the files to meet our requirements), the figure will be marked as "fixed" above. If NAAS is unable to fix the files, a red "failed" label will appear above. When NAAS has confirmed that the figure files meet our requirements, please download the file via the download option, and include these NAAS processed figure files when submitting your revised manuscript.

**Reproducibility:**

?>

# Author comment: Author response to Decision Letter 1

**DOI:** 10.1371/journal.pcbi.1013975.r002

29 May 2026

Attachment Submitted filename: ResponsetoReviewers.docx

# Peer review: Decision Letter 1

**DOI:** 10.1371/journal.pcbi.1013975.r003

**Authors:** Thomas Serre, Jian Liu

20 Jul 2026

PCOMPBIOL-D-26-00260R1

Seizure recruitment properties are dependent upon dynamotype: A modeling study

PLOS Computational Biology

Dear Dr. Stacey,

Thank you for submitting your manuscript to PLOS Computational Biology. After careful consideration, we feel that it has merit but does not fully meet PLOS Computational Biology's publication criteria as it currently stands. Therefore, we invite you to submit a revised version of the manuscript that addresses the points raised during the review process.

Please submit your revised manuscript by Sep 19 2026 11:59PM. If you will need more time than this to complete your revisions, please reply to this message or contact the journal office at ploscompbiol@plos.org. When you're ready to submit your revision, log on to https://www.editorialmanager.com/pcompbiol/ and select the 'Submissions Needing Revision' folder to locate your manuscript file.

Please include the following items when submitting your revised manuscript:

* A letter that responds to each point raised by the editor and reviewer(s). You should upload this letter as a separate file labeled 'Response to Reviewers'. This file does not need to include responses to formatting updates and technical items listed in the 'Journal Requirements' section below.

* A marked-up copy of your manuscript that highlights changes made to the original version. You should upload this as a separate file labeled 'Revised Manuscript with Track Changes'.

* An unmarked version of your revised paper without tracked changes. You should upload this as a separate file labeled 'Manuscript'.

If you would like to make changes to your financial disclosure, competing interests statement, or data availability statement, please make these updates within the submission form at the time of resubmission. Guidelines for resubmitting your figure files are available below the reviewer comments at the end of this letter.

As the corresponding author, your ORCID iD is verified in the submission system and will appear in the published article. PLOS supports the use of ORCID, and we encourage all coauthors to register for an ORCID iD and use it as well. Please encourage your coauthors to verify their ORCID iD within the submission system before final acceptance, as unverified ORCID iDs will not appear in the published article. Only the individual author can complete the verification step; PLOS staff cannot verify ORCID iDs on behalf of authors.

We look forward to receiving your revised manuscript.

Kind regards,

Jian Liu

Academic Editor

PLOS Computational Biology

Thomas Serre

Section Editor

PLOS Computational Biology

**Additional Editor Comments (if provided):**

There are some remaining questions that need to be addressed properly. Please revise the manuscript accordingly.

**Reviewers' comments:**

Reviewer's Responses to Questions

**Comments to the Authors:**

**Please note here if the review is uploaded as an attachment.**

Reviewer #1: Thanks for the detailed responses to my concerns. I have no more concern. It is now acceptable for publication.

Reviewer #3: I remain unconvinced that the work offers insight into the mechanisms of seizures. For example, I am unsatisfied by the response to the question of requirement for hysteresis. An external slow mechanism would itself be deterministic and cover offset not governed by fast activity. The model framework is phenomenological, therefore it is difficult to argue against another phenomenological component.

Regarding parameter changes on arcs and the speed of movement on the arc, the authors do not address my point about the influence on any conclusions drawn. For example, the authors state that paths that traverse the same sequence of regions are equivalent, but paths that are not constrained to an arc would permit additional sequences of crossings in and out of the different regions (essentially nullifying the particular arrangement of regions on the sphere).

**********

**Have the authors made all data and (if applicable) computational code underlying the findings in their manuscript fully available?**

The PLOS Data policy requires authors to make all data and code underlying the findings described in their manuscript fully available without restriction, with rare exception (please refer to the Data Availability Statement in the manuscript PDF file). The data and code should be provided as part of the manuscript or its supporting information, or deposited to a public repository. For example, in addition to summary statistics, the data points behind means, medians and variance measures should be available. If there are restrictions on publicly sharing data or code —e.g. participant privacy or use of data from a third party—those must be specified.

Reviewer #1: Yes

Reviewer #3: None

**********

PLOS authors have the option to publish the peer review history of their article (what does this mean?). If published, this will include your full peer review and any attached files.

If you choose “no”, your identity will remain anonymous but your review may still be made public.

**Do you want your identity to be public for this peer review?**  For information about this choice, including consent withdrawal, please see our Privacy Policy.

Reviewer #1: **Yes:** Dongping Yang

Reviewer #3: No

[NOTE: If reviewer comments were submitted as an attachment file, they will be attached to this email and accessible via the submission site. Please log into your account, locate the manuscript record, and check for the action link "View Attachments". If this link does not appear, there are no attachment files.]

**Figure resubmission:**

**Reproducibility:**

To enhance the reproducibility of your results, we recommend that authors of applicable studies deposit laboratory protocols in protocols.io, where a protocol can be assigned its own identifier (DOI) such that it can be cited independently in the future. Additionally, PLOS ONE offers an option to publish peer-reviewed clinical study protocols. Read more information on sharing protocols at https://plos.org/protocols?utm_medium=editorial-email&utm_source=authorletters&utm_campaign=protocols

# Author comment: Author response to Decision Letter 2

**DOI:** 10.1371/journal.pcbi.1013975.r004

26 Aug 2026

Attachment Submitted filename: ResponsetoReviewers 2.docx

# Editor report: Decision Letter 2

**DOI:** 10.1371/journal.pcbi.1013975.r005

**Authors:** Thomas Serre, Jian Liu

1 Sep 2026

Dear Dr. Stacey,

We are pleased to inform you that your manuscript 'Seizure recruitment properties are dependent upon dynamotype: a modeling study' has been provisionally accepted for publication in PLOS Computational Biology.

Before your manuscript can be formally accepted you will need to complete some formatting changes, which you will receive in a follow up email. A member of our team will be in touch with a set of requests.

Please note that your manuscript will not be scheduled for publication until you have made the required changes, so a swift response is appreciated.

IMPORTANT: The editorial review process is now complete. PLOS will only permit corrections to spelling, formatting or significant scientific errors from this point onwards. Requests for major changes, or any which affect the scientific understanding of your work, will cause delays to the publication date of your manuscript.

Should you, your institution's press office or the journal office choose to press release your paper, you will automatically be opted out of early publication. We ask that you notify us now if you or your institution is planning to press release the article. All press must be co-ordinated with PLOS.

Thank you again for supporting Open Access publishing; we are looking forward to publishing your work in PLOS Computational Biology.

Best regards,

Jian Liu

Academic Editor

PLOS Computational Biology

Thomas Serre

Section Editor

PLOS Computational Biology

***********************************************************

The authors have adequately revised the manuscript within the stated scope of the study. The work is now suitable for publication.

# Editor report: Acceptance letter

**DOI:** 10.1371/journal.pcbi.1013975.r006

**Authors:** Thomas Serre, Jian Liu

PCOMPBIOL-D-26-00260R2

Seizure recruitment properties are dependent upon dynamotype: a modeling study

Dear Dr Stacey,

I am pleased to inform you that your manuscript has been formally accepted for publication in PLOS Computational Biology. Your manuscript is now with our production department and you will be notified of the publication date in due course.

The corresponding author will soon be receiving a typeset proof for review, to ensure errors have not been introduced during production. Please review the PDF proof of your manuscript carefully, as this is the last chance to correct any errors. Please note that major changes, or those which affect the scientific understanding of the work, will likely cause delays to the publication date of your manuscript.

Soon after your final files are uploaded, unless you have opted out, the early version of your manuscript will be published online. The date of the early version will be your article's publication date. The final article will be published to the same URL, and all versions of the paper will be accessible to readers.

For Research, Software, and Methods articles, you will receive an invoice from PLOS for your publication fee after your manuscript has reached the completed accept phase. If you receive an email requesting payment before acceptance or for any other service, this may be a phishing scheme. Learn how to identify phishing emails and protect your accounts at https://explore.plos.org/phishing.

Thank you again for supporting PLOS Computational Biology and open-access publishing. We are looking forward to publishing your work!

With kind regards,

Janani Seenivasan

PLOS Computational Biology | Carlyle House, Carlyle Road, Cambridge CB4 3DN | United Kingdom ploscompbiol@plos.org | Phone +44 (0) 1223-442824 | ploscompbiol.org | @PLOSCompBiol
